# Aerobic Oxidative EDA Catalysis: Synthesis of Tetrahydroquinolines Using an Organocatalytic EDA Active Acceptor

**DOI:** 10.1021/acs.joc.1c02776

**Published:** 2022-01-10

**Authors:** August Runemark, Henrik Sundén

**Affiliations:** † Department of Chemistry and Chemical Engineering, Chalmers University of Technology, Kemivägen 10, 412 96 Gothenburg, Sweden; ‡ Chemistry and Molecular Biology, University of Gothenburg, Kemivägen 10, 412 96 Gothenburg, Sweden

## Abstract

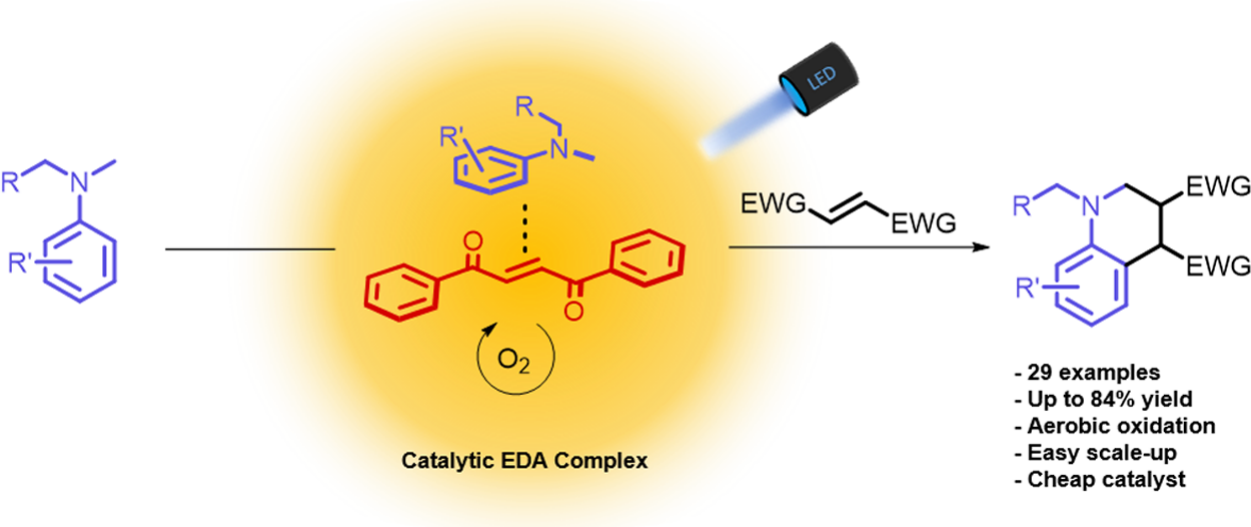

A catalytic electron donor–acceptor (EDA) complex for the visible-light-driven annulation reaction between activated alkenes and N,N-substituted dialkyl anilines is reported. The key photoactive complex is formed *in situ* between dialkylated anilines as donors and 1,2-dibenzoylethylene as a catalytic acceptor. The catalytic acceptor is regenerated by aerobic oxidation. Investigations into the mechanism are provided, revealing a rare example of a catalytic acceptor in photoactive EDA complexes that can give access to selective functionalization of aromatic amines under mild photochemical conditions.

## Introduction

An electron donor–acceptor (EDA) complex is a weak molecular aggregation between an electron-rich donor and an electron-poor acceptor.^1^ A characteristic of EDA complexes is their associated charge-transfer (CT) band in the electromagnetic spectrum. Excitation by light within this CT band results in a single electron transfer (SET) from the donor to the acceptor. The resulting radical species can subsequently undergo a range of different processes and take part in radical reactions. Due to the red-shifted absorption of the CT band, compared to the individual components of the EDA complex, visible light can often be used to induce reactivity in compounds otherwise not absorbing in this spectral region.^2^

**Scheme 1:**
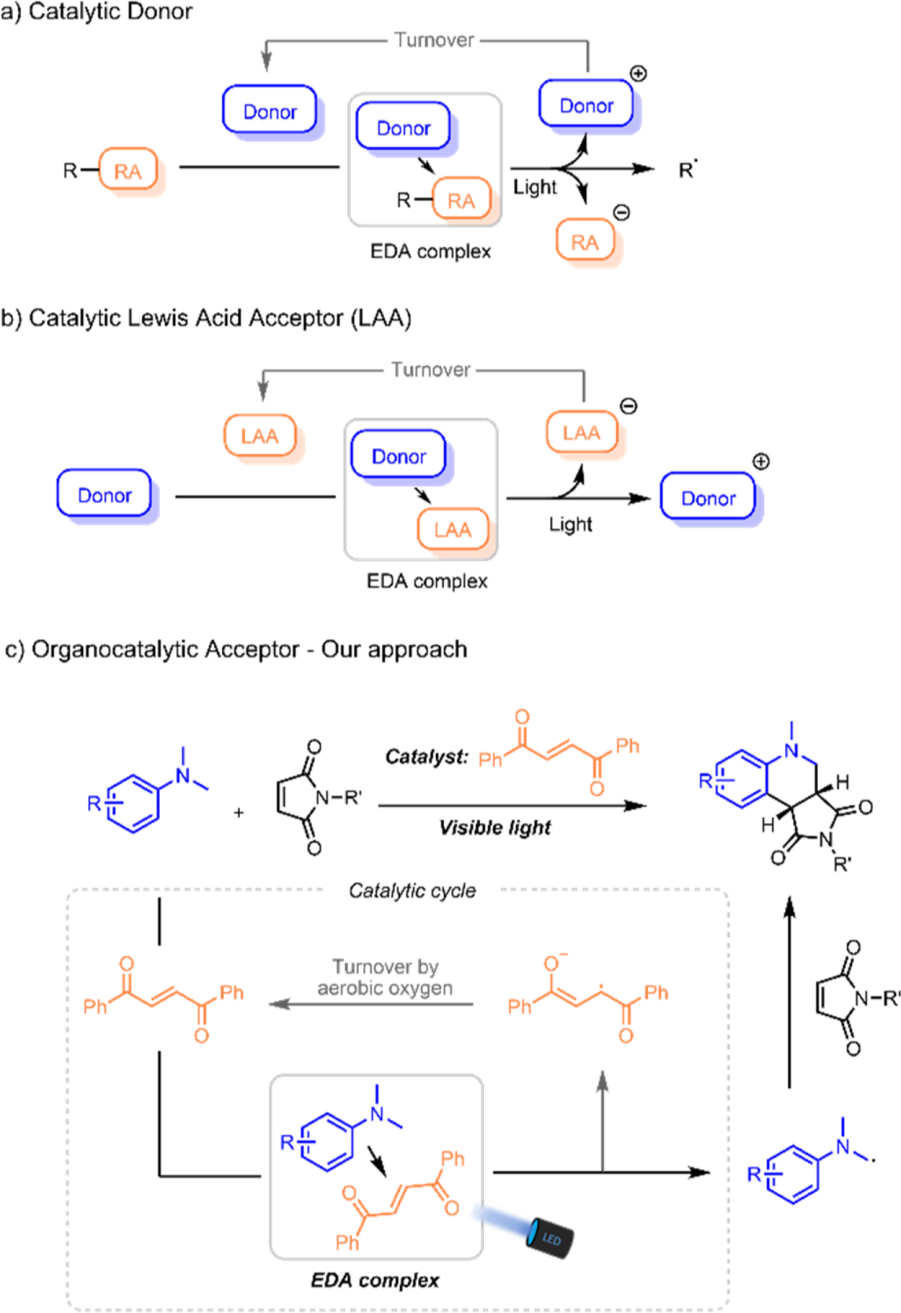
Examples of Catalytic EDA Complexes in Organic Synthesis (a) Working principle of a system with catalytic donors in combination with redox auxiliaries (RA). (b) Working principle of a system using Lewis acids as electron acceptors (LAA). (c) EDA catalysis for visible light-mediated synthesis of tetrahydroquinolines.

Over the last decade, a wide range of different approaches to utilize EDA complexes in synthetic organic chemistry have been developed.^1−4^ Examples include arylations,^5^ stereoselective alkylations,^6^ oxidative annulations,^7^ and acylations.^8^ The prototypical examples are coupling reactions between a donor and an acceptor, driven by the formation of a stoichiometric EDA complex,^7,9^ although strategies to harvest the synthetic potential of EDA complexes have rapidly been expanded to include catalytic complexes (Scheme 1).^2,10−12^

Within the catalytic regime, different approaches can be exploited depending on how the EDA complex is formed. The EDA donor or acceptor can be formed *in situ* from a pre-catalyst, generating highly polarized species from non-reactive substrates. Examples include *in situ*-formed enamines and enolates as donors, or iminium ions as acceptors, that take part in the formation of photoactive EDA complexes.^6,13,14^

Alternatively, an external donor can be present in catalytic amount that associates with a redox-active reactant to form an EDA complex (Scheme 1a). After a SET to the reactant, the donor can re-form to close the catalytic cycle. Examples of these systems include the use of electron-rich aromatics and amines.^10,11,15,16^ Examples of systems using catalytic acceptors in the same manner, however, still remain rare in the literature and are limited to strong Lewis acids (Scheme 1b).^17,18^ Identification of milder suitable acceptors that could act as catalysts for photo-mediated synthesis would constitute a significant contribution to the field of EDA complex-driven transformations. Inspired by this opportunity, we set out to investigate this methodology in the generation of α-amino alkyl radicals. Herein, we present a protocol using visible light to furnish a range of annulation products from aromatic amines and activated alkenes, driven by the formation of a photoactive organocatalytic acceptor–EDA complex (Scheme 1c).

## Results and Discussion

**Figure 1:**
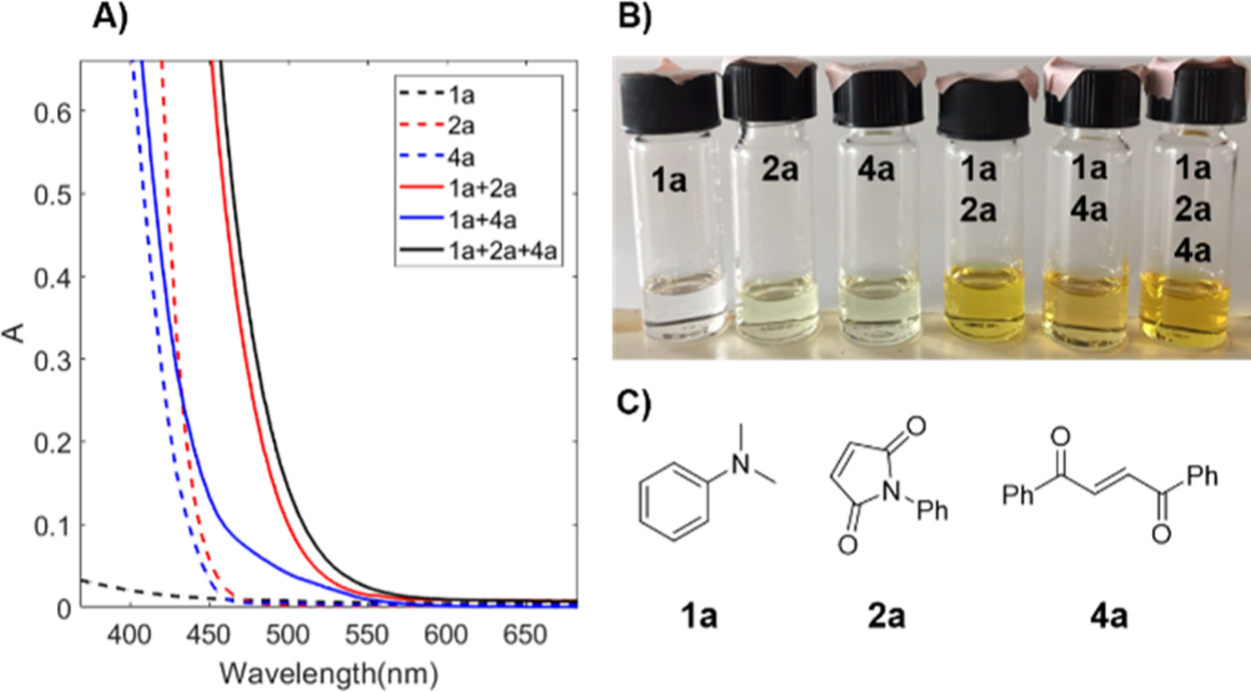
(A) UV–vis spectra of **1a** (0.4 M), **2a** (73 mM), **4a** (6 mM), and their EDA complexes in 1,4-dioxane; (B) pictures of **1a**, **2a**, **4a**, and their EDA complexes; and (C) structures of **1a**, **2a**, and **4a**.

As a model system for this study, 1,2-dibenzoyl ethylene **4a** as a catalyst in combination with *N*,*N*-dimethylaniline (DMA) **1a** was chosen because the EDA complex of the two species absorbs strongly in the visible region (Figure 1).^19^ It was thought that upon photoexcitation of this complex, a SET from the amine to **4a** would occur, resulting in the formation of an α-amino alkyl radical (Scheme 1). This radical could rapidly react with a suitable reaction partner. With a suitable oxidant present, the enol radical formed could be regenerated to **4a**, closing the catalytic cycle. Due to the nucleophilic nature of α-amino alkyl radicals, the proposed methodology could be utilized for a broad scope of electrophiles as reaction partners.

As a starting point for the study, N-substituted maleimides were chosen as reaction partners due to their well-known reactivity with aromatic α-amino alkyl radicals, forming tetrahydroquinolines under a range of reaction conditions.^7,20−46^ The photochemically driven annulation reaction between DMA **1a** and *N*-phenyl maleimide (**2a**) to furnish tetrahydroquinoline (**3a**) proceeded smoothly without an external photocatalyst under UV irradiation.^7^ However, the initial screening of reaction conditions, presented in Table 1, shows that visible light irradiation (with a white compact fluorescent lamp) in the absence of a catalyst resulted in a low yield. This slow background reactivity prompted us to use this reaction as a model for our system. Notably, the addition of only 1 mol % dibenzoyl ethylene (**4a**) resulted in a significantly increased yield (Table 1, entry 2), supporting the feasibility of the outlined approach.

**Table 1:**
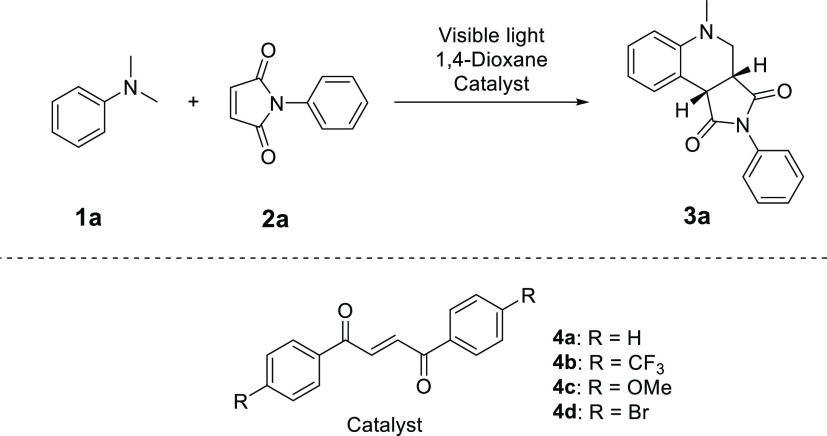
Optimization of the Reaction Conditions

entry^a^	catalyst (mol %)	light source	eq. **1a**	yield **3a**^b^
1		CFL	7	7^c^
2	**4a** (1)	CFL	7	53
3	**4a** (5)	CFL	7	80
4	**4b** (5)	CFL	7	26
5	**4c** (5)	CFL	7	80
6	**4d** (5)	CFL	7	26
7	**4a** (5)	CFL	1	40
8	**4a** (5)	CFL	2	42
9	**4a** (5)	CFL	5	82
10	**4a** (5)		7	12^d^
11	**4a** (5)	green LED	7	50^e^
12	**4a** (5)	blue LED	7	85^f^ (80)^f^^,^^g^
13	**4a** (5)	blue LED	7	3^f^^,^^h^

a Conditions: **2a** (0.2 mmol), **1a** (1–7 equiv), and the catalyst in 3 mL of 1,4-dioxane were irradiated for 6 h under an ambient atmosphere.

b Determined by GC-FID using *n*-dodecane as an internal standard.

c Reaction performed in the absence of the catalyst.

d Reaction performed with protection from light at 100 °C.

e Reaction time, 30 h.

f Reaction time, 7 h.

g Isolated yield.

h Reaction carried out under an atmosphere of nitrogen.

A higher loading of 5 mol % increased the yield further (Table 1, entry 3). The impact of aryl substitutions on the dibenzoyl ethylene catalyst, as a way to tune the catalytic properties, was briefly investigated (Table 1, entries 4–6). Electron-withdrawing groups in the *p*-position resulted in lower yields, while the introduction of an electron-donating group did not affect the catalytic action significantly. Initially, using the amine in excess (7 equiv), we investigated the influence of decreasing amounts and found that with 5 equiv of amine, the reaction proceeded smoothly to yield the desired product at 82% yield (Table 1, entry 9). In order to investigate the effect of the excitation wavelength on the reaction outcome, green and blue light-emitting diodes (LEDs) were used as light sources (Table 1, entries 11 and 12). Green light (525 nm) could promote the transformation with 50% yield using longer irradiation times. A blue LED (465 nm), on the other hand, proved to be a suitable light source, driving the reaction yield to 85%. This result reflects the stronger absorption of the EDA complex in the blue region of the spectrum.

**Scheme 2:**
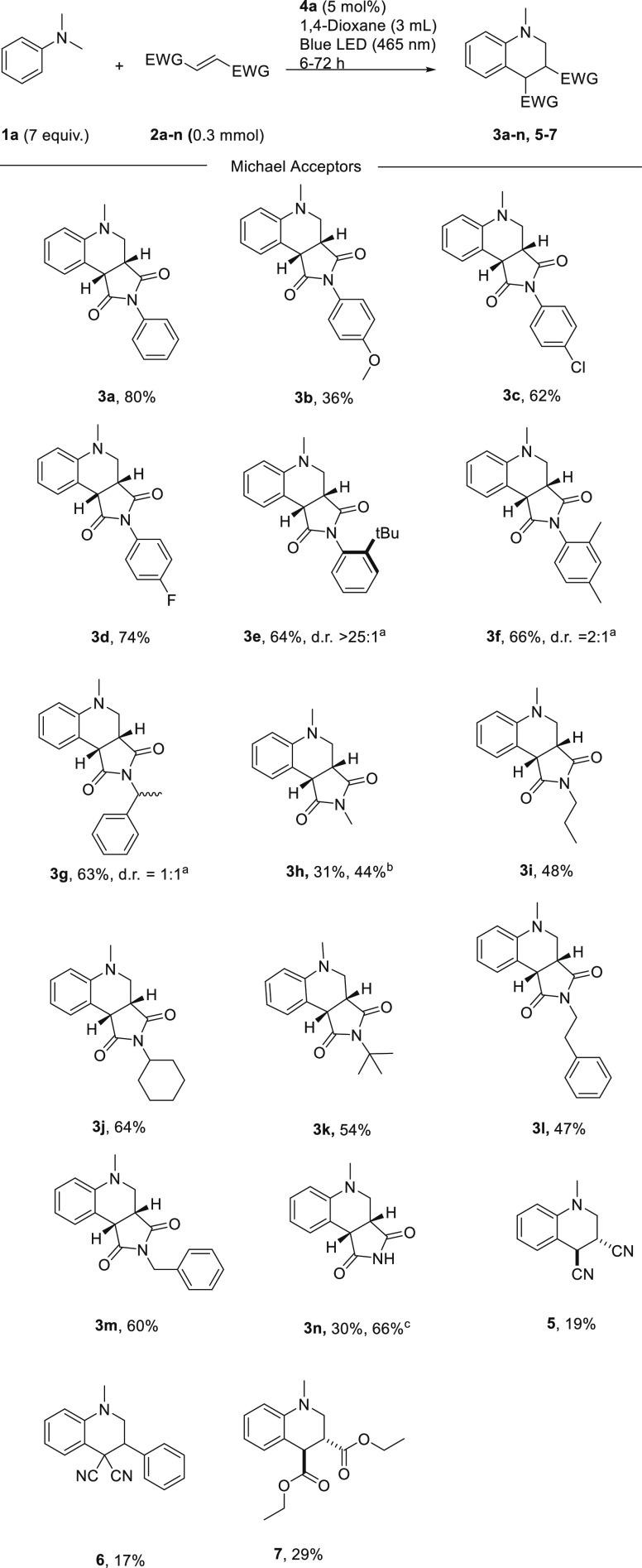
Scope of Activated Alkenes Conditions: alkene (0.3 mmol), amine (7 equiv), and **4a** (5 mol %) in 3 mL of 1,4-dioxane were irradiated for 6–72 h under an ambient atmosphere. Yields reported are isolated. ^a^ Diastereomeric ratio was determined by ^1^H NMR. ^b^ Reaction time, 18 h. ^c^ Reaction time, 72 h.

**Scheme 3:**
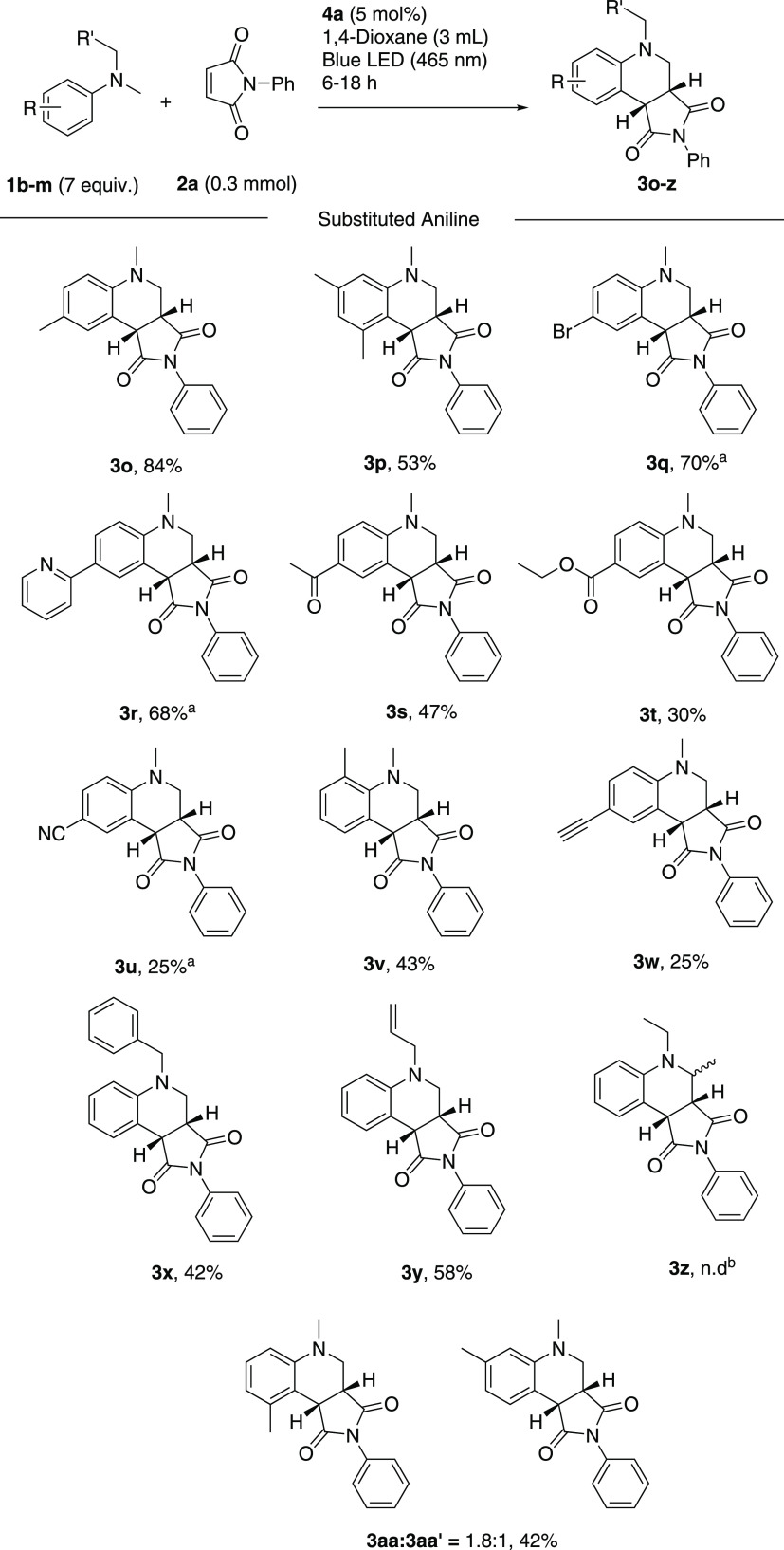
Scope of Amines Conditions: alkene (0.3 mmol), amine (7 equiv), and **4a** (5 mol %) in 3 mL of 1,4-dioxane were irradiated for 6–18 h under an ambient atmosphere. Yields reported are isolated. ^a^ Reaction time 18 h. ^b^ No product was observed in the crude reaction mixture by ^1^H NMR analysis.

With our optimized reaction conditions being found, the substrate scope of the reaction was then investigated (Schemes 2 and 3). Due to the known EDA complexes between anilines and maleimides that potentially could drive the reactions to the desired product in the absence of any catalyst, a control experiment without catalyst **4a** was performed for each entry (Supporting Information, Scheme S1).

First, DMA and different *N*-aryl-substituted maleimides were subjected to the reaction conditions to give products **3b–3f** (Scheme 2). Electron-rich *p*-OMe-substituted maleimide gave product **3b** in a low yield, whereas the slightly electron-withdrawing groups chloro and fluoro provided the desired products **3c** and **3d** in higher yields of 62 and 74%, respectively. The sterically demanding o-substituted *tert*-butyl group was tolerated in the reaction, giving product **3e** in 64% yield as a single diastereomer. On the other hand, compound **3f** bearing the smaller methyl group was obtained as a mixture of diastereomers (2:1 dr) in 66% yield. Next, a set of *N*-alkyl substituted maleimides were tested as Michael acceptors in the radical addition reaction. *N*-Methyl maleimide provided a significantly slower reaction rate with a yield of 31% after 7 h and 44% yield after 18 h. More bulky substituents, such as propyl and *tert*-butyl, gave slightly increased yields of 48 and 54% for **3i** and **3k**, respectively. Non-substituted maleimide provided the desired product **3n** in 30% yield; however, longer reaction times promoted the reaction to proceed to give 66% yield. Michael acceptors other than maleimides resulted in sluggish reactions under the current protocol, giving products **5**–**7** in 17–29% yield.

The effect of substitution on the aniline reaction partner was then explored, giving products **3o–aa** in 25–84% yield (Scheme 3). The reaction was found to be highly sensitive to the electronic nature of the donor amine. Electron-deficient amines, such as *p*-aceto, *p*-carboxylate, and *p*-CN, resulted in lower reaction rates, giving products **3s–u** in 47, 30, and 25% yields, respectively, after 7 h of reaction time. This is presumably due to the higher oxidation potential of these amines.^47^ Complete selectivity toward addition of methyl radicals to the Michael acceptors was observed when different *N*-Me-*N*-alkyl anilines were subjected to the reaction conditions, providing compounds **3x** and **3y**, consistent with the literature.^20,48^ The sterically hindered *o*-methyl-DMA also resulted in lower reactivity (Scheme 3, entry **3v**). When *N*,*N*-diethyl aniline was subjected to the reaction conditions, no annulation product could be observed (Scheme 3, entry **3z**). When *N*,*N*,3-trimethylaniline was used as a starting material, the corresponding tetrahydroquinoline **3aa**/**3aa′** could be isolated as a mixture of regioisomers in (1.8:1) 42% yield.

**Scheme 4:**
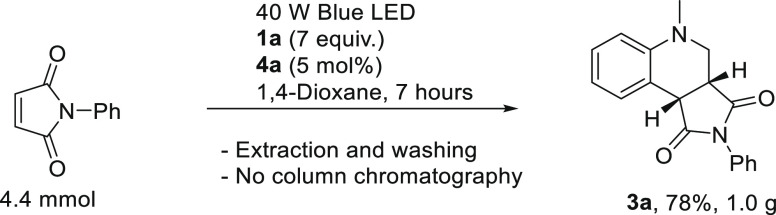
Gram-Scale Synthesis of **3a**

To showcase the scalability of the developed protocol, a gram-scale reaction was carried out (Scheme 4). The desired product **3a** could efficiently be isolated in 78% yield after simple extraction and washing steps.

To gain an insight into the reaction mechanism, several control experiments were carried out. First, when the reaction was carried out in the absence of light at 100 °C for 7 h (Table 1, entry 10), a yield of 12% was obtained. This result shows that although a thermal background reaction can be active at elevated temperatures, light irradiation is needed to successfully drive the reaction. From the background reaction without **4a** as an additive being investigated for almost all substrates (Supporting Information, Scheme S1), it is evident that the catalyst is crucial for promoting the reaction using blue light. Although amines and maleimides formed EDA complexes with absorption that tailed into the visible region (Figure 1), the excitation of these complexes with visible light alone did not seem to result in the efficient formation of reactive radical species. Even with an LED with increased power (40 W) and an emission maximum of 440 nm, where the complex between **1a** and **2a** absorbed significantly, the background reaction proceeded slowly (Supporting Information, Figure S3).

**Scheme 5:**
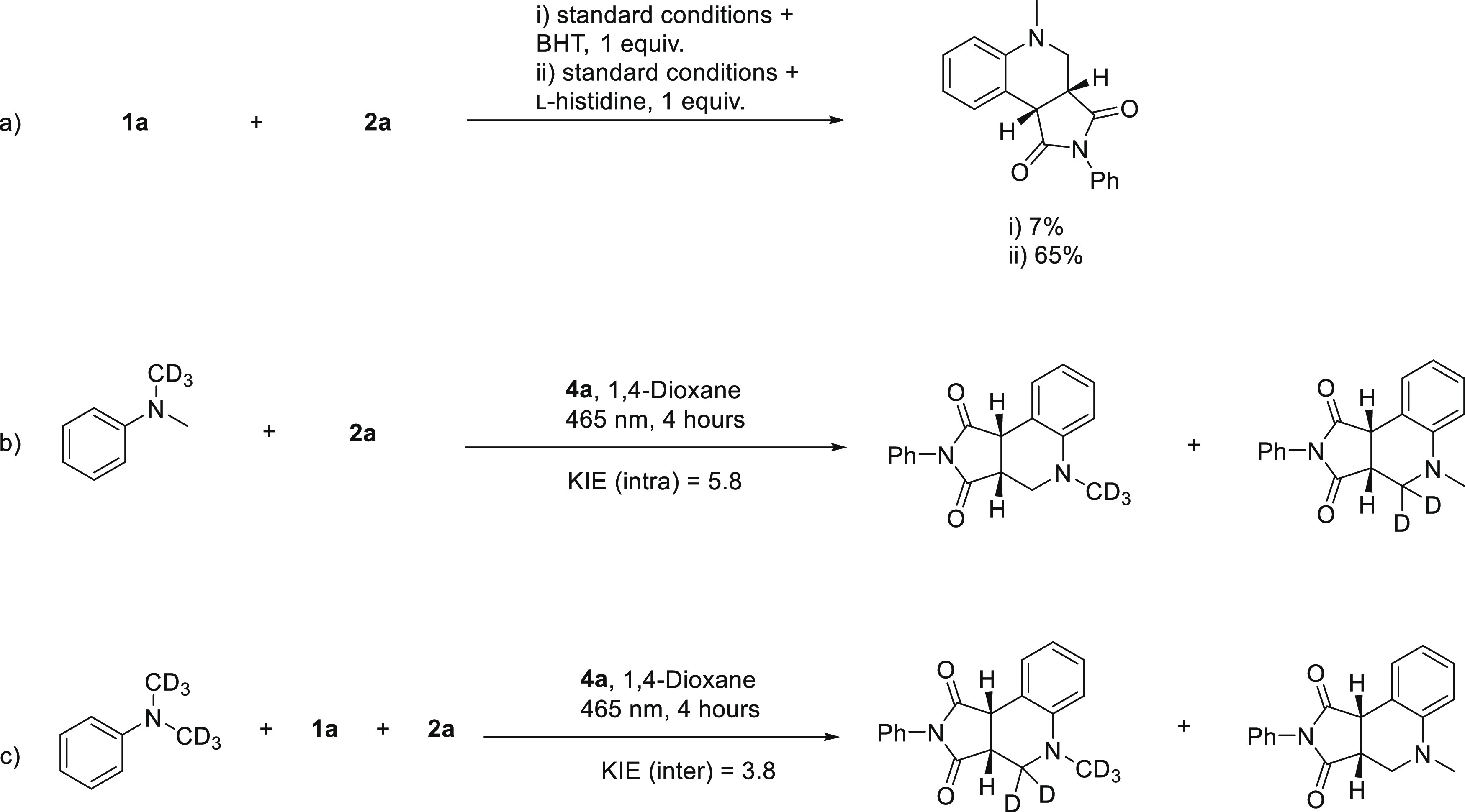
Control Experiments

When the reaction was carried out in the presence of the radical scavenger butylated hydroxytoluene, a significant decrease in the yield was observed, suggesting that the reaction proceeded *via* a radical pathway (Scheme 5a). To investigate if the reaction involved a singlet oxygen species,^49^ a singlet oxygen scavenger, histidine, was investigated, and it was found to have a little impact on the reaction outcome (Scheme 5a).^43^

**Figure 2:**
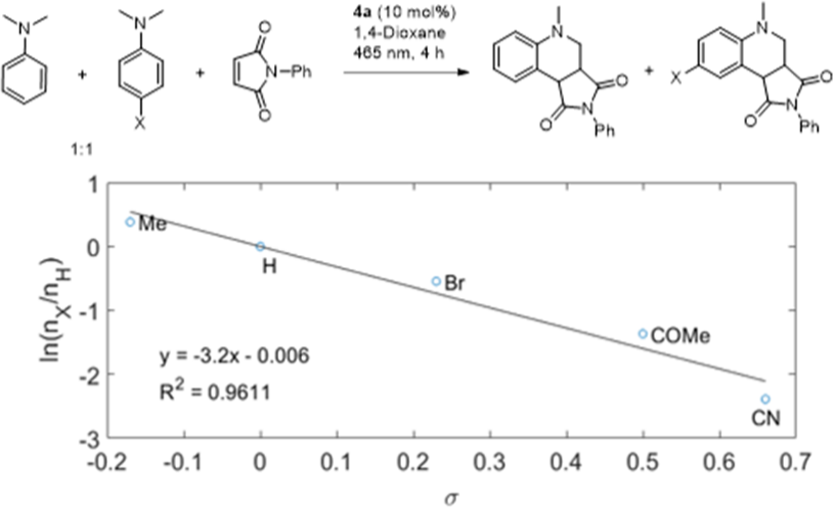
Competition experiment between para-substituted DMA and the plot of the natural logarithms of the relative product ratios, as determined by ^1^H NMR analysis, versus the Hammett parameter σ_p_.

Next, kinetic isotope effect (KIE) experiments were carried out (Scheme 5b,c). The intramolecular KIE was found to be 5.8 (Scheme 5b), whereas an intermolecular KIE of 3.8 was observed when running two parallel reactions with DMA-*d*_6_ and DMA or as a competition experiment (Scheme 5c). The observed difference suggests that the mechanism would not involve a single-step C–H cleavage, such as hydrogen atom transfer, to a significant degree.^50,51^ Instead, the formation of an α-amino alkyl radical from **3a** likely involves two consecutive steps: oxidation and C–H bond cleavage through proton transfer. The oxidation step is an equilibrium between electron transfer and back electron transfer (BET), common to many EDA complexes.^1,2^ In such a system, the intramolecular KIE will be higher than the intermolecular KIE with a difference determined by the ratio between the rate of C–H cleavage and the rate of BET.^50,52^ To investigate the electronic effects of the amine donor, a series of competition experiments were carried out (Figure 2). A significant dependence of the natural logarithms of the relative rates on the Hammett parameter σ_p_ was observed with a slope of −3.2, suggesting that a SET is involved to a significant degree in the mechanism.^51,53^

To investigate the role of air in the reaction, a control experiment under a nitrogen atmosphere was performed which resulted in a product yield of 3% (Table 1, entry 13). The consumption of oxygen was then measured as a function of product formation, and it was observed that a slight excess of oxygen was needed to drive the reaction to completion (Supporting Information, Figure S6).

The quantum yield of the reaction at 450 nm was investigated using standard potassium ferrioxalate actinometry (Supporting Information), and it was found to be 0.07. The low quantum yield can be attributed to the large concentration and absorption by other species such as maleimide and the EDA complex formed between DMA and maleimide, leading to non-productive absorption of photons. Furthermore, given the difference in intra- and intermolecular KIE for the present system, a significant rate of BET can be expected, leading to unproductive absorption of photons.

**Scheme 6:**
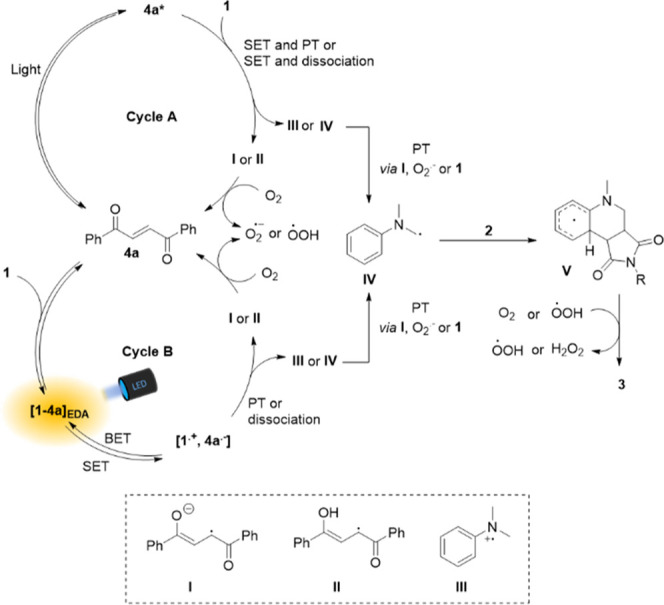
Proposed Mechanism

Based on the control experiments, a possible reaction mechanism can be postulated. The crucial role of **4a** in the reaction outcome can be rationalized according to two likely scenarios. In the first scenario (Scheme 6, cycle A), **4a** is locally excited and promoted to its excited state 4a*, which can oxidize amine **1***via* SET to form **III**, in line with a typical photo-redox catalytic cycle. In the second scenario, however, the key photoactive species is the ground-state EDA complex between **4a** and amine **1** (Scheme 6, cycle B). Excitation of this complex results in the formation of radical anion **I** and the radical cation of the amine *via* a SET within the complex. Subsequently, **I**, or its protonated form **II**, can be oxidized by ground-state molecular oxygen^54^ (rather than by a singlet oxygen species, see Scheme 5a) to yield a superoxide anion or a hydroperoxyl radical.^55^ The deprotonation of the amine radical cation **III** with a p*K*_a_ of 9^56^ can be achieved by proton transfer to either **1**, the radical anion **I**, or a superoxide radical, and yields an α-amino alkyl radical (**IV**) that readily reacts with a Michael acceptor to form **V**, which after aromatization forms the final product **3**. The regenerated **4a** can associate with an amine, forming another EDA complex to close the catalytic cycle.

To completely distinguish between the two scenarios under the developed conditions is, however, challenging. Due to the overlap in absorption of **4a** and its EDA complex with amines (Figure 1), it cannot be ruled out that **4a** is locally excited and acts as a sensitizer. However, the observation that the reaction proceeds with excitation by green light (525 nm) suggests that the EDA complex between **4a** and the amine is a key intermediate in the reaction (Table 1, entry 11). At this wavelength, **4a** has a very low absorption; however, once the EDA complex between **1a** and **4a** has formed, the absorbance is significantly increased. The observation that maleimide reacts with the formed α-amino alkyl radical much faster than the available Michael acceptor **4a** can be rationalized in terms of electrophilicity and the stoichiometry of the reaction conditions. In the absence of light irradiation, a slow background reaction was observed at elevated temperature (Table 1, entry 10). The mechanism for this reaction was not further investigated, although it could be possible that the EDA complex between **1** and **4** or **2** could be activated thermally to some degree, as has been reported for other systems.^57,58^

In conclusion, a protocol for the visible light-induced aerobic oxidative annulation reaction between dialkylanilines and activated alkenes has been disclosed. The reaction is postulated to proceed *via* the excitation of an EDA complex formed between the catalyst 1,2-dibenzoyl ethylene and the amine reaction partner. The simple and available 1,2-dibenzoyl ethylene makes the developed protocol attractive as an alternative to the use of complex photoredox catalysts. Furthermore, this example of a catalytic external acceptor could stimulate the field of EDA complex chemistry to pursue novel photoreactions.

## Experimental Section

### General Information

All reagents and solvents were purchased from Sigma-Aldrich and Alfa Aesar and used without any further purification unless specified. Purification was performed in an automated column chromatograph Biotage Isolera Spektra One with a Biotage SNAP-10 g KP-silica column, together with a 1 g Samplet cartridge using *n*-heptane or petroleum ether (40–60 °C)/ethyl acetate as solvent mixture unless otherwise noted. ^1^H (400 MHz) and ^13^C (101 MHz) NMR spectra were acquired on an Agilent NMR machine at 25 °C. The chemical shifts for ^1^H and ^13^C NMR spectra are reported in parts per million (ppm) relative to the residual peak from the solvent CDCl_3_ as the internal standard: ^1^H NMR at δ 7.26 ppm and ^13^C NMR at δ 77.16 ppm for CDCl_3_. All coupling constants (*J*) are reported in hertz, and multiplicities are indicated by s (singlet), d (doublet), dd (doublet of doublet), td (triplet of doublet), ddd (doublet of doublets of doublets), (t) triplett, dt (doublet of triplet), and m (multiplet). Infrared (IR) spectra were recorded on a PerkinElmer series ATR FTIR spectrometer and are reported in wavenumber (cm^–1^).

High-resolution mass spectrometry measurements were performed by CMSI service at Chalmers University of Technology. An Agilent 6520 equipped with an electrospray interface was operated in the positive ionization mode.

UV–vis absorption spectra were recorded on a Cary 4000 UV–vis spectrometer using 1 × 1 cm quartz cuvettes. All light-promoted reactions were carried out in Biotage microwave vials (2–5 mL) under irradiation with a commercial LED strip (Ledsavers, 5 W, λ_max_ 465 nm), a commercial compact fluorescent lamp (Narva Scandinavia, UV light bulb, 15 W, λ_max_ 365 nm), a Kessil PR160L-440 LED lamp, or a Kessil PR160L-525 LED lamp. Gas chromatography studies were performed using an Agilent 7820A equipped with a flame ionization detector and an Agilent HP-5 19091J-413 column. Emission spectra of light sources were measured using an AvaSpec-2048.

### Synthesis of Starting Materials

Maleimides **2b**, **2d**, **2e**, **2f**, and **2l** were synthesized following a modified reported method.^59^ The appropriate amine (10 mmol) dissolved in diethyl ether (5 mL) was added to a stirred solution of maleic anhydride (10 mmol, 1 g) in diethyl ether (10 mL). The solution was stirred at room temperature for 4–18 h, and the resulting suspension was filtered. The collected solids were washed with diethyl ether and then dried *in vacuo* and directly added to acetic anhydride (6 mL), followed by sodium acetate (1 equiv). The suspension was heated to 120 °C until full conversion as followed by TLC (1–4 h reaction time). The solvent was then removed under reduced pressure, and the residue was taken up with ethyl acetate and washed with saturated sodium bicarbonate solution and brine. The organic phase was then dried over anhydrous sodium sulfate and was concentrated under reduced pressure. The solid residue was recrystallized from ethanol or ethyl acetate to afford the desired maleimide as a crystalline solid or purified by column chromatography for oils.

#### 1-(4-Methoxyphenyl)-1*H*-pyrrole-2,5-dione (**2b**)^60^

A yellow solid afforded after recrystallization from ethanol, 1.2 g (58%); spectroscopic data were in accordance with the literature;^60^^1^H NMR (400 MHz, chloroform-*d*): δ 7.25–7.19 (m, 2H), 7.02–6.94 (m, 2H), 6.84 (s, 2H), 3.83 (s, 3H) ppm; ^13^C{^1^H} NMR (101 MHz, chloroform-*d*): δ 169.9, 159.3, 134.2, 127.7, 123.8, 114.6, 55.6 ppm.

#### 1-(4-Fluorophenyl)-1*H*-pyrrole-2,5-dione (**2d**)^61^

A yellow crystalline solid obtained after recrystallization from ethyl acetate/heptane, 937 mg (50%); spectroscopic data were in accordance with the literature;^61^^1^H NMR (400 MHz, chloroform-*d*): δ 7.38–7.28 (m, 2H), 7.21–7.12 (m, 2H), 6.86 (d, *J* = 0.8 Hz, 2H); ^13^C{^1^H} NMR (101 MHz, chloroform-*d*): δ 169.5, 162.0 (d, *J* = 248 Hz), 134.4, 128.0 (d, *J* = 8.7 Hz), 127.2 (d, *J* = 3.3 Hz), 116.3 (d, *J* = 22.9 Hz) ppm.

#### 1-(2-(*tert*-Butyl)phenyl)-1*H*-pyrrole-2,5-dione (**2e**)^62^

An off-white solid obtained after recrystallization thrice from ethanol (500 mg, 36%). Spectroscopic data were in accordance with the literature;^62^^1^H NMR (400 MHz, chloroform-*d*): δ 7.59 (dd, *J* = 8.2, 1.5 Hz, 1H), 7.41 (tdd, *J* = 7.4, 1.6, 0.8 Hz, 1H), 7.31–7.27 (m, 1H), 6.93–6.87 (m, 3H), 1.30 (s, 9H) ppm; ^13^C{^1^H} NMR (101 MHz, chloroform-*d*): δ 171.0, 149.7, 135.1, 131.5, 130.0, 129.3, 128.8, 127.5, 35.6, 31.7 ppm.

#### 1-(2,4-Dimethylphenyl)-1*H*-pyrrole-2,5-dione (**2f**)^63^

A yellow oil obtained after column chromatography (silica, 10% ethyl acetate in petroleum spirit), 1.47 g (34%); spectroscopic data were in accordance with the literature;^63^^1^H NMR (400 MHz, chloroform-*d*): δ 7.14 (dq, *J* = 2.0, 0.7 Hz, 1H), 7.13–7.08 (m, 1H), 6.99 (d, *J* = 8.0 Hz, 1H), 6.86 (s, 2H), 2.35 (s, 3H), 2.11 (s, 3H) ppm; ^13^C{^1^H} NMR (101 MHz, chloroform-*d*): δ 167.0, 139.7, 136.3, 134.5, 132.0, 128.6, 127.8, 127.3, 21.3, 17.9 ppm.

#### 1-Phenethyl-1*H*-pyrrole-2,5-dione (**2l**)^64^

Pink flakes isolated after recrystallization from ethanol twice, 894 mg (44%); spectroscopic data were in accordance with the literature;^64^^1^H NMR (400 MHz, chloroform-*d*): δ 7.33–7.26 (m, 2H), 7.25–7.17 (m, 3H), 6.65 (s, 2H), 3.82–3.72 (m, 2H), 2.95–2.83 (m, 2H) ppm; ^13^C{^1^H} NMR (101 MHz, chloroform-*d*): δ 170.7, 138.0, 134.2, 129.0, 128.7, 126.8, 39.3, 34.7 ppm.

### General Procedure for the Visible-Light-Driven Aerobic Oxidative Annulation Reaction

To a 2–5 mL Biotage microwave vial were added N-substituted maleimide (0.3 mmol, 1 equiv), the appropriate amine (1.75 mmol, 7 equiv), and 1,2-dibenzoyl ethylene (0.013 mmol, 0.05 equiv). 1,4-Dioxane (3 mL) was then added, and the solution was irradiated using a 465 nm LED stripe with a distance of 2 cm to the irradiation source for 6–18 h, after which the solvent was removed under reduced pressure and the crude residue was loaded on a silica column and eluted with a mixture of ethyl acetate in petroleum ethers to afford the desired tetrahydroquinoline products **3a–aa** and **5–7**.

#### 5-Methyl-2-phenyl-3a,4,5,9b-tetrahydro-1*H*-pyrrolo[3,4-*c*]quinoline-1,3(2*H*)-dione (**3a**)^7^

Starting with 43 mg of *N*-phenyl maleimide following the general procedure, **3a** was obtained as a yellow solid (58.3 mg, 80%) after purification using column chromatography (SiO_2_, 0–10% ethyl acetate in petroleum ether). Spectroscopic data were in accordance with the literature;^7^^1^H NMR (400 MHz, chloroform-*d*): δ 7.54 (dd, *J* = 7.6, 1.4 Hz, 1H), 7.47–7.40 (m, 2H), 7.39–7.32 (m, 1H), 7.29–7.21 (m, 3H), 6.92 (tt, *J* = 7.5, 1.2 Hz, 1H), 6.76 (d, *J* = 8.2 Hz, 1H), 4.17 (d, *J* = 9.6 Hz, 1H), 3.66–3.59 (m, 1H), 3.59–3.51 (m, 1H), 3.14 (ddd, *J* = 11.4, 4.4, 1.0 Hz, 1H), 2.85 (s, 3H) ppm; ^13^C{^1^H} NMR (101 MHz, chloroform-*d*): δ 177.8, 175.9, 148.5, 132.1, 130.5, 129.1, 128.8, 128.6, 126.5, 119.9, 118.7, 112.8, 50.8, 43.7, 42.3, 39.6 ppm.

For the gram-scale synthesis version of **3a**, to a 100 mL round-bottom flask were added *N*-phenylmaleimide (762 mg, 4.4 mmol, 1 equiv), 1,2-dibenzoyl ethylene (52 mg, 0.22 mmol, 0.05 equiv), DMA (3.7 g, 30.5 mmol, 6.9 equiv), and 1,4-dioxane (50 mL). The reaction mixture was then placed 5 cm from a 40 W blue LED lamp and irradiated for 7 h under vigorous stirring. The round-bottom flask was kept open during the reaction to facilitate easy diffusion of oxygen to drive the reaction. After full conversion, as judged by TLC (20% ethyl acetate in *n*-heptane), the solvent was removed under reduced pressure and the residue was taken up in ethyl acetate. Excess aniline was removed by washing with HCl (3 × 10 mL, 1 M). The organic layer was then washed with brine and was dried over sodium sulfate. The solvent was then removed under reduced pressure to yield a brown solid that was suspended in ethyl acetate/heptane and was collected by filtration. The solids were washed with a small amount of ethanol and then excessive amounts of *n*-heptane. After drying *in vacuo*, **3a** was obtained as an off-white powder (1.0 g, 3.4 mmol, 78%).

#### 2-(4-Methoxyphenyl)-5-methyl-3a,4,5,9b-tetrahydro-1*H*-pyrrolo[3,4-*c*]quinoline-1,3(2*H*)-dione (**3b**)^35^

Starting with 49.9 mg of *N*-(4-methoxyphenyl)maleimide following the general procedure provided **3b** as a yellow solid (27.4 mg, 36%) after purification using column chromatography (SiO_2_, 0–10% ethyl acetate in petroleum ether). Spectroscopic data were in accordance with the literature;^35^^1^H NMR (400 MHz, chloroform-*d*): δ 7.53 (ddd, *J* = 7.5, 1.6, 0.8 Hz, 1H), 7.27–7.21 (m, 1H), 7.21–7.14 (m, 2H), 6.98–6.90 (m, 3H), 6.75 (dd, *J* = 8.2, 1.1 Hz, 1H), 4.14 (d, *J* = 9.5 Hz, 1H), 3.80 (s, 3H), 3.60 (dd, *J* = 11.4, 2.8 Hz, 1H), 3.52 (ddd, *J* = 9.6, 4.4, 2.7 Hz, 1H), 3.12 (dd, *J* = 11.5, 4.4 Hz, 1H), 2.84 (s, 3H) ppm; ^13^C{^1^H} NMR (101 MHz, chloroform-*d*): δ 178.1, 176.1, 159.5, 148.6, 130.5, 128.8, 127.7, 124.7, 119.8, 118.8, 114.4, 112.7, 55.6, 50.8, 43.6, 42.2, 39.6 ppm.

#### 2-(4-Chlorophenyl)-5-methyl-3a,4,5,9b-tetrahydro-1*H*-pyrrolo[3,4-*c*]quinoline-1,3(2*H*)-dione (**3c**)^7^

Starting with 52.6 mg of *N*-(4-chlorophenyl)maleimide following the general procedure provided a white solid (51 mg, 62%) after purification using column chromatography (SiO_2_, 0–10% ethyl acetate in petroleum ether). Spectroscopic data were in accordance with the literature;^7^^1^H NMR (400 MHz, chloroform-*d*): δ 7.51 (ddd, *J* = 7.5, 1.7, 0.8 Hz, 1H), 7.45–7.34 (m, 2H), 7.29–7.20 (m, 4H), 6.92 (td, *J* = 7.5, 1.1 Hz, 1H), 6.75 (dd, *J* = 8.3, 1.1 Hz, 1H), 4.15 (d, *J* = 9.6 Hz, 1H), 3.60 (dd, *J* = 11.5, 2.7 Hz, 1H), 3.52 (ddd, *J* = 9.6, 4.4, 2.7 Hz, 1H), 3.11 (dd, *J* = 11.5, 4.4 Hz, 1H), 2.84 (s, 3H) ppm; ^13^C{^1^H} NMR (101 MHz, chloroform-*d*): δ 177.5, 175.6, 148.6, 134.3, 130.5, 130.4, 129.3, 128.9, 127.7, 119.9, 118.5, 112.7, 50.7, 43.7, 42.2, 39.6 ppm.

#### 2-(4-Fluorophenyl)-5-methyl-3a,4,5,9b-tetrahydro-1*H*-pyrrolo[3,4-*c*]quinoline-1,3(2*H*)-dione (**3d**)^22^

Starting with 48.3 mg of *N*-(4-fluorophenyl)maleimide following the general procedure provided a white solid (57.5 mg, 74%) after purification using column chromatography (SiO_2_, 0–10% ethyl acetate in petroleum ether). Spectroscopic data were in accordance with the literature;^22^^1^H NMR (400 MHz, chloroform-*d*): δ 7.52 (ddd, *J* = 7.6, 1.6, 0.8 Hz, 1H), 7.29–7.20 (m, 3H), 7.15–7.07 (m, 2H), 6.92 (td, *J* = 7.5, 1.2 Hz, 1H), 6.75 (dd, *J* = 8.3, 1.1 Hz, 1H), 4.15 (d, *J* = 9.6 Hz, 1H), 3.60 (dd, *J* = 11.5, 2.7 Hz, 1H), 3.52 (ddd, *J* = 9.6, 4.4, 2.6 Hz, 1H), 3.11 (dd, *J* = 11.5, 4.4 Hz, 1H), 2.84 (s, 3H); ^13^C{^1^H} NMR (101 MHz, chloroform-*d*): δ 177.8, 175.8, 162.2 (d, ^1^*J*_C–F_ = 248.4 Hz), 148.6, 130.4, 128.8, 128.3 (d, ^3^*J*_C–F_ = 8.7 Hz), 128.0 (d, ^4^*J*_C–F_ = 3.3 Hz), 119.8, 118.5, 116.1 (d, ^2^*J*_C–F_ = 22.9 Hz), 112.7, 50.7, 43.7, 42.2, 39.5 ppm.

#### 2-(2-(*tert*-Butyl)phenyl)-5-methyl-3a,4,5,9b-tetrahydro-1*H*-pyrrolo[3,4-*c*]quinoline-1,3(2*H*)-dione (**3e**)

Starting with 58.9 mg of *N*-(2-*tert*butylphenyl)maleimide following the general procedure with an irradiation time of 18 h provided **3e** as an orange oil (56.9 mg, 64%) after purification using column chromatography (SiO_2_, 0–10% ethyl acetate in petroleum ether). ^1^H NMR (400 MHz, chloroform-*d*): δ 7.54 (dddd, *J* = 22.0, 7.5, 1.5, 0.7 Hz, 2H), 7.39–7.32 (m, 1H), 7.29–7.23 (m, 1H), 7.22–7.16 (m, 1H), 6.92 (tt, *J* = 7.5, 0.9 Hz, 1H), 6.78 (dd, *J* = 8.2, 1.1 Hz, 1H), 6.68 (dd, *J* = 7.8, 1.5 Hz, 1H), 4.16 (d, *J* = 9.6 Hz, 1H), 3.61 (ddd, *J* = 11.4, 2.8, 0.7 Hz, 1H), 3.58–3.48 (m, 1H), 3.11 (ddd, *J* = 11.4, 4.6, 0.8 Hz, 1H), 2.86 (d, *J* = 0.7 Hz, 3H), 1.36 (t, *J* = 0.6 Hz, 9H) ppm; ^13^C{^1^H} NMR (101 MHz, chloroform-*d*): δ 179.1, 176.9, 162.5, 148.9, 147.8, 131.0, 130.6, 129.8, 128.8, 128.7, 127.4, 119.9, 119.1, 112.6, 51.1, 44.0, 42.6, 39.6, 35.8, 31.8 ppm; FTIR ν: 2958, 1709, 1495, 1375, 1316, 1195, 1181 cm^–1^; HRMS (ESI) *m*/*z*: calcd C_22_H_24_N_2_O_2_ [M + H]^+^, 349.1916; found, 349.1917.

#### 2-(2-(*tert*-Butyl)phenyl)-5-methyl-3a,4,5,9b-tetrahydro-1*H*-pyrrolo[3,4-*c*]quinoline-1,3(2*H*)-dione (**3e′**)

Diastereomer **3e′** was furnished from **3e** as follows: to a 2–5 mL Biotage microwave vial was added **3e** (20 mg, 0.057 mmol). The vial was then capped and heated to 160 °C using a metal heating block. After 1 h of heating, the vial was cooled to room temperature and the crude mixture was purified using silica flash chromatography (10% ethyl acetate in petroleum ether) to afford **3e** (8.4 mg, 42%) and **3e′** (6.9 mg, 35%) as white solids. mp 195.4–196.7 °C; ^1^H NMR (400 MHz, chloroform-*d*): δ 7.54–7.46 (m, 2H), 7.36 (ddd, *J* = 8.1, 7.3, 1.6 Hz, 1H), 7.29 (dd, *J* = 7.5, 1.5 Hz, 1H), 7.25–7.19 (m, 1H), 6.92–6.85 (m, 2H), 6.73 (dd, *J* = 8.3, 1.1 Hz, 1H), 4.18 (d, *J* = 9.5 Hz, 1H), 3.70 (dd, *J* = 11.5, 2.2 Hz, 1H), 3.54 (ddd, *J* = 9.6, 4.0, 2.2 Hz, 1H), 3.16 (dd, *J* = 11.5, 4.0 Hz, 1H), 2.84 (s, 3H), 1.00 (s, 9H) ppm; ^13^C{^1^H} NMR (101 MHz, chloroform-*d*): δ 179.1, 177.0, 148.8, 148.6, 131.4, 130.9, 130.3, 129.8, 128.8, 128.4, 127.5, 119.8, 118.1, 112.4, 50.4, 45.0, 43.1, 39.1, 35.1, 30.7 ppm; FTIR ν: 2966, 1710, 1497, 1371, 1265, 1180 cm^–1^; HRMS (ESI) *m*/*z*: calcd C_22_H_24_N_2_O_2_ [M + H]^+^, 349.1916; found, 349.1921.

#### 2-(2,4-Dimethylphenyl)-5-methyl-3a,4,5,9b-tetrahydro-1*H*-pyrrolo[3,4-*c*]quinoline-1,3(2*H*)-dione (**3f**)

Starting with 53.0 mg of *N*-(2,4-dimethylphenyl)maleimide following the general procedure provided **3f** as a 2:1 mixture of diastereomers as an orange oil (55.3 mg, 66%) after purification using column chromatography (SiO_2_, 0–10% ethyl acetate in petroleum ether). ^1^H NMR (400 MHz, chloroform-*d*): δ 7.56–7.48 (m, 3H), 7.26–7.22 (m, 3H), 7.15–6.98 (m, 8H), 6.95–6.88 (m, 3H), 6.86–6.72 (m, 4H), 4.23–4.12 (m, 3H), 3.66–3.50 (m, 6H), 3.15–3.06 (m, 3H), 2.86 (s, 3H), 2.85 (s, 6H), 2.35–2.28 (m, 9H), 2.16 (s, 3H), 1.80 (s, 6H) ppm; ^13^C{^1^H} NMR (101 MHz, chloroform-*d*): δ 178.1, 177.9, 176.1, 175.9, 148.7 (2C), 139.6, 139.4, 135.6, 135.0, 131.9 (2C), 130.5, 130.4, 128.7, 128.6, 128.0, 127.6 (3C), 119.8 (2C), 119.2, 118.8, 112.6, 112.4, 51.2, 50.9, 44.4, 43.7, 42.9, 42.4, 39.6, 39.3, 21.3 (2C), 17.8, 17.0 ppm; FTIR ν: 2924, 1706, 1498, 1386, 1197, 1180 cm^–1^; HRMS (ESI) *m*/*z*: calcd C_20_H_20_N_2_O_2_ [M + H]^+^, 321.1603; found, 321.1612.

#### 5-Methyl-2-(1-phenylethyl)-3a,4,5,9b-tetrahydro-1*H*-pyrrolo[3,4-*c*]quinoline-1,3(2*H*)-dione (**3g**)^65^

Starting with 54.4 mg of 1-(1-phenylethyl)-1*H*-pyrrole-2,5-dione following the general procedure provided **3g** as a 1:1 mixture of diastereomers as a brown oil (54.6 mg, 63%) after purification using column chromatography (SiO_2_, 0–10% ethyl acetate in petroleum ether). Spectroscopic data were in accordance with the literature.^65^^1^H NMR (400 MHz, chloroform-*d*): δ 7.48–7.43 (m, 2H), 7.35 (ddtd, *J* = 6.1, 4.6, 1.6, 0.7 Hz, 4H), 7.32–7.18 (m, 8H), 6.89 (dtd, *J* = 8.7, 7.5, 1.2 Hz, 2H), 6.72 (ddd, *J* = 8.2, 2.2, 1.1 Hz, 2H), 5.47–5.31 (m, 2H), 3.98–3.87 (m, 2H), 3.44 (ddd, *J* = 11.4, 9.2, 3.0 Hz, 2H), 3.37–3.24 (m, 2H), 3.04 (ddd, *J* = 11.3, 6.4, 4.6 Hz, 2H), 2.82 (s, 3H), 2.78 (s, 3H), 1.79 (d, *J* = 7.3 Hz, 3H), 1.75 (d, *J* = 7.3 Hz, 3H) ppm; ^13^C{^1^H} NMR (101 MHz, chloroform-*d*): δ 178.6, 178.5, 176.7, 176.5, 148.6 (2C), 139.5 (2C), 130.3 (2C), 128.6 (2C), 128.5, 128.4, 127.7 (2C), 127.2 (2C), 119.8, 119.7, 119.3, 119.0, 112.5 (2C), 51.2, 51.0, 50.7, 50.6, 43.5, 43.4, 42.1, 42.0, 39.5, 39.4, 16.9, 16.8 ppm.

#### 2,5-Dimethyl-3a,4,5,9b-tetrahydro-1*H*-pyrrolo[3,4-*c*]quinoline-1,3(2*H*)-dione (**3h**)^35^

Starting from 27.8 mg of *N*-methylmaleimide following the general procedure, **3h** as a white solid (18.0 mg, 31% with a 6 h reaction time or 26 mg, 44% with an 18 h reaction time) was isolated after purification using column chromatography (SiO_2_, 0–10% ethyl acetate in petroleum ether); spectroscopic data were in accordance with the literature;^35^^1^H NMR (400 MHz, chloroform-*d*): δ 7.47 (ddd, *J* = 7.4, 1.7, 0.8 Hz, 1H), 7.24–7.14 (m, 1H), 6.89 (td, *J* = 7.5, 1.1 Hz, 1H), 6.69 (dd, *J* = 8.3, 1.1 Hz, 1H), 3.99 (d, *J* = 9.4 Hz, 1H), 3.53 (dd, *J* = 11.5, 2.5 Hz, 1H), 3.36 (ddd, *J* = 9.4, 4.4, 2.4 Hz, 1H), 3.03 (dd, *J* = 11.5, 4.4 Hz, 1H), 2.98 (s, 3H), 2.79 (s, 3H); ^13^C{^1^H} NMR (101 MHz, chloroform-*d*): δ 178.9, 176.9, 148.5, 130.3, 128.7, 119.8, 118.9, 112.7, 50.6, 43.7, 42.1, 39.6, 25.5 ppm.

#### 5-Methyl-2-propyl-3a,4,5,9b-tetrahydro-1*H*-pyrrolo[3,4-*c*]quinoline-1,3(2*H*)-dione (**3i**)^7^

Starting from 34.1 mg of *N*-propylmaleimide following the general procedure, **3i** as a white solid (30.2 mg, 48%) was isolated after purification using column chromatography (SiO_2_, 0–10% ethyl acetate in petroleum ether). Spectroscopic data were in accordance with the literature;^7^^1^H NMR (400 MHz, chloroform-*d*): δ 7.48–7.42 (m, 1H), 7.22–7.14 (m, 1H), 6.87 (td, *J* = 7.4, 1.2 Hz, 1H), 6.69 (dd, *J* = 8.2, 1.3 Hz, 1H), 3.95 (d, *J* = 9.3 Hz, 1H), 3.53–3.40 (m, 3H), 3.33 (ddd, *J* = 9.0, 4.3, 2.4 Hz, 1H), 3.01 (dd, *J* = 11.5, 4.5 Hz, 1H), 2.78 (s, 3H), 1.66–1.47 (m, 2H), 0.81 (td, *J* = 7.4, 1.7 Hz, 3H) ppm; ^13^C{^1^H} NMR (101 MHz, chloroform-*d*): δ 178.9, 176.9, 148.4, 130.3, 128.6, 119.8, 119.2, 112.6, 50.6, 43.6, 42.1, 41.0, 39.5, 21.0, 11.1 ppm.

#### 2-Cyclohexyl-5-methyl-3a,4,5,9b-tetrahydro-1*H*-pyrrolo[3,4-*c*]quinoline-1,3(2*H*)-dione (**3j**)^7^

Starting from 47.4 mg of *N*-cyclohexylmaleimide following the general procedure, **3j** as a yellow oil (49.9 mg, 64%) was isolated after purification using column chromatography (SiO_2_, 0–10% ethyl acetate in petroleum ether). Spectroscopic data were in accordance with the literature;^7^^1^H NMR (400 MHz, chloroform-*d*): δ 7.46 (ddd, *J* = 7.5, 1.7, 0.7 Hz, 1H), 7.23–7.14 (m, 1H), 6.88 (td, *J* = 7.5, 1.1 Hz, 1H), 6.69 (dd, *J* = 8.2, 1.1 Hz, 1H), 4.00–3.85 (m, 2H), 3.45 (dd, *J* = 11.4, 3.0 Hz, 1H), 3.28 (ddd, *J* = 9.5, 4.6, 3.0 Hz, 1H), 3.02 (dd, *J* = 11.4, 4.6 Hz, 1H), 2.79 (s, 3H), 2.21–1.96 (m, 2H), 1.84–1.72 (m, 2H), 1.66–1.58 (m, 1H), 1.58–1.49 (m, 2H), 1.37–1.09 (m, 3H) ppm; ^13^C{^1^H} NMR (101 MHz, chloroform-*d*): δ 178.8, 176.9, 148.5, 130.3, 128.5, 119.5, 119.1, 112.4, 52.2, 50.9, 43.1, 41.8, 39.5, 28.9, 28.8, 25.9, 25.8, 25.1 ppm.

#### 2-(*tert*-Butyl)-5-methyl-3a,4,5,9b-tetrahydro-1*H*-pyrrolo[3,4-*c*]quinoline-1,3(2*H*)-dione (**3k**)^7^

37.8 mg of *N*-*t*Bu-maleimide provided an orange oil, 37.3 mg (54%), after purification using column chromatography (SiO_2_, 0–10% ethyl acetate in petroleum ether). Spectroscopic data were in accordance with the literature;^7^^1^H NMR (400 MHz, chloroform-*d*): δ 7.45 (dddd, *J* = 7.5, 1.7, 0.9, 0.4 Hz, 1H), 7.25–7.18 (m, 1H), 6.88 (td, *J* = 7.5, 1.2 Hz, 1H), 6.72 (dd, *J* = 8.2, 1.1 Hz, 1H), 3.83 (d, *J* = 9.7 Hz, 1H), 3.44 (dd, *J* = 11.4, 3.1 Hz, 1H), 3.21 (ddd, *J* = 9.7, 4.6, 3.1 Hz, 1H), 3.01 (dd, *J* = 11.4, 4.6 Hz, 1H), 2.81 (s, 3H), 1.54 (s, 9H) ppm; ^13^C{^1^H} NMR (101 MHz, chloroform-*d*): δ 179.6, 177.9, 148.2, 130.4, 128.6, 119.7, 119.4, 112.7, 58.9, 51.0, 43.1, 42.2, 39.7, 28.5 ppm.

#### 5-Methyl-2-phenethyl-3a,4,5,9b-tetrahydro-1*H*-pyrrolo[3,4-*c*]quinoline-1,3(2*H*)-dione (**3l**)

Starting with 52.9 mg of *N*-phenethylmaleimide following the general procedure provided **3l** as an orange oil (39.4 mg, 47%) after purification using column chromatography (SiO_2_, 0–10% ethyl acetate in petroleum ether). ^1^H NMR (400 MHz, chloroform-*d*): δ 7.45 (ddd, *J* = 7.6, 1.6, 0.8 Hz, 1H), 7.25–7.13 (m, 4H), 7.10 (dd, *J* = 7.7, 1.9 Hz, 2H), 6.89 (td, *J* = 7.4, 1.1 Hz, 1H), 6.71 (dd, *J* = 8.3, 1.1 Hz, 1H), 3.93 (d, *J* = 9.4 Hz, 1H), 3.74 (t, *J* = 7.4 Hz, 2H), 3.47 (dd, *J* = 11.5, 2.6 Hz, 1H), 3.28 (ddd, *J* = 9.4, 4.4, 2.6 Hz, 1H), 3.01 (dd, *J* = 11.5, 4.4 Hz, 1H), 2.85 (td, *J* = 7.2, 1.4 Hz, 2H), 2.78 (s, 3H) ppm; ^13^C{^1^H} NMR (101 MHz, chloroform-*d*): δ 178.5, 176.6, 148.4, 137.8, 130.2, 128.9, 128.6, 128.5, 126.6, 119.6, 118.8, 112.5, 50.4, 43.5, 42.0, 40.5, 39.5, 33.5 ppm; ATR-FTIR ν: 2949, 1698, 1499, 1401, 1354, 1159 cm^–1^; HRMS (ESI) *m*/*z*: calcd C_20_H_20_N_2_O_2_ [M + H]^+^, 321.1603; found, 321.1610.

#### 2-Benzyl-5-methyl-3a,4,5,9b-tetrahydro-1*H*-pyrrolo[3,4-*c*]quinoline-1,3(2*H*)-dione (**3m**)^7^

Starting with 46.5 mg of *N*-benzylmaleimide following the general procedure provided a white solid (45.3 mg, 60%) after purification using column chromatography (SiO_2_, 0–10% ethyl acetate in petroleum ether). Spectroscopic data were in accordance with the literature;^7^^1^H NMR (400 MHz, chloroform-*d*): δ 7.51–7.41 (m, 1H), 7.38–7.13 (m, 6H), 6.90 (td, *J* = 7.5, 1.2 Hz, 1H), 6.71 (dd, *J* = 8.2, 1.1 Hz, 1H), 4.76–4.51 (m, 2H), 3.99 (d, *J* = 9.4 Hz, 1H), 3.50 (dd, *J* = 11.5, 2.7 Hz, 1H), 3.36 (ddd, *J* = 9.4, 4.6, 2.8 Hz, 1H), 3.05 (dd, *J* = 11.5, 4.6 Hz, 1H), 2.80 (s, 3H) ppm; ^13^C{^1^H} NMR (101 MHz, chloroform-*d*): δ 178.4, 176.5, 148.5, 135.7, 130.3, 128.7, 128.7, 128.4, 127.9, 119.8, 119.0, 112.6, 50.8, 43.7, 42.9, 42.2, 39.5 ppm.

#### 5-Methyl-3a,4,5,9b-tetrahydro-1*H*-pyrrolo[3,4-*c*]quinoline-1,3(2*H*)-dione (**3n**)^66^

Starting with 25.9 mg of maleimide following the general procedure provided **3n** as an off-white solid after column chromatography using 0–4% methanol in dichloromethane (17.2 mg, 30%). Spectroscopic data were in accordance with the literature;^66^^1^H NMR (400 MHz, chloroform-*d*): δ 8.56 (s, 1H), 7.50–7.36 (m, 1H), 7.25–7.17 (m, 1H), 6.89 (td, *J* = 7.4, 1.1 Hz, 1H), 6.72 (dd, *J* = 8.3, 1.1 Hz, 1H), 4.02 (d, *J* = 9.5 Hz, 1H), 3.50 (dd, *J* = 11.5, 2.6 Hz, 1H), 3.41 (ddd, *J* = 9.5, 4.5, 2.6 Hz, 1H), 3.02 (ddd, *J* = 11.5, 4.4, 0.8 Hz, 1H), 2.81 (s, 3H) ppm; ^13^C{^1^H} NMR (101 MHz, chloroform-*d*): δ 179.0, 177.0, 148.5, 130.2, 128.8, 119.9, 118.5, 112.7, 50.5, 44.9, 43.4, 39.6 ppm.

#### 5,8-Dimethyl-2-phenyl-3a,4,5,9b-tetrahydro-1*H*-pyrrolo[3,4-*c*]quinoline-1,3(2*H*)-dione (**3o**)^7^

Starting with 43.9 mg of *N*-phenylmaleimide following the general procedure provided a white solid (65.0 mg, 84%) after purification using column chromatography (SiO_2_, 0–10% ethyl acetate in petroleum ether). Spectroscopic data were in accordance with the literature;^7^^1^H NMR (400 MHz, chloroform-*d*): δ 7.46–7.38 (m, 2H), 7.37–7.30 (m, 2H), 7.29–7.23 (m, 2H), 7.07–6.98 (m, 1H), 6.65 (d, *J* = 8.3 Hz, 1H), 4.11 (d, *J* = 9.5 Hz, 1H), 3.58 (dd, *J* = 11.4, 2.7 Hz, 1H), 3.50 (ddd, *J* = 9.6, 4.4, 2.7 Hz, 1H), 3.05 (dd, *J* = 11.4, 4.4 Hz, 1H), 2.79 (s, 3H), 2.29 (s, 3H) ppm; ^13^C{^1^H} NMR (101 MHz, chloroform-*d*): δ 177.9, 176.0, 146.4, 132.1, 131.0, 129.4, 129.2, 129.1, 128.6, 126.5, 118.7, 112.7, 51.1, 43.7, 42.3, 39.8, 20.6 ppm.

#### 5,7,9-Trimethyl-2-phenyl-3a,4,5,9b-tetrahydro-1*H*-pyrrolo[3,4-*c*]quinoline-1,3(2*H*)-dione (**3p**)^22^

Starting with 42.4 mg of *N*-phenylmaleimide following the general procedure provided **3p** as a yellow solid (41.8 mg, 53%) after purification using column chromatography (SiO_2_, 0–10% ethyl acetate in petroleum ether). Spectroscopic data were in accordance with the literature;^22^^1^H NMR (400 MHz, chloroform-*d*): δ 7.44–7.37 (m, 2H), 7.36–7.29 (m, 1H), 7.28–7.22 (m, 2H), 6.71–6.36 (m, 2H), 4.45 (d, *J* = 9.8 Hz, 1H), 3.54 (dd, *J* = 11.3, 1.6 Hz, 1H), 3.48 (ddd, *J* = 9.8, 4.8, 1.7 Hz, 1H), 2.91 (dd, *J* = 11.3, 4.8 Hz, 1H), 2.76 (s, 3H), 2.53 (s, 3H), 2.27 (s, 3H) ppm; ^13^C{^1^H} NMR (101 MHz, chloroform-*d*): δ 178.7, 176.0, 150.1, 138.4, 137.9, 132.2, 129.1, 128.6, 126.6, 123.7, 116.6, 111.6, 52.7, 44.8, 40.0, 39.4, 21.7, 20.4 ppm.

#### 8-Bromo-5-methyl-2-phenyl-3a,4,5,9b-tetrahydro-1*H*-pyrrolo[3,4-*c*]quinoline-1,3(2*H*)-dione (**3q**)^43^

Starting with 46.3 mg of *N*-phenylmaleimide following the general provided **3q** as a yellow solid (69.3 mg, 70%) after purification using column chromatography (SiO_2_, 0–10% ethyl acetate in petroleum ether). Spectroscopic data were in accordance with the literature;^43^^1^H NMR (400 MHz, chloroform-*d*): δ 7.64 (m, 1H), 7.48–7.39 (m, 2H), 7.40–7.33 (m, 1H), 7.31 (m, 1H), 7.29–7.21 (m, 2H), 6.60 (dd, *J* = 8.8, 1.9 Hz, 1H), 4.10 (dd, *J* = 9.6, 1.8 Hz, 1H), 3.60 (m, 1H), 3.56–3.45 (m, 1H), 3.11 (m, 1H), 2.82 (d, *J* = 1.9 Hz, 3H) ppm; ^13^C{^1^H} NMR (101 MHz, chloroform-*d*): δ 177.4, 175.2, 147.6, 132.8, 131.9, 131.6, 129.2, 128.8, 126.4, 120.5, 114.4, 111.8, 50.5, 43.4, 41.9, 39.6 ppm.

#### 5-Methyl-2-phenyl-8-(pyridin-2-yl)-3a,4,5,9b-tetrahydro-1*H*-pyrrolo[3,4-*c*]quinoline-1,3(2*H*)-dione (**3r**)

Starting with 18.1 mg of *N*-phenylmaleimide following the general procedure using an irradiation time of 18 h provided **3r** as a yellow foam (26.4 mg, 68%) after purification using column chromatography (SiO_2_, 0–10% ethyl acetate in petroleum ether). mp 183.5–184.6 °C; ^1^H NMR (400 MHz, chloroform-*d*): δ 8.16 (p, *J* = 1.0 Hz, 1H), 7.90 (dt, *J* = 8.7, 1.7 Hz, 1H), 7.69 (dt, *J* = 4.9, 1.4 Hz, 2H), 7.40 (ddd, *J* = 7.6, 6.6, 1.4 Hz, 2H), 7.37–7.29 (m, 1H), 7.26 (dt, *J* = 8.3, 1.4 Hz, 3H), 7.14 (qd, *J* = 4.6, 1.3 Hz, 1H), 6.82 (dd, *J* = 8.6, 1.3 Hz, 1H), 4.28–4.18 (m, 1H), 3.64 (ddd, *J* = 11.5, 2.9, 1.3 Hz, 1H), 3.56 (dddd, *J* = 8.7, 4.3, 2.8, 1.3 Hz, 1H), 3.18 (ddd, *J* = 11.5, 4.4, 1.3 Hz, 1H), 2.89 (s, 3H) ppm; ^13^C{^1^H} NMR (101 MHz, chloroform-*d*): δ 177.6, 175.8, 157.0, 149.4, 149.2, 137.0, 132.1, 130.6, 129.1, 129.0, 128.7, 127.5, 126.5, 121.5, 120.0, 118.5, 113.0, 50.4, 43.5, 42.2, 39.6 ppm; FTIR ν: 1704, 1568, 1469, 1370, 1152, 776, 761, 691, 576 cm^–1^; HRMS (ESI) *m*/*z*: calcd C_23_H_19_N_3_O_2_ [M + H]^+^, 370.1556; found, 370.1556.

#### 8-Acetyl-5-methyl-2-phenyl-3a,4,5,9b-tetrahydro-1*H*-pyrrolo[3,4-*c*]quinoline-1,3(2*H*)-dione (**3s**)^21^

Starting with 43.5 mg of *N*-phenylmaleimide following the general procedure, **3s** was obtained as an off-white solid (39.5 mg, 47%) after purification using column chromatography (SiO_2_, 0–10% ethyl acetate in petroleum ether). Spectroscopic data were in accordance with the literature;^21^^1^H NMR (400 MHz, chloroform-*d*): δ 8.13 (dd, *J* = 2.2, 0.9 Hz, 1H), 7.85 (dd, *J* = 8.7, 2.1 Hz, 1H), 7.47–7.39 (m, 2H), 7.39–7.32 (m, 1H), 7.28–7.20 (m, 2H), 6.74 (d, *J* = 8.7 Hz, 1H), 4.19 (dd, *J* = 9.5, 0.9 Hz, 1H), 3.67 (dd, *J* = 11.7, 3.0 Hz, 1H), 3.58 (ddd, *J* = 9.6, 4.5, 2.9 Hz, 1H), 3.25 (dd, *J* = 11.7, 4.5 Hz, 1H), 2.93 (s, 3H), 2.54 (s, 3H) ppm; ^13^C{^1^H} NMR (101 MHz, chloroform-*d*): δ 196.5, 177.0, 175.4, 151.8, 131.9, 131.3, 129.6, 129.1, 128.7, 128.6, 126.3, 117.2, 112.1, 49.6, 43.0, 41.7, 39.5, 26.3 ppm.

#### Ethyl-5-methyl-1,3-dioxo-2-phenyl-2,3,3a,4,5,9b-hexahydro-1*H*-pyrrolo[3,4-*c*]quinoline-8-carboxylate (**3t**)

Starting with 43.3 mg of *N*-phenylmaleimide following the general procedure, the product was obtained as an off-white solid after silica column chromatography using 0–10% EtOAc in petroleum ether. Subsequent recrystallization from hot ethyl acetate afforded compound **3t** as a yellow crystalline solid (26.9 mg, 30%). mp 156.5–157.8 °C; ^1^H NMR (400 MHz, chloroform-*d*): δ 8.21 (d, *J* = 2.0 Hz, 1H), 7.91 (dd, *J* = 8.7, 2.0 Hz, 1H), 7.42 (dd, *J* = 8.3, 6.6 Hz, 2H), 7.38–7.32 (m, 1H), 7.25 (dd, *J* = 7.4, 1.6 Hz, 2H), 6.73 (d, *J* = 8.7 Hz, 1H), 4.42–4.27 (m, 2H), 4.19 (d, *J* = 9.4 Hz, 1H), 3.67 (dd, *J* = 11.6, 2.5 Hz, 1H), 3.62–3.51 (m, 1H), 3.22 (dd, *J* = 11.7, 4.2 Hz, 1H), 2.92 (s, 3H), 1.37 (t, *J* = 7.1 Hz, 3H) ppm; ^13^C{^1^H} NMR (101 MHz, chloroform-*d*): δ 177.2, 175.4, 166.5, 151.7, 131.9, 131.9, 130.7, 129.1, 128.7, 126.4, 121.3, 117.4, 112.1, 60.7, 49.9, 43.2, 41.8, 39.5, 14.6 ppm; ATR-FTIR ν: 1709, 1700, 1606, 1517, 1500, 1373, 1272, 1178, 1152, 759 cm^–1^; HRMS (ESI+) *m*/*z*: calcd C_21_H_20_N_2_O_4_ [M + H]^+^, 365.1501; found, 365.1501.

#### 5-Methyl-1,3-dioxo-2-phenyl-2,3,3a,4,5,9b-hexahydro-1*H*-pyrrolo[3,4-*c*]quinoline-8-carbonitrile (**3u**)

Starting with 39.7 mg of *N*-phenylmaleimide following the general procedure provided **3u** as a yellow solid (18 mg, 25%) after purification using column chromatography (SiO_2_, 0–10% ethyl acetate in petroleum ether). mp 194.0–194.9 °C; ^1^H NMR (400 MHz, chloroform-*d*): δ 7.82 (dd, *J* = 2.1, 0.8 Hz, 1H), 7.51–7.41 (m, 3H), 7.41–7.35 (m, 1H), 7.29–7.20 (m, 2H), 6.74 (d, *J* = 8.6 Hz, 1H), 4.16 (dd, *J* = 9.6, 0.8 Hz, 1H), 3.69 (dd, *J* = 11.8, 3.1 Hz, 1H), 3.59 (ddd, *J* = 9.6, 4.5, 3.1 Hz, 1H), 3.29 (dd, *J* = 11.8, 4.5 Hz, 1H), 2.94 (s, 3H) ppm; ^13^C{^1^H} NMR (101 MHz, chloroform-*d*): δ 176.7, 174.8, 151.2, 134.2, 133.1, 131.7, 129.3, 128.9, 126.3, 119.6, 118.3, 112.8, 101.8, 49.4, 42.8, 41.4, 39.5 ppm; FTIR ν: 3066, 2931, 2217, 1710, 1604, 1514, 1497, 1374 cm^–1^; HRMS (ESI) *m*/*z*: calcd C_19_H_15_N_3_O_2_ [M + H]^+^, 318.1243; found, 318.1253.

#### 5,6-Dimethyl-2-phenyl-3a,4,5,9b-tetrahydro-1*H*-pyrrolo[3,4-*c*]quinoline-1,3(2*H*)-dione (**3v**)^35^

Yellow oil (32.9 mg, 43%) obtained after purification using column chromatography (SiO_2_, 0–10% ethyl acetate in petroleum ether). Spectroscopic data were in accordance with the literature;^35^^1^H NMR (400 MHz, chloroform-*d*): δ 7.57 (ddt, *J* = 7.6, 1.6, 0.7 Hz, 1H), 7.49–7.41 (m, 2H), 7.41–7.34 (m, 1H), 7.29–7.23 (m, 2H), 7.14 (ddt, *J* = 7.4, 1.6, 0.7 Hz, 1H), 7.06 (t, *J* = 7.5 Hz, 1H), 4.16 (d, *J* = 8.9 Hz, 1H), 3.60–3.45 (m, 2H), 3.45–3.32 (m, 1H), 2.74 (s, 3H), 2.30 (s, 3H) ppm; ^13^C{^1^H} NMR (101 MHz, chloroform-*d*): δ 177.9, 175.9, 146.8, 132.9, 132.1, 130.6, 129.3, 129.1, 128.8, 128.6, 126.4, 124.0, 123.1, 51.5, 42.2, 41.7, 39.0, 17.8 ppm.

#### 8-Ethynyl-5-methyl-2-phenyl-3a,4,5,9b-tetrahydro-1*H*-pyrrolo[3,4-*c*]quinoline-1,3(2*H*)-dione (**3w**)^21^

Starting with 41.3 mg of *N*-phenylmaleimide following the general procedure provided **3w** as a yellow solid (19 mg, 25%) after purification using column chromatography (SiO_2_, 0–10% ethyl acetate in petroleum ether). Spectroscopic data were in accordance with the literature;^21^^1^H NMR (400 MHz, chloroform-*d*): δ 7.68 (dd, *J* = 2.0, 0.9 Hz, 1H), 7.49–7.40 (m, 2H), 7.40–7.31 (m, 2H), 7.30–7.21 (m, 3H), 6.67 (d, *J* = 8.5 Hz, 1H), 4.12 (dt, *J* = 9.6, 0.6 Hz, 1H), 3.63 (dd, *J* = 11.6, 2.8 Hz, 1H), 3.54 (ddd, *J* = 9.6, 4.4, 2.8 Hz, 1H), 3.17 (dd, *J* = 11.6, 4.4 Hz, 1H), 3.00 (s, 1H), 2.86 (s, 3H) ppm; ^13^C{^1^H} NMR (101 MHz, chloroform-*d*): δ 177.4, 175.4, 148.7, 134.2, 132.8, 132.0, 129.2, 128.7, 126.4, 118.2, 112.8, 112.6, 83.9, 76.1, 50.2, 43.3, 41.8, 39.5 ppm.

#### 5-Benzyl-2-phenyl-3a,4,5,9b-tetrahydro-1*H*-pyrrolo[3,4-*c*]quinoline-1,3(2*H*)-dione (**3x**)^20^

Starting with 40.5 mg of *N*-phenylmaleimide following the general procedure provided **3x** as a yellow oil (36.6 mg, 42%) after purification using column chromatography (SiO_2_, 0–10% ethyl acetate in petroleum ether). Spectroscopic data were in accordance with the literature;^20^^1^H NMR (400 MHz, chloroform-*d*): δ 7.60–7.52 (m, 1H), 7.47 (dd, *J* = 8.3, 6.8 Hz, 2H), 7.43–7.36 (m, 1H), 7.34–7.22 (m, 7H), 7.19–7.11 (m, 1H), 6.90 (td, *J* = 7.5, 1.1 Hz, 1H), 6.76 (d, *J* = 1.0 Hz, 1H), 4.55–4.25 (m, 2H), 4.19 (d, *J* = 9.5 Hz, 1H), 3.70 (dd, *J* = 11.7, 2.9 Hz, 1H), 3.55 (ddd, *J* = 9.5, 4.4, 2.8 Hz, 1H), 3.28 (dd, *J* = 11.7, 4.4 Hz, 1H) ppm; ^13^C{^1^H} NMR (101 MHz, chloroform-*d*): δ 177.6, 175.9, 147.6, 137.8, 132.2, 130.7, 130.3, 129.2, 128.7, 128.7, 128.6, 127.5, 127.4, 126.5, 119.9, 119.0, 113.6, 55.5, 49.2, 44.3, 42.6 ppm.

#### 5-Allyl-2-phenyl-3a,4,5,9b-tetrahydro-1*H*-pyrrolo[3,4-*c*]quinoline-1,3(2*H*)-dione (**3y**)^20^

Starting with 41.6 mg of *N*-phenylmaleimide following the general procedure provided **3y** as a yellow oil (44.1 mg, 58%) after purification using column chromatography (SiO_2_, 0–10% ethyl acetate in petroleum ether). Spectroscopic data were in accordance with the literature;^20^^1^H NMR (400 MHz, chloroform-*d*): δ 7.54 (ddd, *J* = 7.6, 1.7, 0.8 Hz, 1H), 7.47–7.40 (m, 2H), 7.40–7.33 (m, 1H), 7.30–7.23 (m, 2H), 7.24–7.16 (m, 1H), 6.89 (td, *J* = 7.5, 1.1 Hz, 1H), 6.78 (dd, *J* = 8.3, 1.1 Hz, 1H), 5.87 (ddt, *J* = 17.3, 10.3, 5.8 Hz, 1H), 5.34–5.21 (m, 2H), 4.15 (d, *J* = 9.5 Hz, 1H), 3.96–3.74 (m, 2H), 3.68 (dd, *J* = 11.8, 2.9 Hz, 1H), 3.54 (ddd, *J* = 9.5, 4.3, 2.8 Hz, 1H), 3.15 (dd, *J* = 11.8, 4.3 Hz, 1H) ppm; ^13^C{^1^H} NMR (101 MHz, chloroform-*d*): δ 177.7, 175.9, 147.4, 133.3, 132.1, 130.7, 129.1, 128.6, 128.6, 126.4, 119.6, 118.9, 118.3, 113.3, 53.6, 47.8, 44.1, 42.5 ppm.

#### 5,9-Dimethyl-2-phenyl-3a,4,5,9b-tetrahydro-1*H*-pyrrolo[3,4-*c*]quinoline-1,3(2*H*)-dione (**3aa**) and 5,7-Dimethyl-2-phenyl-3a,4,5,9b-tetrahydro-1*H*-pyrrolo[3,4-*c*]quinoline-1,3(2*H*)-dione (**3aa′**)^22^

Starting with 41.6 mg of *N*-phenylmaleimide following the general procedure provided a mixture of regioisomers **3aa** and **3aa′** as an off-white solid (31.8 mg, 42%) after purification using column chromatography (SiO_2_, 0–7% ethyl acetate in petroleum ether). Spectroscopic data were in accordance with the literature;^22^^1^H NMR (400 MHz, chloroform-*d*): δ 7.46–7.39 (m, 4H), 7.38–7.32 (m, 1.6H), 7.32–7.26 (m, 3H), 7.13 (t, *J* = 7.9 Hz, 1H), 6.82 (d, *J* = 7.5 Hz, 1H), 6.77–6.70 (m, 0.6H), 6.64 (d, *J* = 8.2 Hz, 1H), 6.56 (d, *J* = 1.5 Hz, 0.6H), 4.53 (d, *J* = 9.8 Hz, 1H), 4.13 (d, *J* = 9.6 Hz, 0.6H), 3.63–3.57 (m, 1.8H), 3.56–3.49 (m, 1.6H), 3.11 (dd, *J* = 11.5, 4.4 Hz, 1H), 2.96 (dd, *J* = 11.3, 4.8 Hz, 1H), 2.83 (s, 1.6H), 2.79 (s, 3H), 2.59 (s, 3H), 2.33 (s, 1.6H) ppm; ^13^C{^1^H} NMR (101 MHz, chloroform-*d*): δ 178.7, 178.0, 176.1, 175.8, 150.2, 148.5, 138.74, 138.69, 132.2, 132.1, 130.3, 129.2, 129.1, 128.7, 128.6, 128.2, 126.6, 126.5, 122.8, 120.7, 119.6, 115.7, 113.4, 110.8, 52.7, 50.8, 45.0, 43.7, 42.0, 40.0, 39.62, 39.60, 21.8, 20.5 ppm.

#### 1-Methyl-1,2,3,4-tetrahydroquinoline-3,4-dicarbonitrile (**5**)

Starting with fumaronitrile (18.7 mg) following the general procedure, **5** was afforded as a white solid (8.9 mg, 19%) after purification using column chromatography (SiO_2_, 0–10% ethyl acetate in petroleum ether). ^1^H NMR (400 MHz, chloroform-*d*): δ 7.33–7.14 (m, 3H), 6.81 (td, *J* = 7.5, 1.1 Hz, 1H), 6.72 (dd, *J* = 8.3, 1.0 Hz, 1H), 4.25 (d, *J* = 5.7 Hz, 1H), 3.69 (dd, *J* = 12.0, 3.1 Hz, 1H), 3.59–3.41 (m, 2H), 3.01 (s, 3H); ^13^C{^1^H} NMR (101 MHz, chloroform-*d*): δ 144.4, 130.7, 129.5, 118.5, 118.2, 117.4, 112.8, 111.6, 50.1, 39.2, 32.9, 28.8 ppm; FTIR ν: 2843, 2247, 1601, 1507, 1349, 1310, 1223, 740 cm^–1^; HRMS (ESI) *m*/*z*: calcd C_12_H_11_N_3_ [M + H]^+^, 198.1031; found, 198.1031.

#### 1-Methyl-3-phenyl-2,3-dihydroquinoline-4,4(1*H*)-dicarbonitrile (**6**)^65^

Starting with benzylidenemalonitrile (34 mg), **6** was afforded as a white solid (9.9 mg, 17%) after purification using column chromatography (SiO_2_, 0–10% ethyl acetate in petroleum ether). Spectroscopic data were in accordance with the literature;^65^^1^H NMR (400 MHz, chloroform-*d*): δ 7.91 (ddd, *J* = 8.5, 1.3, 0.6 Hz, 1H), 7.51–7.42 (m, 5H), 7.39–7.32 (m, 1H), 6.82 (ddd, *J* = 7.8, 7.3, 1.1 Hz, 1H), 6.75 (dd, *J* = 8.4, 1.1 Hz, 1H), 3.97 (dd, *J* = 12.4, 11.4 Hz, 1H), 3.61 (dd, *J* = 11.4, 3.9 Hz, 1H), 3.52 (dd, *J* = 12.4, 3.9 Hz, 1H), 3.03 (s, 3H); ^13^C{^1^H} NMR (101 MHz, chloroform-*d*): δ 144.1, 134.9, 131.9, 129.6, 129.3, 129.0, 128.6, 117.8, 115.3, 114.2, 112.9, 112.7, 51.5, 45.7, 42.6, 38.9 ppm.

#### Diethyl (3*R*,4*R*)-1-Methyl-1,2,3,4-tetrahydroquinoline-3,4-dicarboxylate (**7**)^28^

Starting with diethyl fumarate (25.8 mg), following the general procedure, **7** was obtained as a colorless oil (13 mg, 29%) after purification using column chromatography (SiO_2_, 0–10% ethyl acetate in petroleum ether). Spectroscopic data were in accordance with the literature;^28^^1^H NMR (400 MHz, chloroform-*d*): δ 7.22 (dt, *J* = 7.7, 1.4 Hz, 1H), 7.14 (dddd, *J* = 8.1, 7.3, 1.7, 0.7 Hz, 1H), 6.81–6.58 (m, 2H), 4.30–4.07 (m, 5H), 3.59–3.49 (m, 1H), 3.46–3.34 (m, 2H), 2.92 (s, 3H), 1.29 (t, *J* = 7.1 Hz, 3H), 1.23 (t, *J* = 7.1 Hz, 3H) ppm; ^13^C{^1^H} NMR (101 MHz, chloroform-*d*): δ 173.3, 172.2, 145.8, 129.2, 128.5, 117.9, 117.3, 112.0, 61.4, 61.2, 50.4, 44.8, 41.3, 39.5, 14.3 (2C) ppm.

## Supporting Information Available

UV–vis studies, emission spectrum of the irradiation source, mechanistic investigations, and NMR spectra (PDF)
