# Supplementary material for: Aerobic Oxidative EDA Catalysis: Synthesis of Tetrahydroquinolines Using an Organocatalytic EDA Active Acceptor
Source: J Org Chem. 2022 Jan 10;87(2):1457–69. doi: 10.1021/acs.joc.1c02776 (PMC8790759; doi:10.1021/acs.joc.1c02776)
Supplement: Supplementary file 1 — jo1c02776_si_001.pdf [file jo1c02776_si_001.pdf]

Supporting Information for

Aerobic oxidative EDA catalysis: Synthesis of tetrahydroquinolines using an organocatalytic EDA active acceptor

August Runemark[a] and Henrik Sundén[b*]

[a]Department of Chemistry and Chemical Engineering, Chalmers University of Technology, Kemivägen 10, 41296 Goothenburg, Sweden
[b]Chemistry and Molecular Biology, University of Gothenburg, Kemivägen 10, 412 96, Gothenburg, Sweden.
* E-mail: henrik.sunden@chem.gu.se

Contents

1.1 UV-vis spectrum of **1a**, **2a**, **4a**, and their mixtures S2
1.2 Emission spectrum of the irradiation source S2
1.3 Investigation of the background reaction without catalyst S3
1.4 Reaction profile with and without **4a** S4
1.5 Kinetic Isotope Effect investigations S5
1.6 Competition experiments S7
1.7 Measurement of oxygen consumption S8
1.8 Quantum yield determination S9
2.1 References S10
2.1 NMR spectra S12

1.1 UV-vis spectrum of **1a**, **2a**, **4a**, and their mixtures

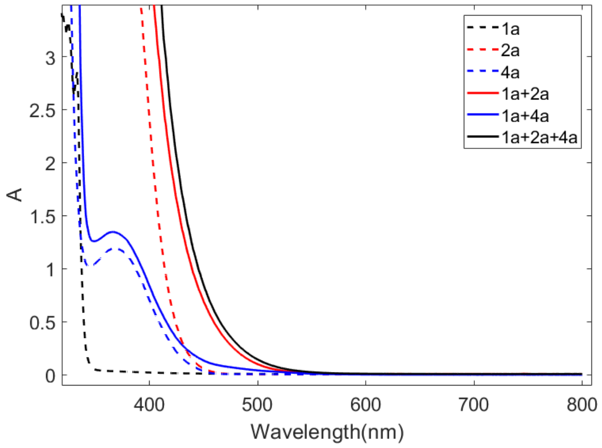

*Figure S1. UV-vis spectrum (300 – 800 nm) of **1a** (0.4 M), **2a** (73 mM), **4a** (6 mM), and their EDA complexes in 1,4-dioxane.*

1.2 Emission spectrum of the irradiation source

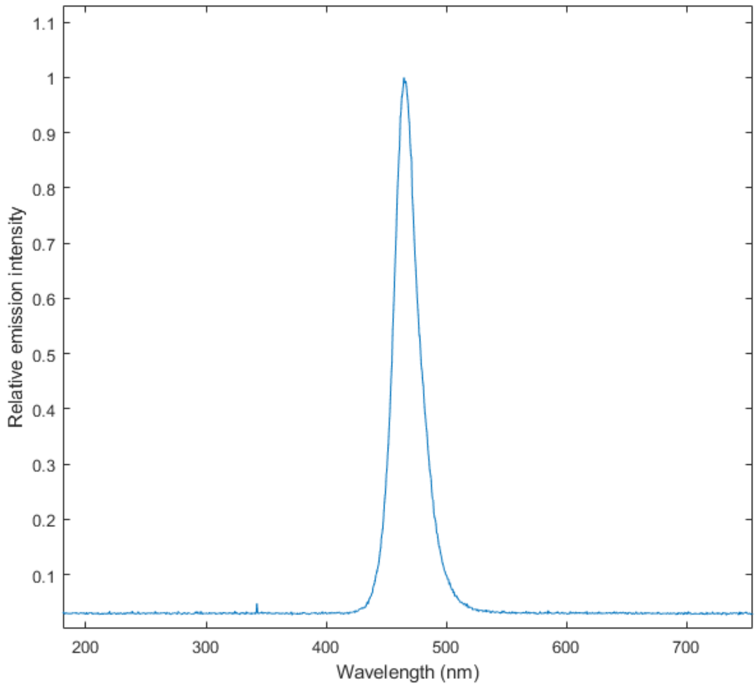

Figure S2. The normalized emission spectrum of the blue LED stripe used for the photoreaction, with λ_{max} of 465 nm

1.3 Investigation of the background reaction without catalyst

For each entry where the possibility of maleimide-amine EDA complex formation, a control experiment was carried out without the catalyst **4a**, under otherwise identical conditions (Scheme S1).

Scheme S1. Background reaction between maleimides and N,N-dialkylanilines under irradiation with blue LED.

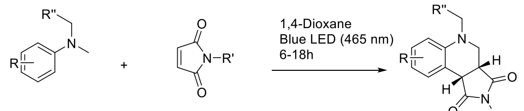

7 eq. | 0.3 mmol | **3a-z**

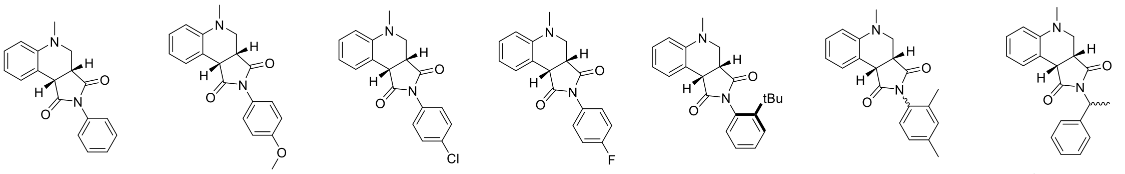

3a, 7% | **3b**, 10% | **3c**, 30% | **3d**, 4% | **3e**, 19% | **3f**, 3% | **3g**, 28%

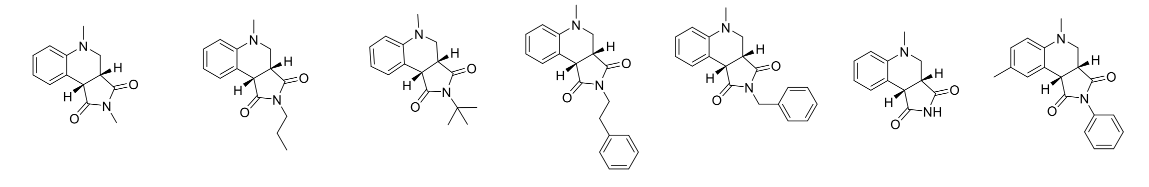

3h, 3% | **3i**, 4% | **3k**, 9% | **3l**, 4% | **3m**, 15% | **3n**, 3% | **3o**, 10%

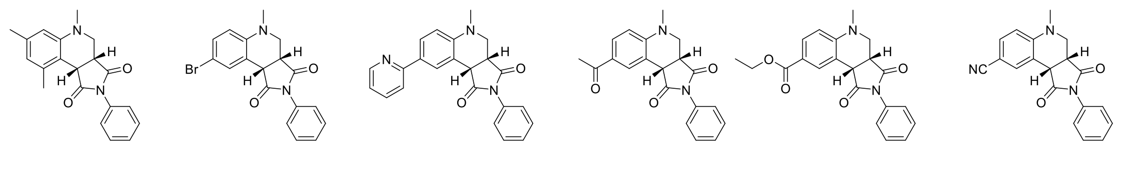

3p, n.d.[a] | **3q**, n.d[a, b] | **3r**, 7%[b] | **3s**, n.d.[a] | **3t**, n.d.[a] | **3u**, n.d[a]

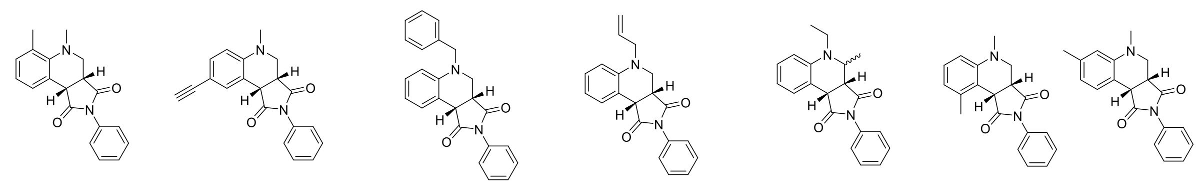

3v, n.d[a] | **3w**, 7% | **3x**, n.d.[a] | **3y**, n.d[a] | **3z**, n.d.[a] | **3aa/3aa'**, n.d.[a]

Conditions: maleimide (0.3 mmol) and amine (7 equiv.) in 3 mL 1,4-dioxane was irradiated for 6-18 hours under ambient atmosphere. Yields reported are isolated. a) No, or trace amounts, of product was observed in the crude reaction mixture by ^{1}H NMR analysis. b) Reaction time 18 hours.

1.4 Reaction profile with and without **4a**

The formation of **3a** when using a 40W blue (440 nm) LED was followed over time in the presence and absence of **4a** as catalyst (Figure S3). It was observed that the formation of product was significantly faster in the presence of the catalyst.

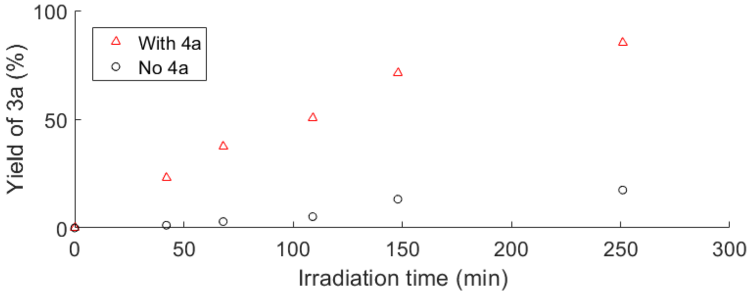

*Figure S3. Yield of product **3a** over time in presence (red triangles) and absence (black circles) of the catalyst **4a**. Measured as duplicates.*

1.5 Kinetic Isotope Effect investigations

Intramolecular KIE

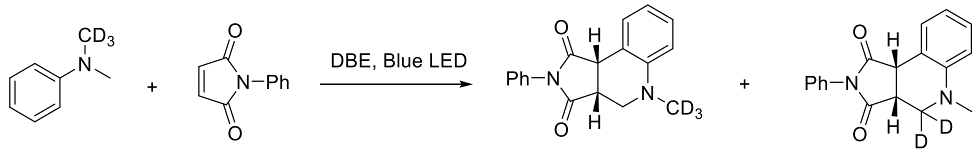

Following a modified published procedure,[3] *N*-phenylmaleimide (7.6 mg, 0.044 mmol, 1 equiv.), *N*-(CD_3)-*N*-(CH_3)-aniline (20.5 mg, 0.17 mmol, 3.8 equiv.) and 1,2-dibenzoyl ethylene (0.8 mg, 0.0034 mmol, 0.08 equiv.) were added to a 2-5 mL Biotage microwave vial. 1,4-Dioxane (1 mL) was then added and the open flask reaction mixture was irradiated using 465 nm LED strip for 4 hours. Solvent was then removed under reduced pressure and the residue dried in vacuo. The resulting crude product mixture was then dissolved in deuterated chloroform and was analyzed by ^{1}H NMR. Based on comparison of the integrals of the N(CH_3) group and the CH_2 group, the intramolecular KIE was estimated to be **5.8**.

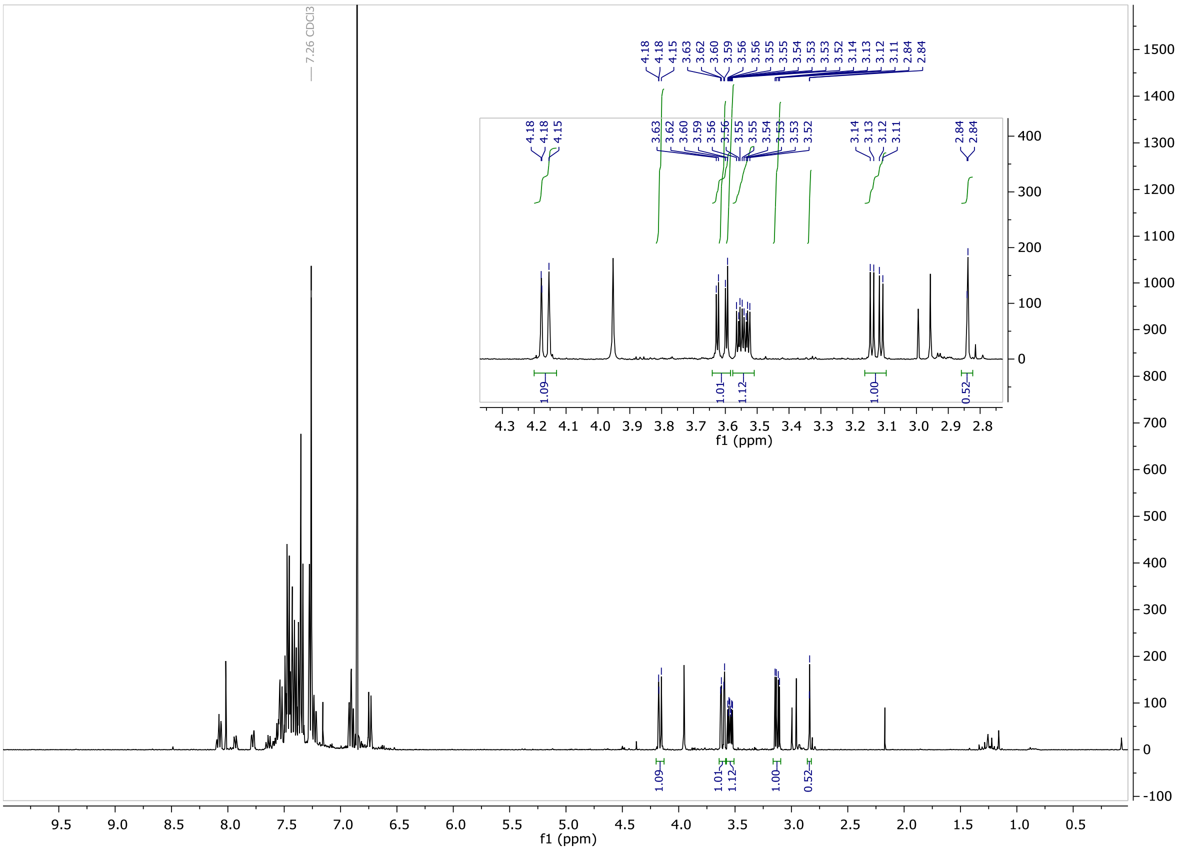

Intermolecular KIE

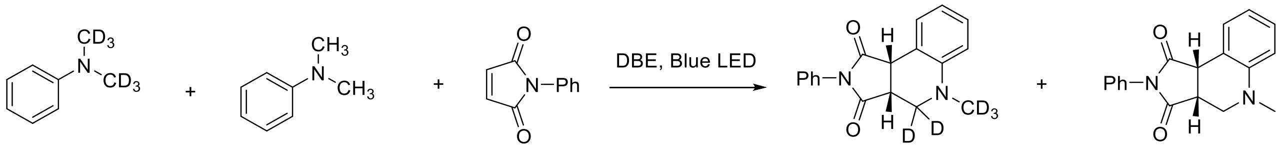

Following a modified published procedure,[3] *N*-phenylmaleimide (8.0 mg, 0.046 mmol, 1 equiv.), *N,N*-$(CD_3)_2$-aniline (14.6 mg, 0.12 mmol, 2.5 equiv.), *N,N*-dimethylaniline (13.9 mg, 0.12 mmol, 2.5 equiv.), and 1,2-dibenzoyl ethylene (0.8 mg, 0.0034 mmol, 0.08 equiv.) were added to a 2-5 mL Biotage microwave vial. 1,4-Dioxane (1 mL) was then added, and the reaction mixture was irradiated using 465 nm LED strip for 5 hours. Solvent was then removed under reduced pressure and the residue dried in vacuo. The resulting crude product mixture was then dissolved in deuterated chloroform and was analyzed by ^{1}H NMR. Based on comparison of the integrals of the $N(CH_3)$ group and the benzylic CH group, the intramolecular KIE was estimated to be **3.8**.

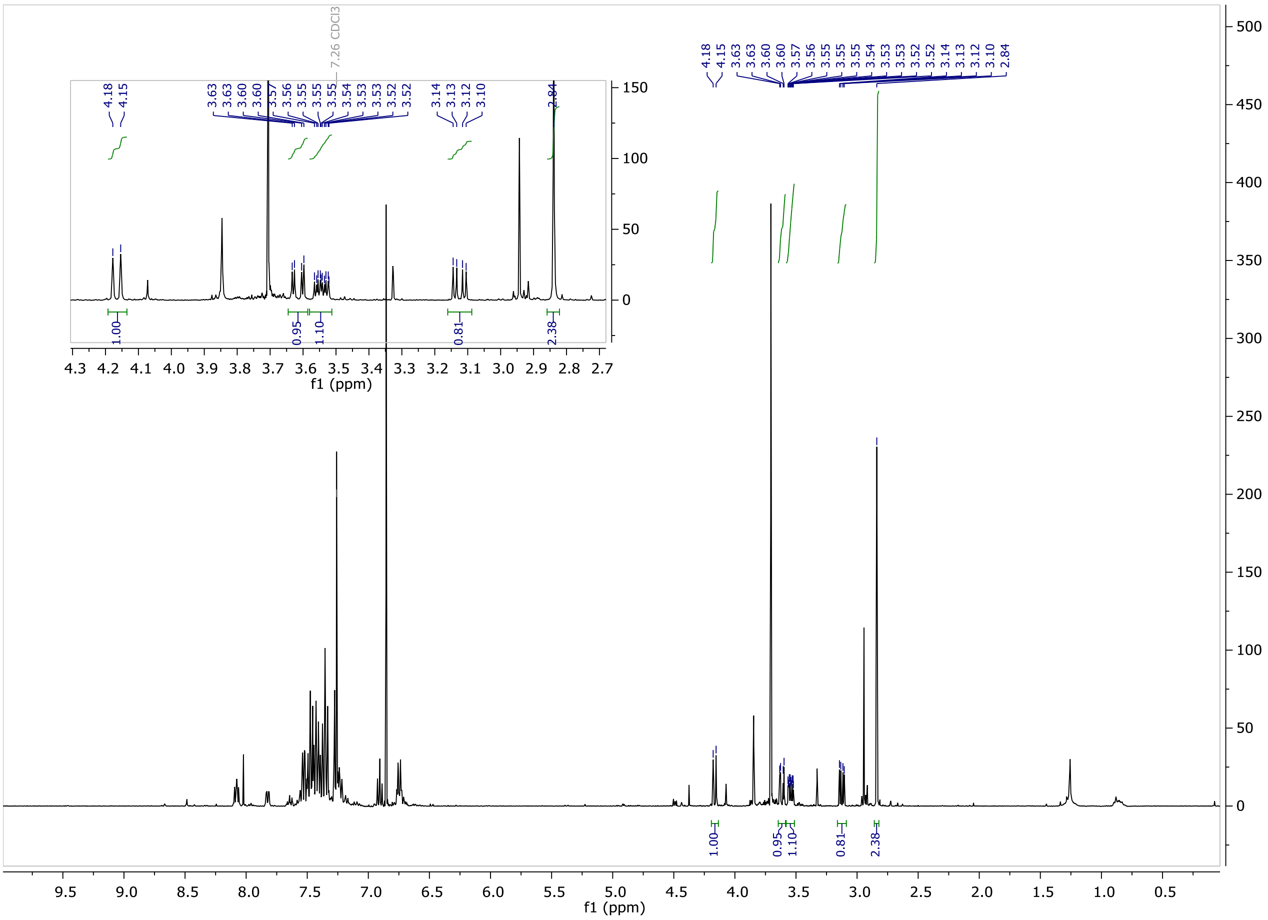

1.6 Competition experiments

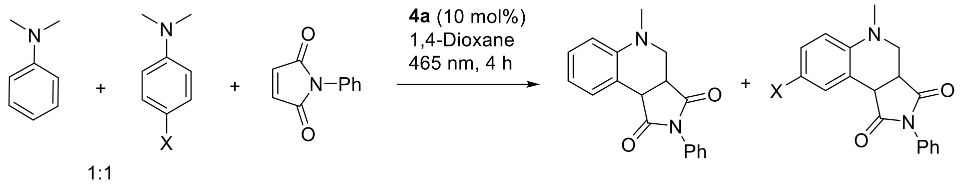

To investigate the impact of substituents on the amine reaction partner, four reactions were set up:

A: To a 2-5 mL Biotage microwave vial was added **2a** (18.4 mg, 0.11 mmol), **1a** (35 mg, 0.29 mmol), 4'-*N,N*-trimethyl aniline (39 mg, 0.29 mmol), **4a** (2.2 mg, 0.009 mmol) and 3 mL 1,4-Dioxane.

B: To a 2-5 mL Biotage microwave vial was added **2a** (17.7 mg, 0.10 mmol), **1a** (24 mg, 0.20 mmol), *p*-CN-*N,N*-dimethyl aniline (29 mg, 0.20 mmol), **4a** (2.2 mg, 0.009 mmol) and 3 mL 1,4-Dioxane.

C: To a 2-5 mL Biotage microwave vial was added **2a** (17.1 mg, 0.10 mmol), **1a** (25 mg, 0.21 mmol), *p*-Br-*N,N*-dimethyl aniline (42.8 mg, 0.21 mmol), **4a** (2.2 mg, 0.009 mmol) and 3 mL 1,4-Dioxane.

D: To a 2-5 mL Biotage microwave vial was added **2a** (16.8 mg, 0.97 mmol), **1a** (27 mg, 0.22 mmol), *p*-COMe-*N,N*-dimethyl aniline (36.0 mg, 0.22 mmol), **4a** (2.2 mg, 0.009 mmol) and 3 mL 1,4-Dioxane.

The four reactions where then irradiated using 465 nm LED strip for 4 hours. The ratio of the products was then calculated by ^{1}H NMR analysis of the crude reaction mixture. To establish the electronic effect, the natural logarithm of the ratio was plotted against the Hammett σ_p parameter (Figure S4).[2]

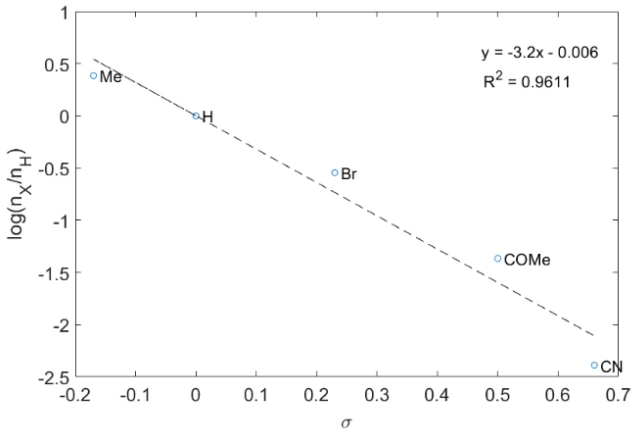

Figure S4. Plot of the ratio of products n(X)/n(H) plotted against the Hammett parameter σ_p.

1.7 Measurement of oxygen consumption

To estimate the amount of oxygen consumed in the photoreaction, an in-house made set up was prepared consisting of two vertically placed 20 mL burettes filled to half heigh with water and connected at the bottom with a rubber tube. One of the burettes was connected to a Schlenk tube containing the reaction mixture with a rubber tube. All fittings were carefully sealed with Teflon tape to ensure a closed system. The other burette was left open to the atmosphere at the top. The reaction mixture was then irradiated. The gas volume displaced by water as the oxygen in the closed system was consumed, was monitored over time. Aliquots of the reaction mixture was taken using a Hamilton 20 µL syringe for GC-FID analysis at regular time intervals. Based on the atmospheric pressure measured in the house (103 hPa) and the volume of displaced gas, the molar amount of oxygen was estimated using the ideal gas law. It was assumed that all gas displaced was due to consumption of oxygen. The amount of product **3a** formed was then plotted against the molar amount of oxygen consumed (Figure S6). A slope of 1.3 was observed, suggesting that 1.3 molecules of dioxygen is consumed per product formed.

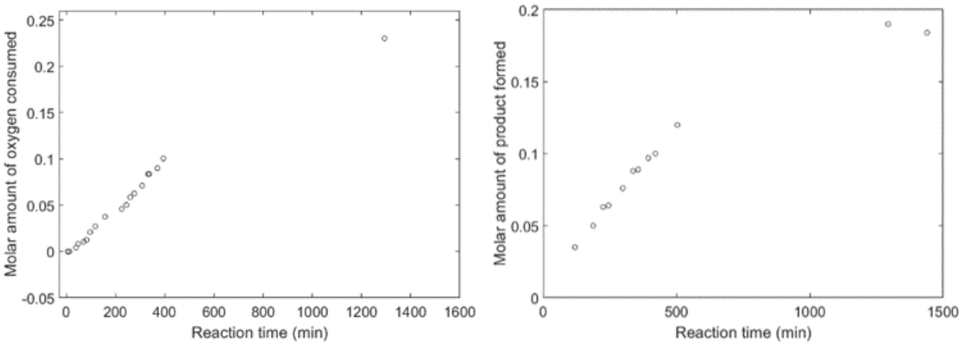

*Figure S5. Consumption of oxygen (left) and formation of **3a** (right) over time. Amount of product was determined using GC-FID using n-dodecane as the internal standard.*

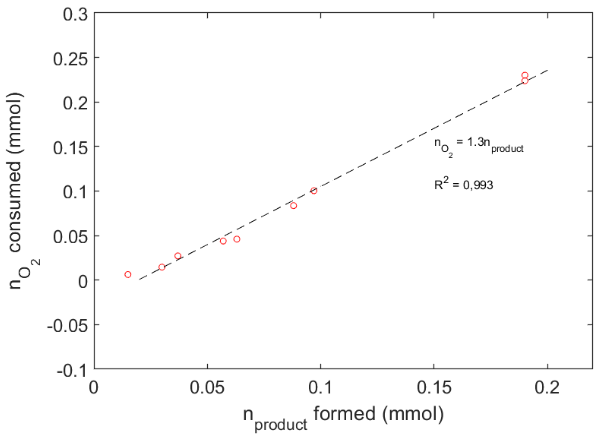

Figure S6. Molar amount of oxygen consumed as a function of product formation.

1.8 Quantum yield determination

Following a published procedure,[1] actinometer solution was prepared by dissolving potassium ferrioxalate (mg) and 280 µL concentrated sulfuric acid in 100 mL deionized water in a volumetric flask. A buffer solution was separately prepared from sodium acetate (mg) and concentrated sulfuric aicd (1 mL) in 100 mL deionized water in a volumetric flask. Finally, 1,10-phenanthroline (200 mg) was dissolved in 100 mL water in a separate volumetric flask.

To measure the light intensity of the light source, 2 mL of the actinometer solution was transferred to a 1x1 cm quartz cuvette and was irradiated for a set time. Care was taken to exclude light exposure of the solution prior and after irradiation. Five different 2 mL portions of the solution were thus irradiated at different times (5, 10, 20, 29 and 60 minutes). After irradiation, each sample was added to a 10 mL volumetric flask containing 2 mL buffer solution and 1 mL 1,10-phenathroline solution. Water was then added to the mark. After resting in room temperature protected from light for 30 minutes, 3 mL of each resulting solution was transferred to 1x1 cm quartz cuvettes and the absorbance at 510 nm was noted and compared to a blank stored protected from light. With the reported molar absorptivity of the iron(II)phenanthroline complex (11 100 L mol^{-1} cm^{-1}) and the difference in absorption between the developed blank and each sample, the molar amount of iron(II) formed during the irradiation was determined according to equation (1).

$$moles\ Fe(II) = \frac{V1*V3*\Delta A_{510\ nm}}{10^3*V2*l*\varepsilon_{510\ nm}} \qquad (1)$$

Where V1 is the volume of the irradiated sample, V2 is the volume of the irradiated sample taken for the determination of amount of Fe(II), V3 is the final volume of the sample measured, l is the path length of light (1 cm), $\Delta A_{510\ nm}$ is the difference in absorption between the irradiated samples and the reference sample kept in dark, and $\varepsilon_{510\ nm}$ is the molar extinction coefficient of the iron(II) phenanthroline complex (11 100 L mol^{-1} cm^{-1}).

The amount of iron(II) was then plotted as a function of irradiation time and from the slope of the fitted data, the photon flux can be calculated according to eq. 2,

$$photon\ flux = \frac{\frac{dx}{dt}}{\phi(450\ nm)*(1-10^{-A(450\ nm)})} \qquad (2)$$

where dx/dt is the rate of formation of iron(II), $\phi(450\ nm)$ is the quantum yield for the ferrioxalate actinometer at 450 nm excitation (0.9) and A(450 nm) is the absorbance of the ferrioxalate actinometer at the irradiation wavelength (0.228). Consequently, the photon flux was determined to be 1*10^{-9} moles of photons s^{-1}.

Reactions prepared protected from light. DBE, Maleimide and dodecane mixed in a vial and separately the amine and dioxane in another vial. When in dark room, the amine solution was added to the solids and when solids were dissolved 3 mL was transferred to a 1x1 cm quartz vial and irradiated with 450 nm (5 nm slit) for different times. The molar amount of product formed was determined using GC-FID with n-dodecane as internal standard and was plotted as a function of time. The quantum yield (moles of product formed per moles of absorbed photons) was then calculated according to equation 3:

$$\phi = \frac{moles\ product\ formed/time}{photon\ flux*(1-10^{-A(reaction\ mixture\ at\ 450\ nm)})} \qquad (3)$$

Accordingly, a quantum yield of 0.07 could be estimated.

2.1 References

(1) Kandukuri, S. R.; Bahamonde, A.; Chatterjee, I.; Jurberg, I. D.; Escudero-Adán, E. C.; Melchiorre, P. X-Ray Characterization of an Electron Donor–Acceptor Complex That Drives the Photochemical Alkylation of Indoles. *Angew. Chemie Int. Ed.* **2015**, *54* (5), 1485–1489. https://doi.org/10.1002/anie.201409529.

(2) Hansch, C.; Leo, A.; Taft, R. W. A Survey of Hammett Substituent Constants and Resonance and Field Parameters. *Chem. Rev.* **1991**, *91* (2), 165–195. https://doi.org/10.1021/cr00002a004.

(3) Li, J.; Bao, W.; Zhang, Y.; Rao, Y. Cercosporin-Photocatalyzed Sp3 (C–H) Activation for the Synthesis of Pyrrolo[3{,}4-c]Quinolones. *Org. Biomol. Chem.* **2019**, *17* (40), 8958–8962. https://doi.org/10.1039/C9OB01946D.

(4) Xu, W.-L.; Tang, L.; Ge, C.-Y.; Chen, J.; Zhou, L. Synthesis of Tetrahydroisoindolinones via a Metal-Free Dehydrogenative Diels-Alder Reaction. *Adv. Synth. \& Catal.* **2019**, *361* (10), 2268–2273. https://doi.org/https://doi.org/10.1002/adsc.201801436.

(5) Jiang, S.; Tala, S. R.; Lu, H.; Zou, P.; Avan, I.; Ibrahim, T. S.; Abo-Dya, N. E.; Abdelmajeid, A.; Debnath, A. K.; Katritzky, A. R. Design, Synthesis, and Biological Activity of a Novel Series of 2,5-Disubstituted Furans/Pyrroles as HIV-1 Fusion Inhibitors Targeting Gp41. *Bioorg. Med. Chem. Lett.* **2011**, *21* (22), 6895–6898. https://doi.org/https://doi.org/10.1016/j.bmcl.2011.08.081.

(6) Lu, C.-D.; Chen, Z.-Y.; Liu, H.; Hu, W.-H.; Mi, A.-Q.; Doyle, M. P. A Facile Three-Component One-Pot Synthesis of Structurally Constrained Tetrahydrofurans That Are t-RNA Synthetase Inhibitor Analogues. *J. Org. Chem.* **2004**, *69* (14), 4856–4859. https://doi.org/10.1021/jo0497508.

(7) Matuszak, N.; Muccioli, G. G.; Labar, G.; Lambert, D. M. Synthesis and in Vitro Evaluation of N-Substituted Maleimide Derivatives as Selective Monoglyceride Lipase Inhibitors. *J. Med. Chem.* **2009**, *52* (23), 7410–7420. https://doi.org/10.1021/jm900461w.

(8) Samgina, T. Y.; Gorshkov, V. A.; Vorontsov, E. A.; Bagrov, V. V; Nifant'ev, I. E.; Lebedev, A. T. New Cysteine-Modifying Reagents: Efficiency of Derivatization and Influence on the Signals of the Protonated Molecules of Disulfide-Containing Peptides in Matrix-Assisted Laser Desorption/Ionization Mass Spectrometry. *J. Anal. Chem.* **2010**, *65* (13), 1320–1327. https://doi.org/10.1134/S1061934810130034.

(9) Salewska, N.; Boros-Majewska, J.; Łącka, I.; Chylińska, K.; Sabisz, M.; Milewski, S.; Milewska, M. J. Chemical Reactivity and Antimicrobial Activity of N-Substituted Maleimides. *J. Enzyme Inhib. Med. Chem.* **2012**, *27* (1), 117–124. https://doi.org/10.3109/14756366.2011.580455.

(10) Hsu, C.-W.; Sundén, H. α-Aminoalkyl Radical Addition to Maleimides via Electron Donor–Acceptor Complexes. *Org. Lett.* **2018**, *20* (7), 2051–2054. https://doi.org/10.1021/acs.orglett.8b00597.

(11) Tang, J.; Grampp, G.; Liu, Y.; Wang, B.-X.; Tao, F.-F.; Wang, L.-J.; Liang, X.-Z.; Xiao, H.-Q.; Shen, Y.-M. Visible Light Mediated Cyclization of Tertiary Anilines with Maleimides Using Nickel(II) Oxide Surface-Modified Titanium Dioxide Catalyst. *J. Org. Chem.* **2015**, *80* (5), 2724–2732. https://doi.org/10.1021/jo502901h.

(12) Yang, X.-L.; Guo, J.-D.; Lei, T.; Chen, B.; Tung, C.-H.; Wu, L.-Z. Oxidative Cyclization Synthesis of Tetrahydroquinolines and Reductive Hydrogenation of Maleimides under Redox-Neutral Conditions. *Org. Lett.* **2018**, *20* (10), 2916–2920. https://doi.org/10.1021/acs.orglett.8b00977.

(13) Nicholls, T. P.; Constable, G. E.; Robertson, J. C.; Gardiner, M. G.; Bissember, A. C. Bronsted Acid Cocatalysis in Copper(I)-Photocatalyzed α-Amino C-H Bond Functionalization. *ACS Catal.* **2016**, *6* (1), 451–457. https://doi.org/10.1021/acscatal.5b02014.

(14) Huang, P.; Wang, P.; Wang, S.; Tang, S.; Lei, A. Electrochemical Oxidative [4 + 2] Annulation of Tertiary Anilines and Alkenes for the Synthesis of Tetrahydroquinolines. *Green Chem.* **2018**, *20* (21), 4870–4874. https://doi.org/10.1039/c8gc02463d.

(15) Sharma, K.; Das, B.; Gogoi, P. Synthesis of Pyrrolo[3,4-c]Quinoline-1,3-Diones: A Sequential Oxidative Annulation Followed by Dehydrogenation and N-Demethylation Strategy. *New J. Chem.* **2018**, *42* (23), 18894–18905. https://doi.org/10.1039/c8nj04443k.

(16) Perumal, G.; Kandasamy, M.; Ganesan, B.; Govindan, K.; Sathya, H.; Hung, M.-Y.; Chandru Senadi, G.; Wu, Y.-C.; Lin, W.-Y. Visible Light-Induced N-Methyl Activation of Unsymmetric Tertiary Amines. *Tetrahedron* **2021**, *80*, 131891. https://doi.org/https://doi.org/10.1016/j.tet.2020.131891.

(17) Song, Z.; Antonchick, A. P. Catching α-Aminoalkyl Radicals: Cyclization between Tertiary Alkylanilines and Alkenes. *Tetrahedron* **2016**, *72* (48), 7715–7721. https://doi.org/https://doi.org/10.1016/j.tet.2016.04.052.

2.1 NMR spectra

Starting materials

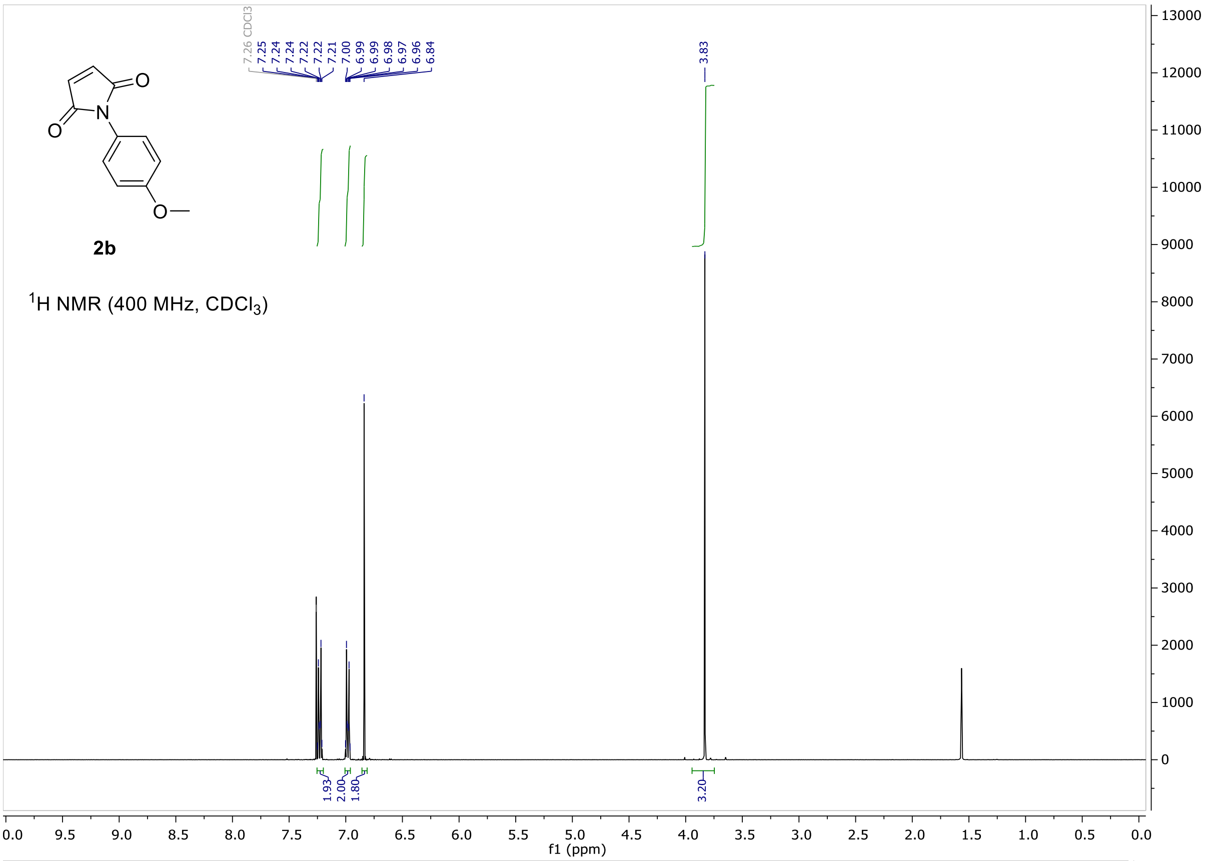

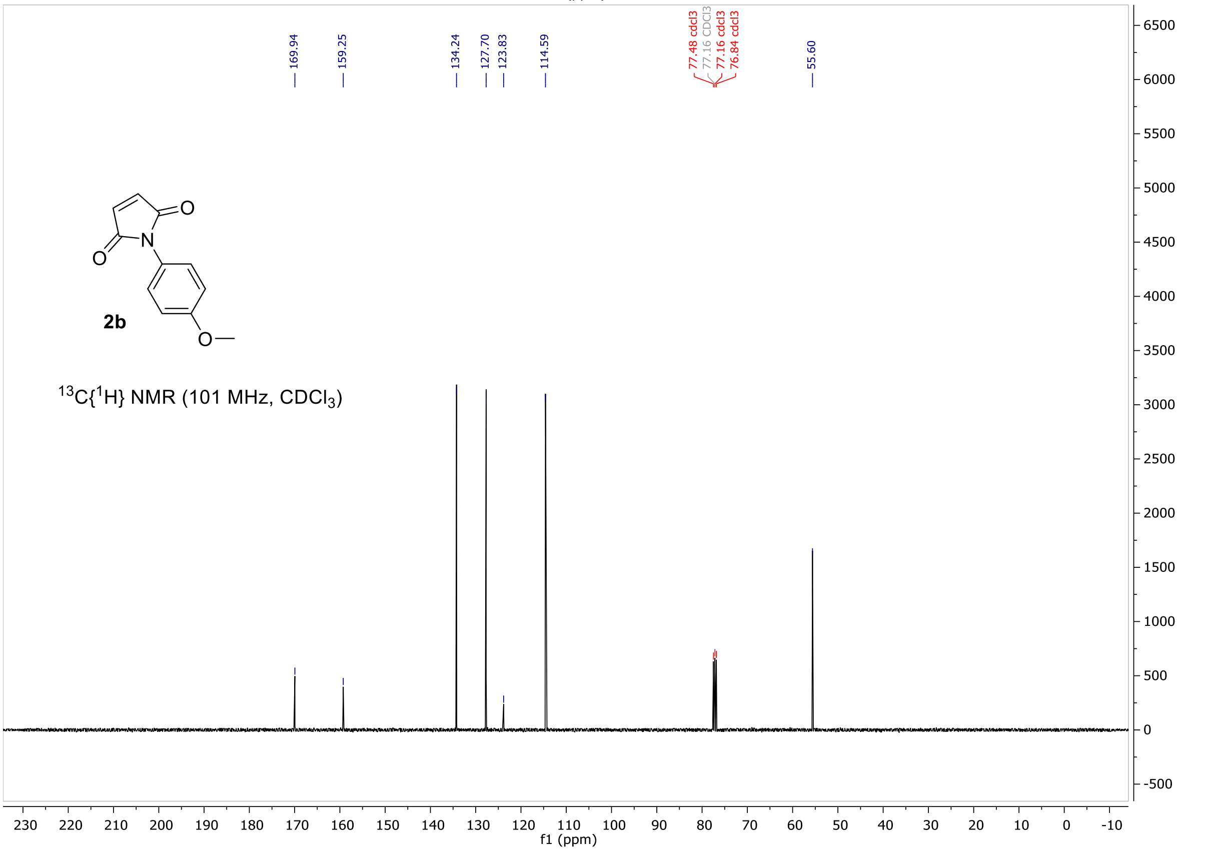

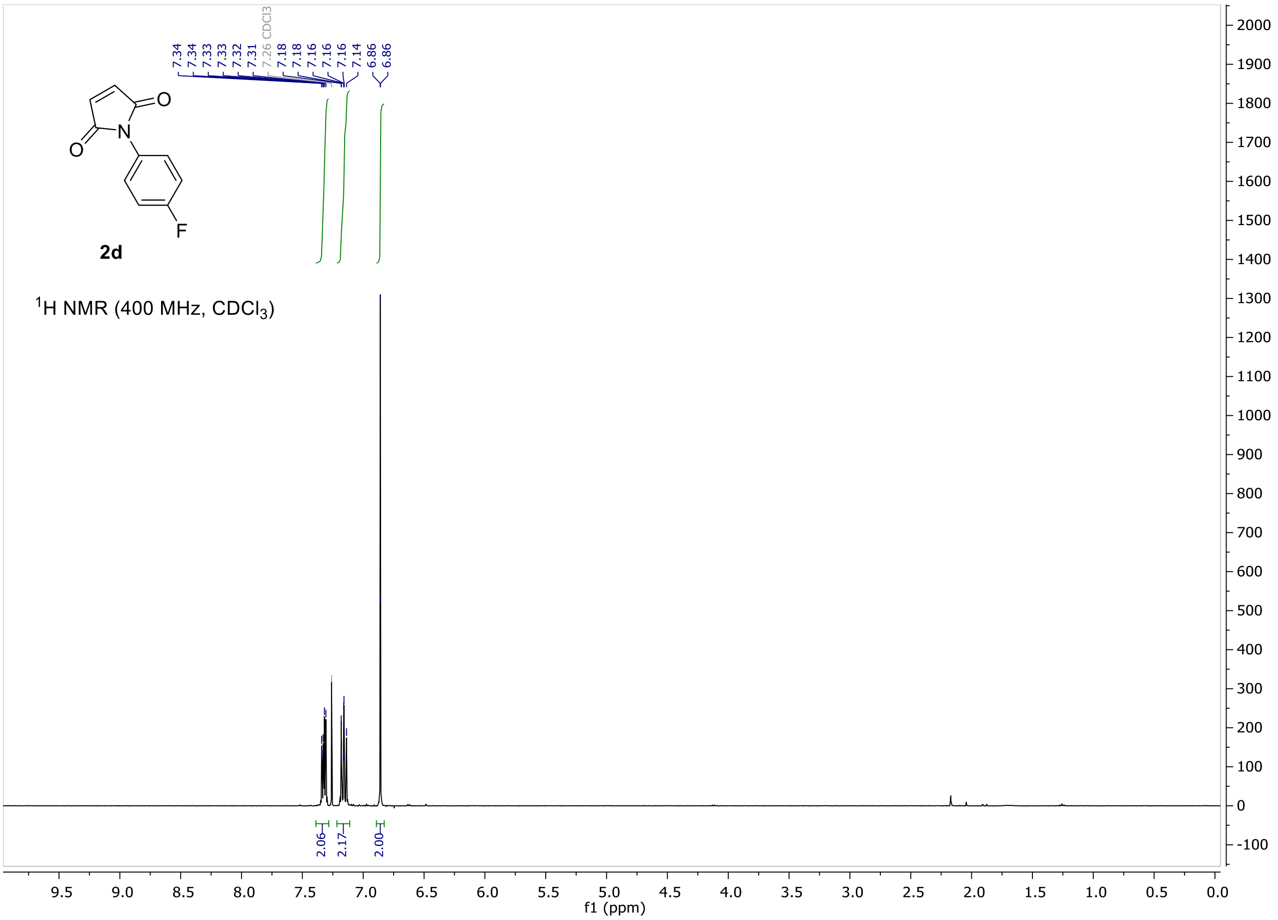
2d
^{1}H NMR (400 MHz, CDCl$_3$)
7.34 7.34 7.33 7.33 7.32 7.31 7.26 CDCl3 7.18 7.18 7.16 7.16 7.16 7.14 6.86 6.86
2.06 2.17 2.00
f1 (ppm)

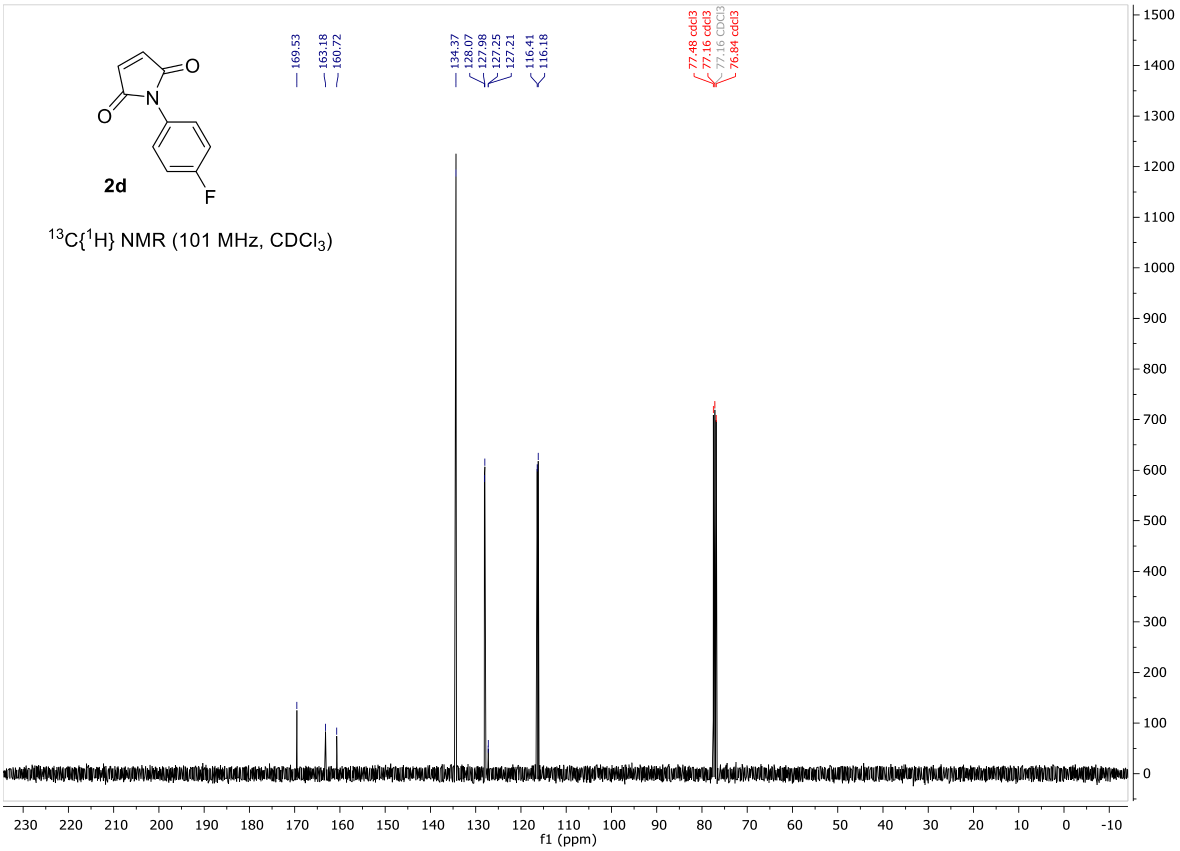
2d
^{13}C{^{1}H} NMR (101 MHz, CDCl$_3$)
169.53 163.18 160.72 134.37 128.07 127.98 127.25 127.21 116.41 116.18
77.48 cdcl3 77.16 cdcl3 77.16 CDCl3 76.84 cdcl3
f1 (ppm)

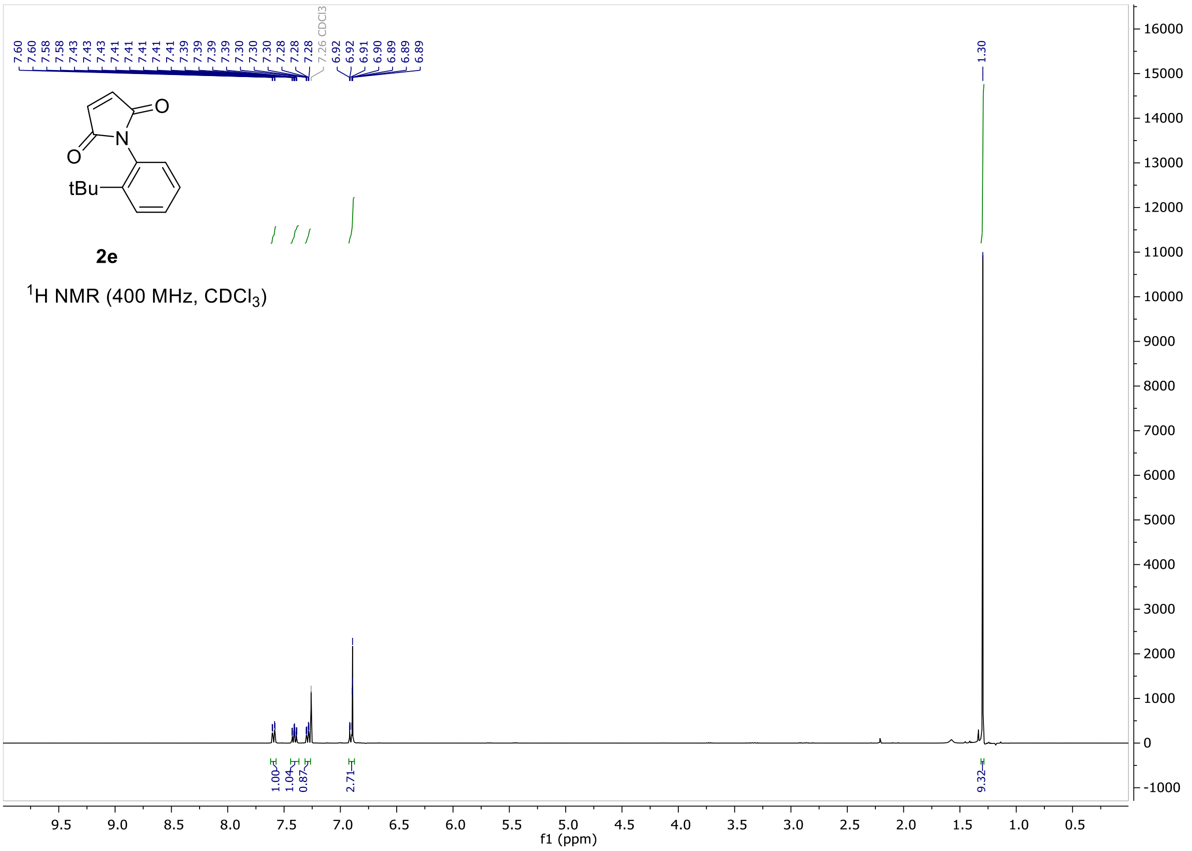

7.60 7.60 7.58 7.58 7.43 7.43 7.43 7.41 7.41 7.41 7.41 7.39 7.39 7.39 7.39 7.30 7.30 7.30 7.28 7.28 7.28
7.26 CDCl3
6.92 6.92 6.91 6.90 6.89 6.89 6.89
1.30
O
N
O
tBu
2e
^{1}H NMR (400 MHz, $CDCl_3$)
1.00
1.04
0.87
2.71
9.32
f1 (ppm)

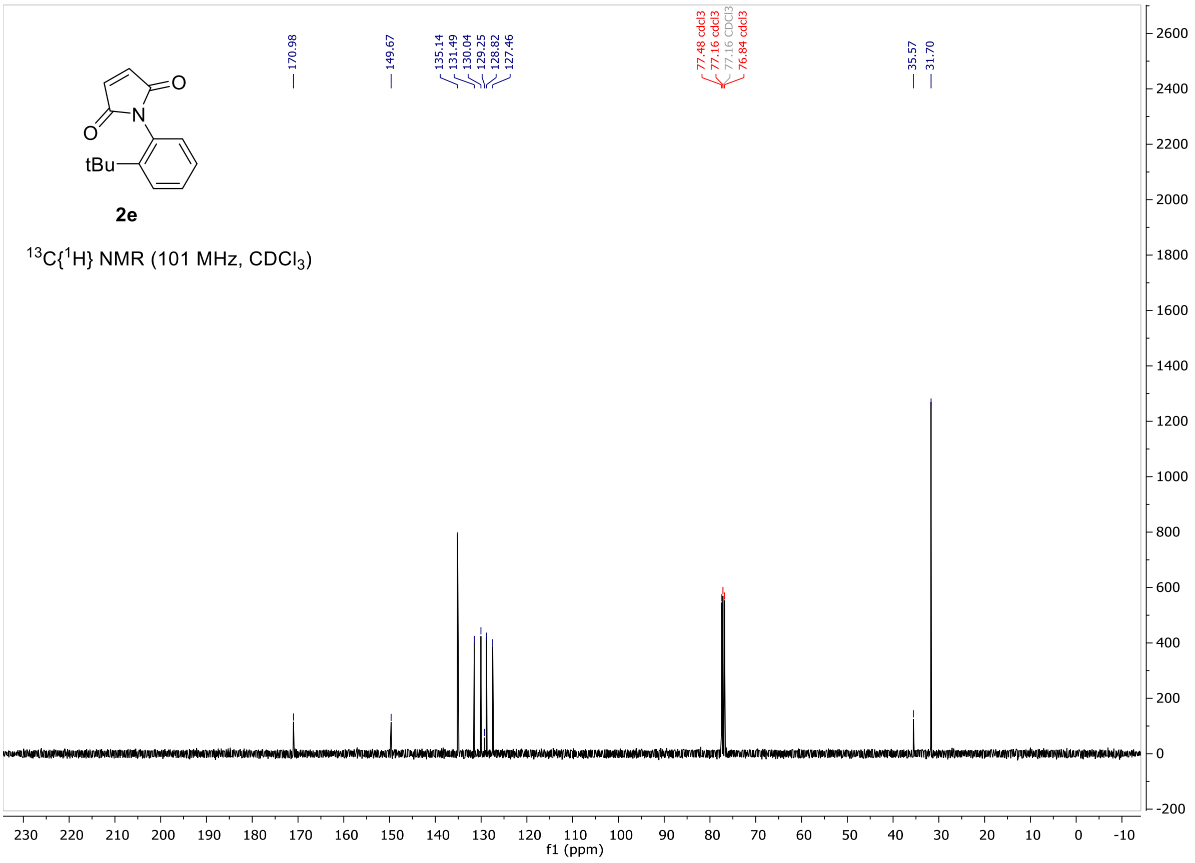

170.98
149.67
135.14 131.49 130.04 129.25 128.82 127.46
77.48 cdcl3
77.16 cdcl3
77.16 CDCl3
76.84 cdcl3
35.57
31.70
O
N
O
tBu
2e
^{13}C{^{1}H} NMR (101 MHz, $CDCl_3$)
f1 (ppm)

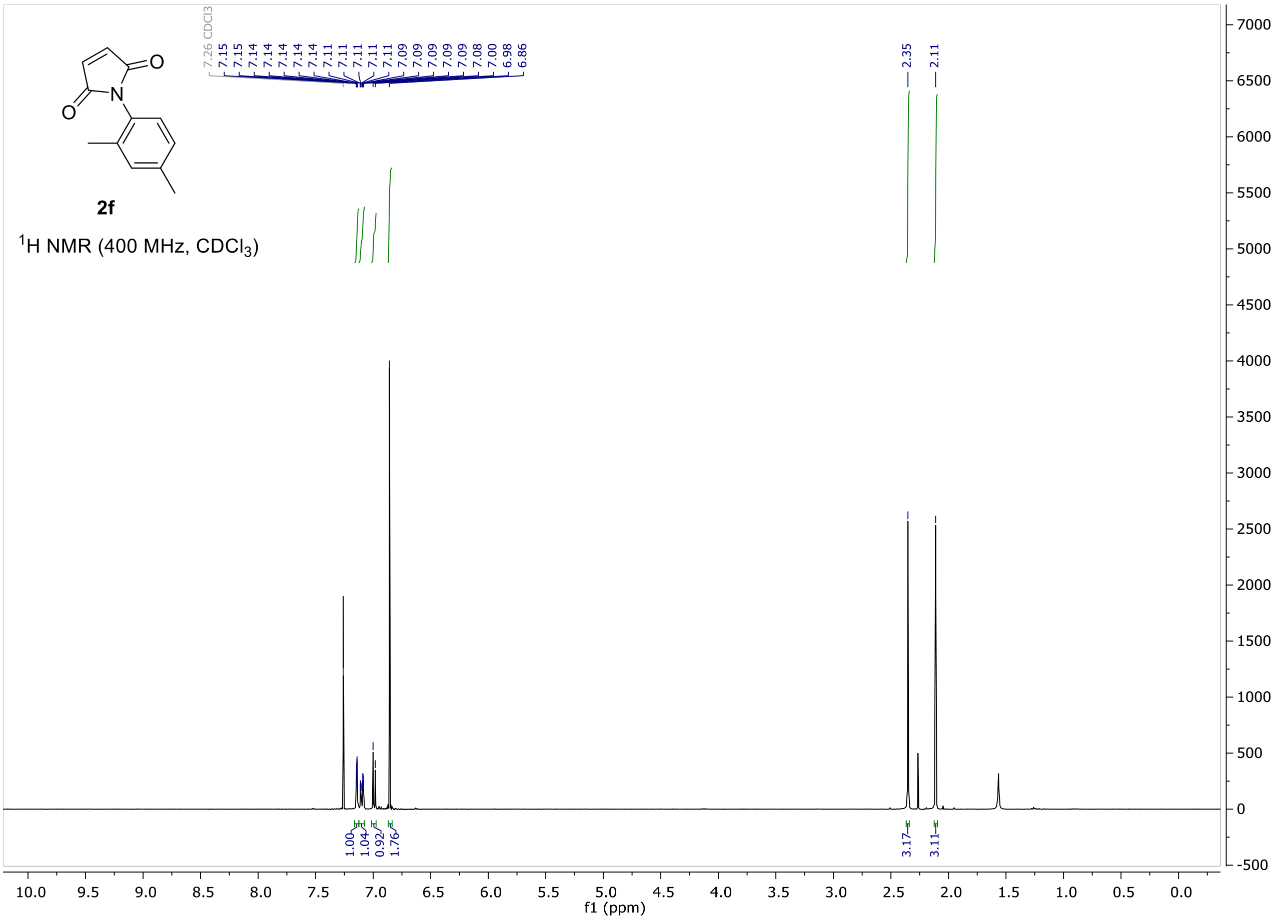

2f
^{1}H NMR (400 MHz, CDCl$_3$)
7.26 CDCl3
7.15
7.15
7.14
7.14
7.14
7.14
7.11
7.11
7.11
7.11
7.11
7.09
7.09
7.09
7.09
7.08
7.00
6.98
6.86
2.35
2.11
1.00
1.04
0.92
1.76
3.17
3.11
f1 (ppm)

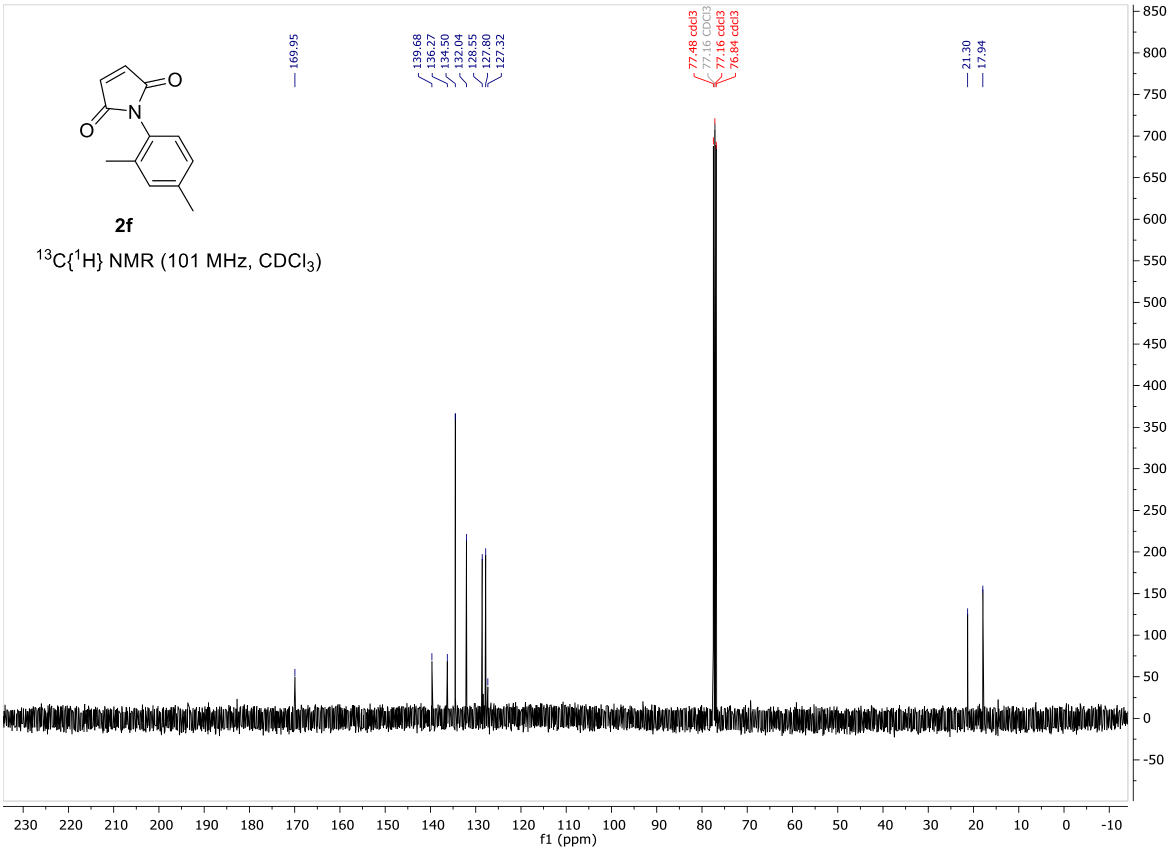

2f
^{13}C{^{1}H} NMR (101 MHz, CDCl$_3$)
169.95
139.68
136.27
134.50
132.04
128.55
127.80
127.32
77.48 cdcl3
77.16 CDCl3
77.16 cdcl3
76.84 cdcl3
21.30
17.94
f1 (ppm)

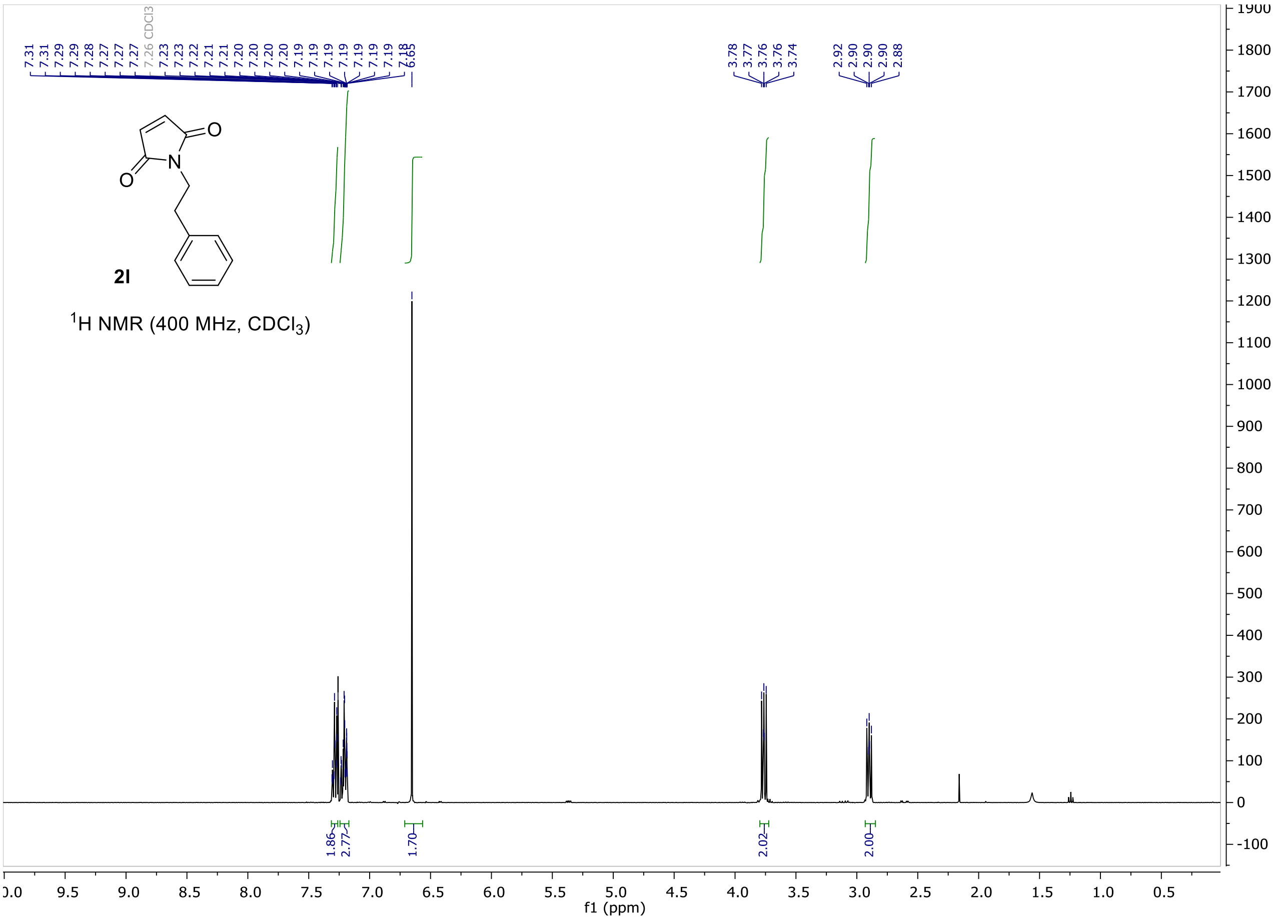
2l
^{1}H NMR (400 MHz, CDCl$_3$)
f1 (ppm)

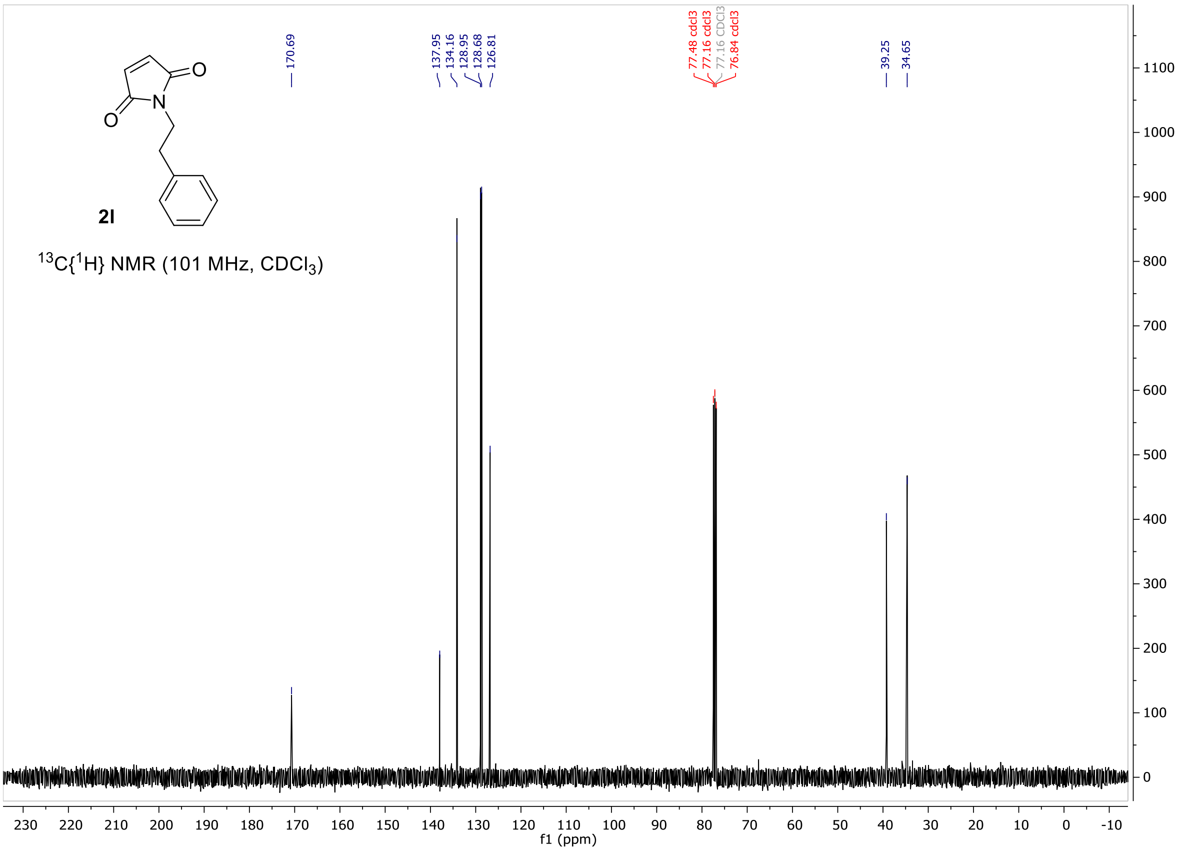
2l
^{13}C{^{1}H} NMR (101 MHz, CDCl$_3$)
f1 (ppm)

Tetrahydriquionoline products

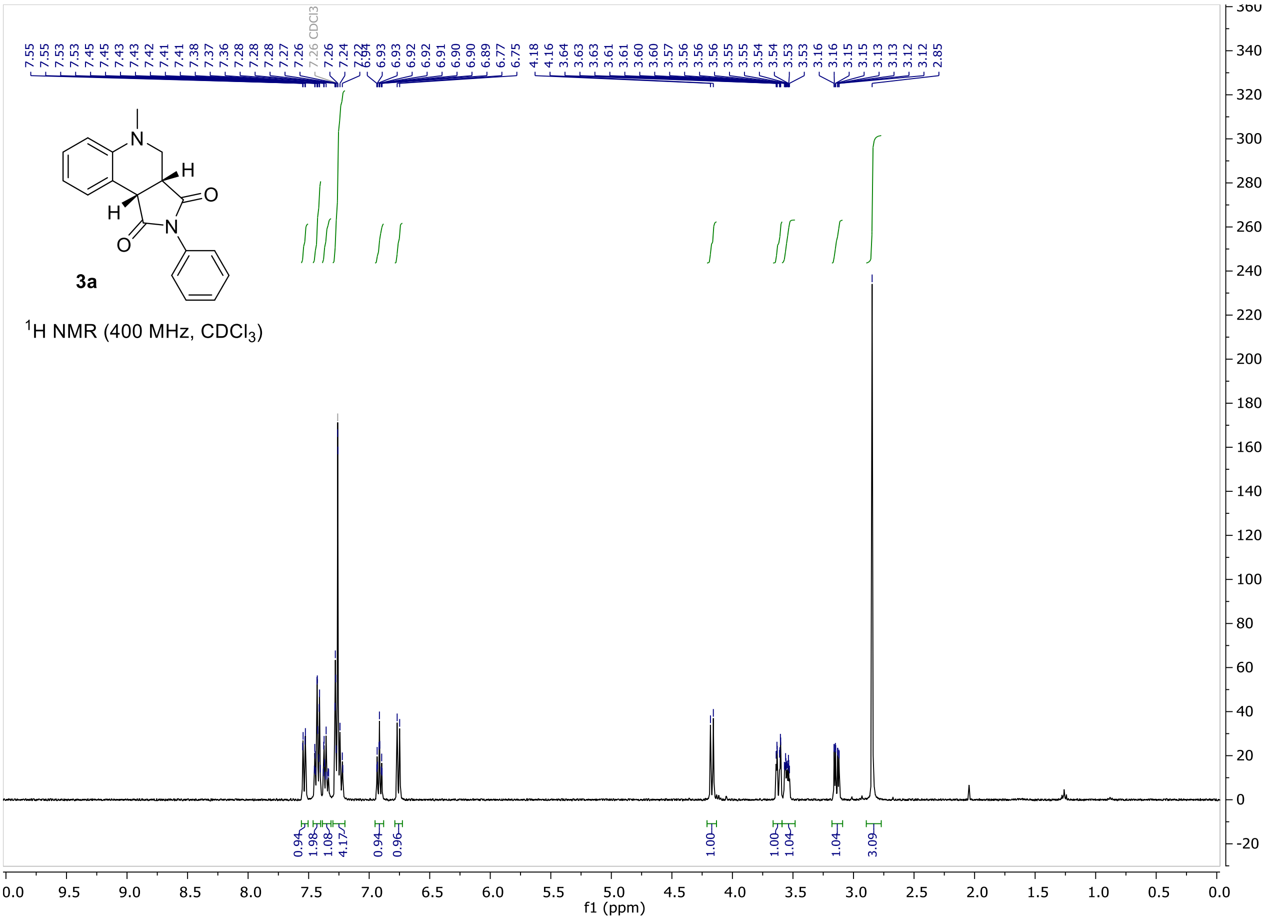

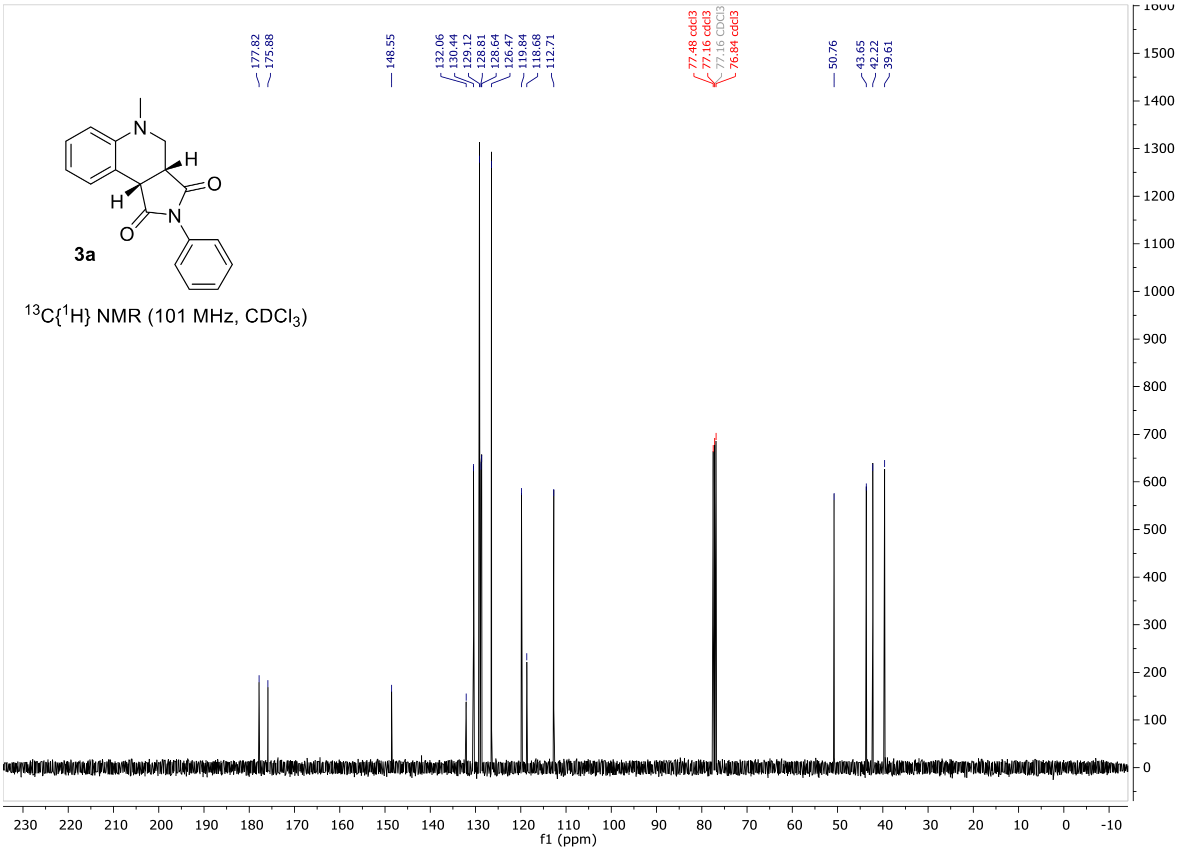

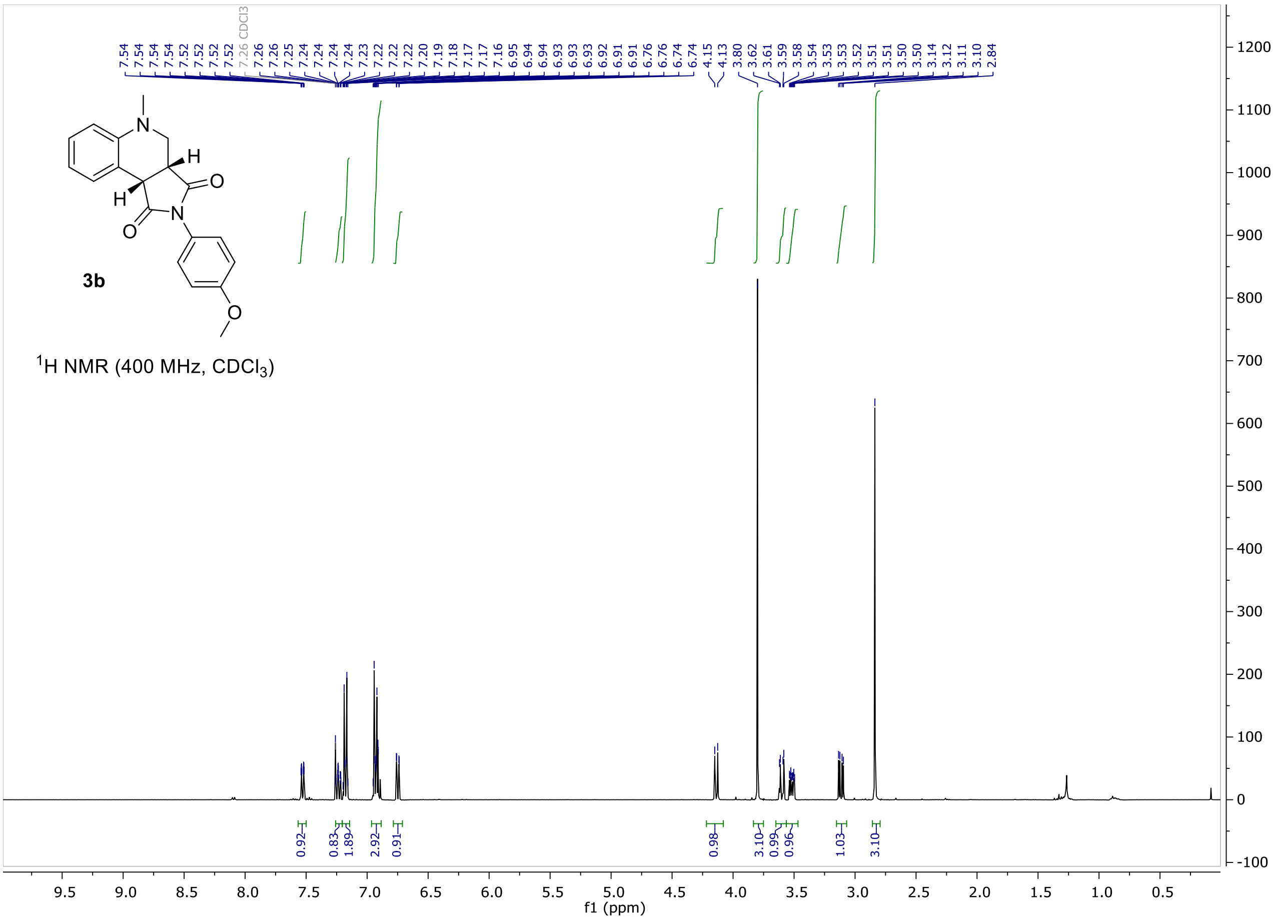
3b
^{1}H NMR (400 MHz, CDCl$_3$)
f1 (ppm)

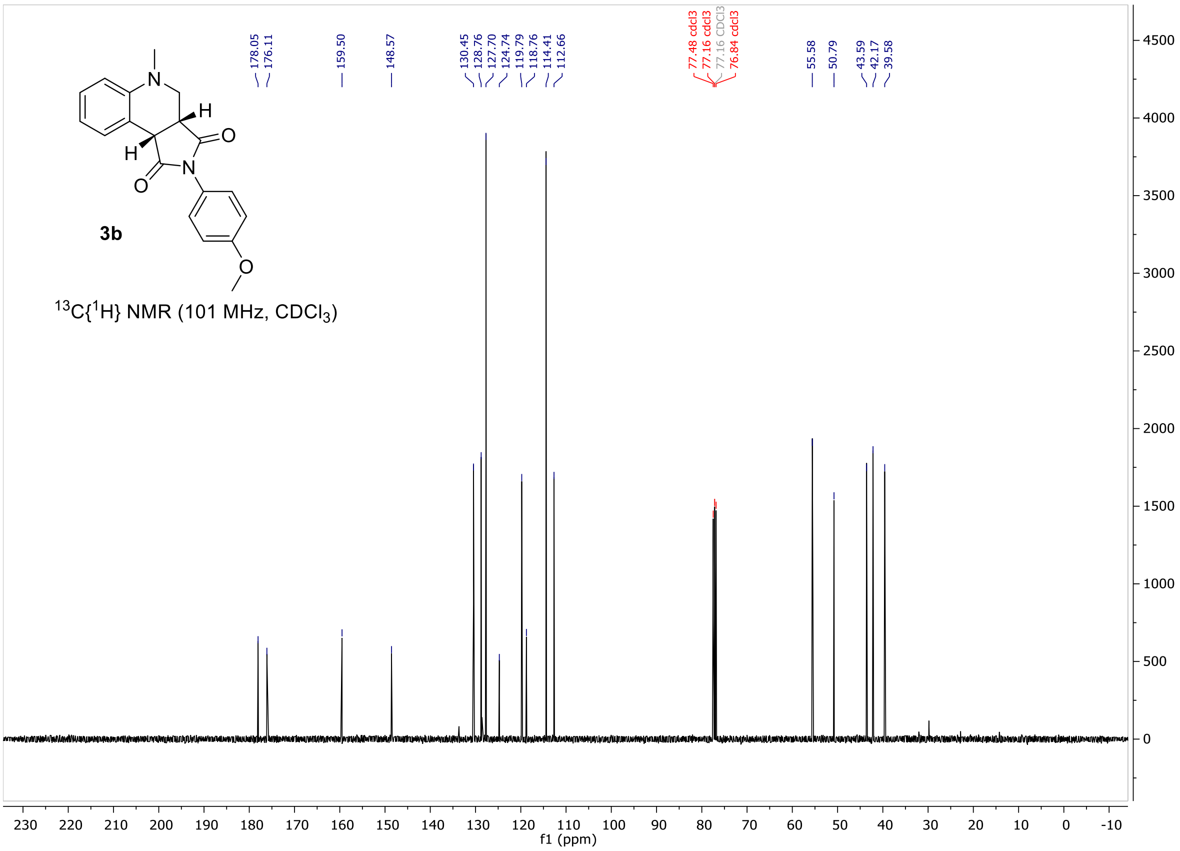
3b
^{13}C{^{1}H} NMR (101 MHz, CDCl$_3$)
f1 (ppm)

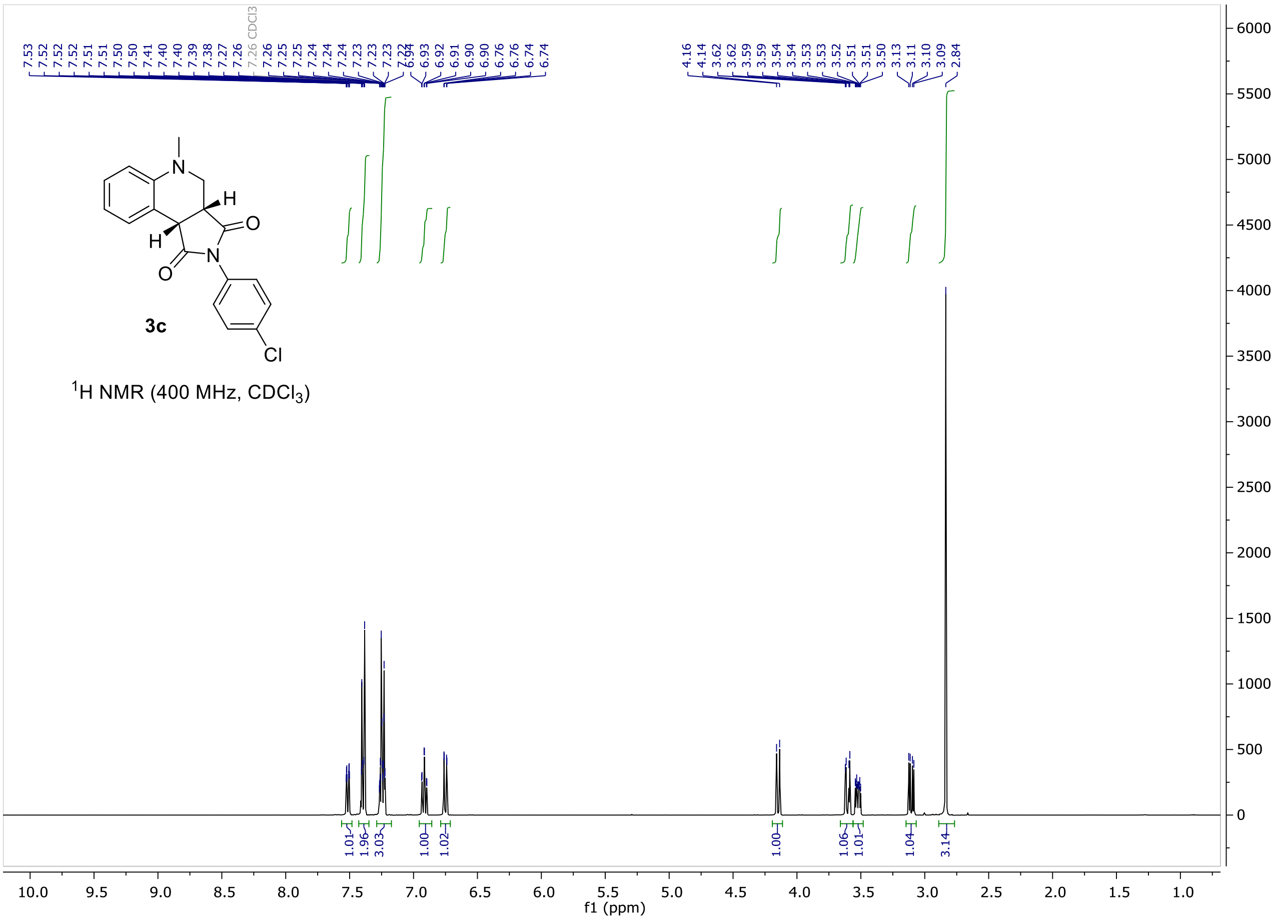
3c
^{1}H NMR (400 MHz, CDCl$_3$)
f1 (ppm)

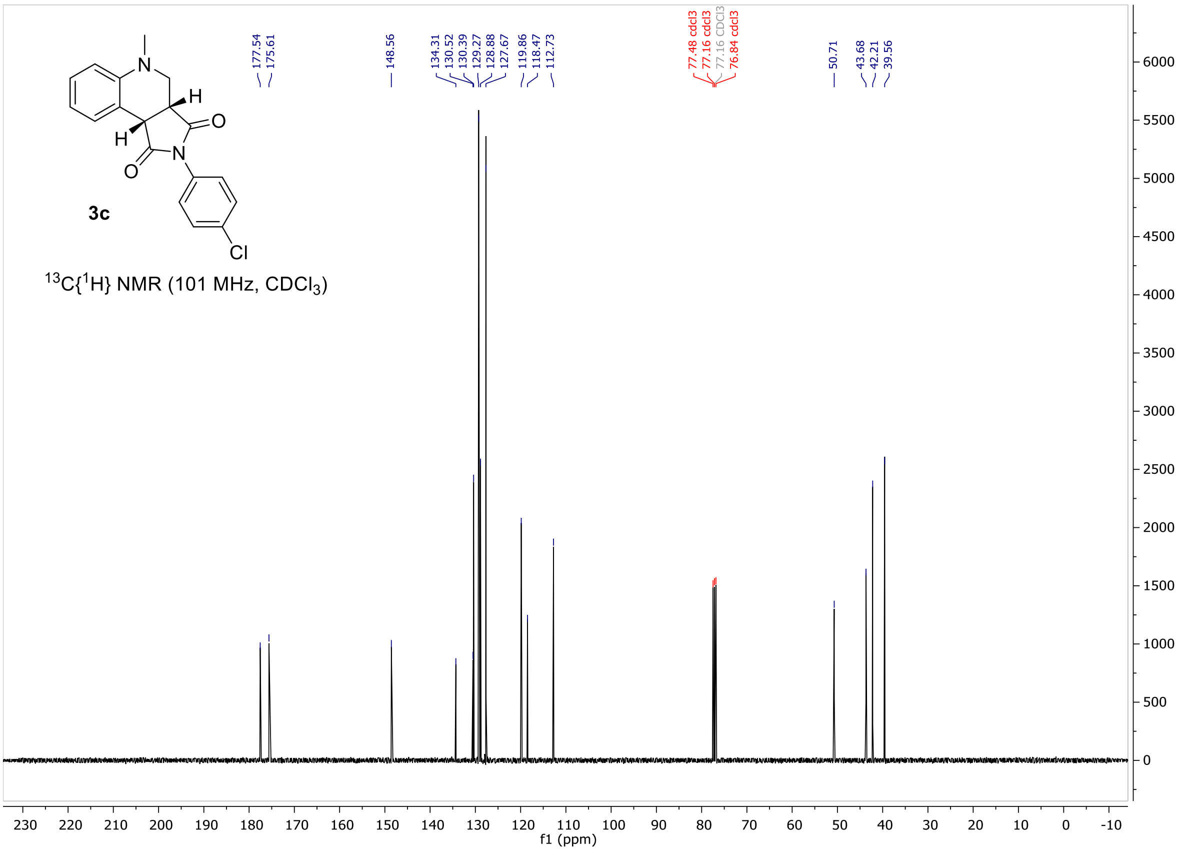
3c
^{13}C{^{1}H} NMR (101 MHz, CDCl$_3$)
177.54
175.61
148.56
134.31
130.52
130.39
129.27
128.88
127.67
119.86
118.47
112.73
77.48 cdcl3
77.16 cdcl3
77.16 CDCl3
76.84 cdcl3
50.71
43.68
42.21
39.56
f1 (ppm)

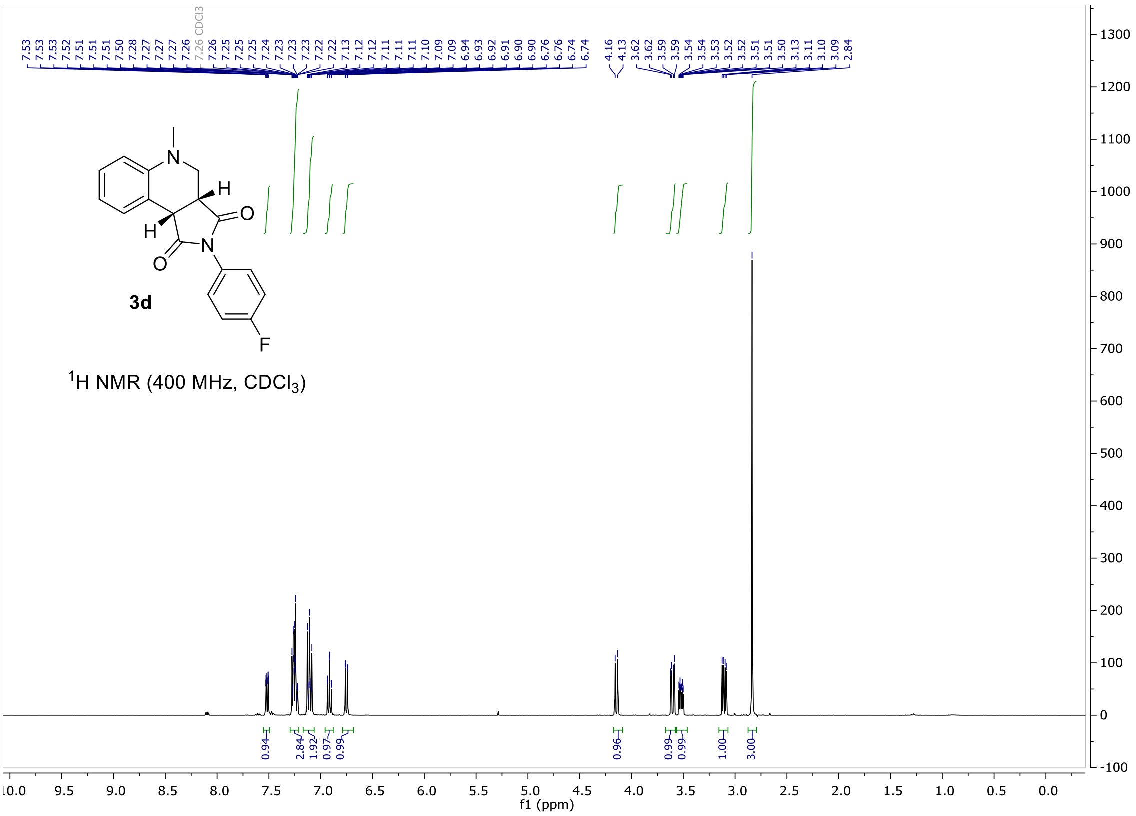

3d
^{1}H NMR (400 MHz, CDCl$_3$)
f1 (ppm)

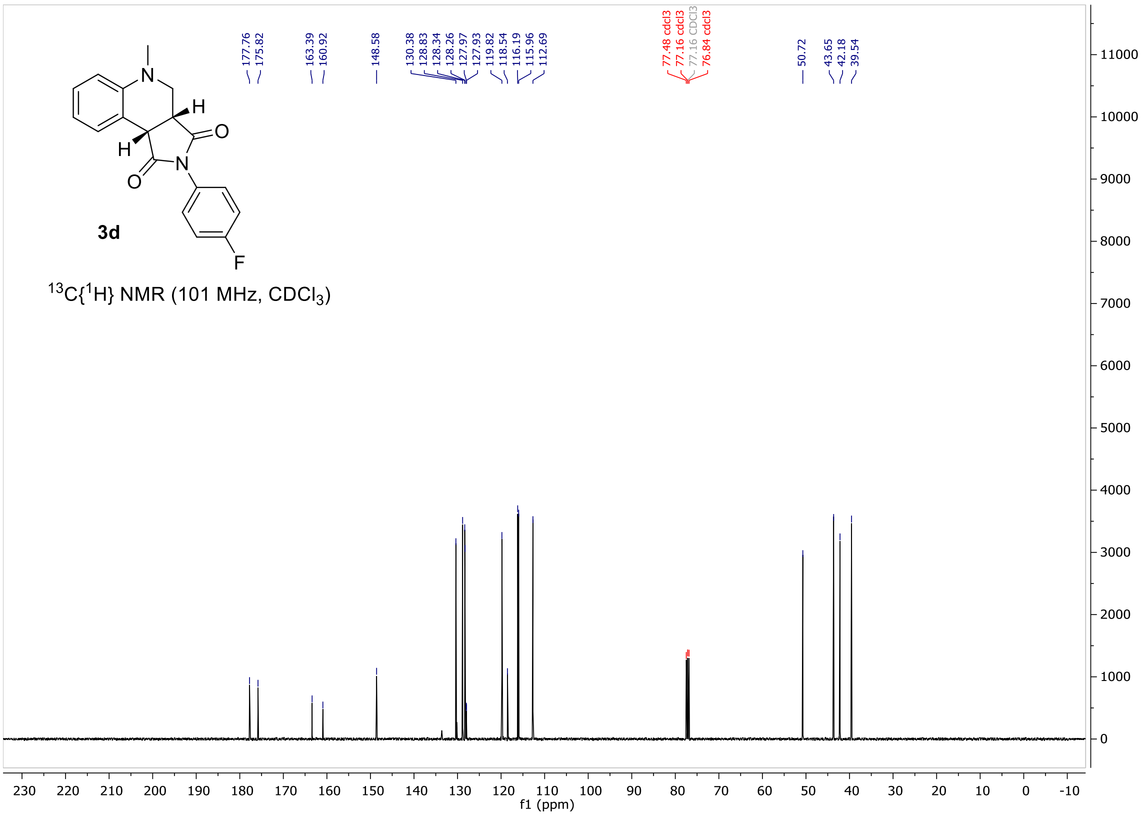

3d
^{13}C{^{1}H} NMR (101 MHz, CDCl$_3$)
177.76
175.82
163.39
160.92
148.58
130.38
128.83
128.34
128.26
127.97
127.93
119.82
118.54
116.19
115.96
112.69
77.48 cdcl3
77.16 cdcl3
77.16 CDCl3
76.84 cdcl3
50.72
43.65
42.18
39.54
f1 (ppm)

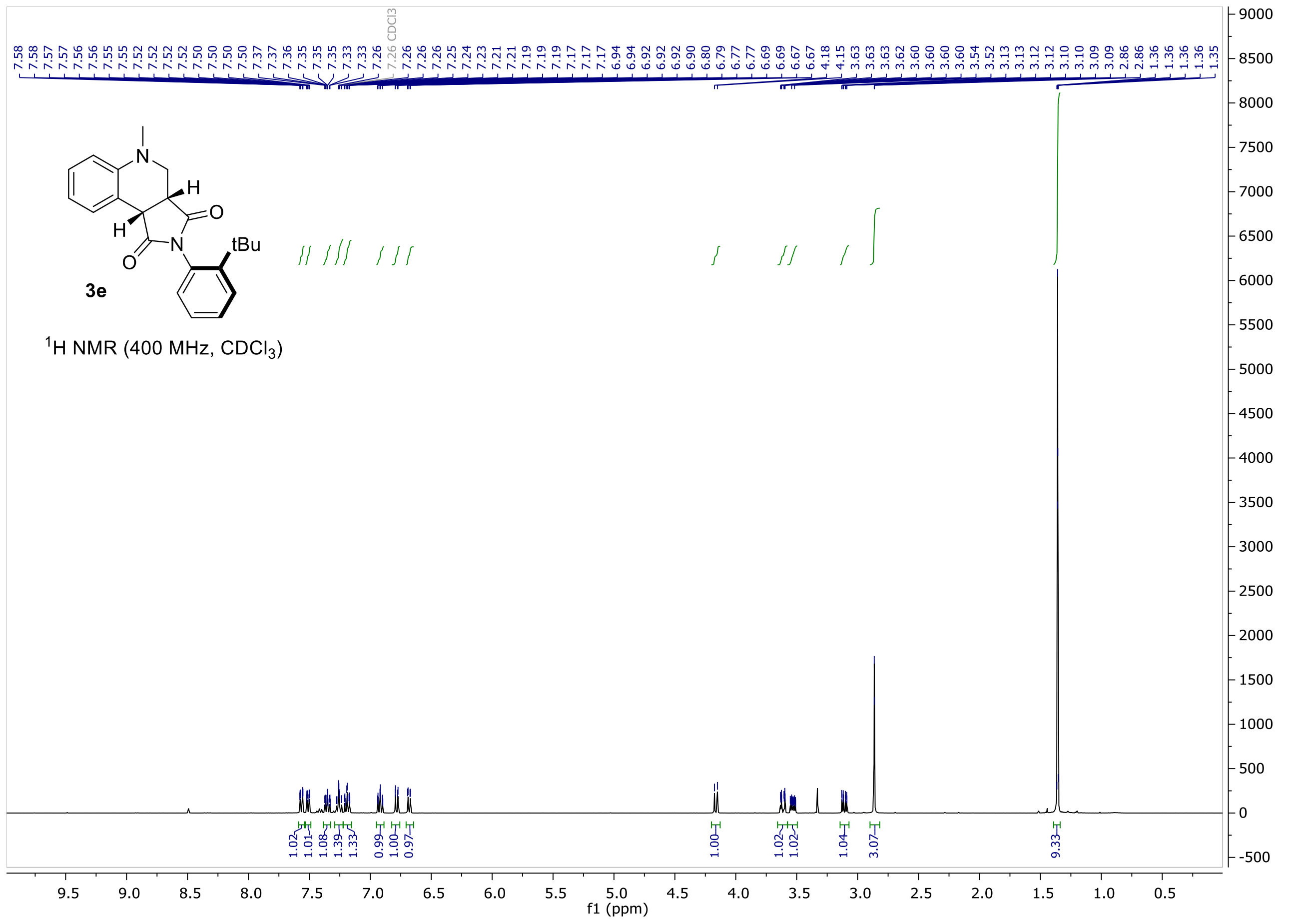
3e
^{1}H NMR (400 MHz, CDCl$_3$)
7.58 7.58 7.57 7.56 7.56 7.55 7.55 7.52 7.52 7.52 7.52 7.50 7.50 7.50 7.50 7.37 7.37 7.36 7.35 7.35 7.35 7.33 7.33 7.26 7.26 CDCl3 7.26 7.26 7.25 7.24 7.23 7.21 7.21 7.19 7.19 7.19 7.17 7.17 6.94 6.94 6.92 6.92 6.92 6.90 6.80 6.79 6.77 6.77 6.69 6.69 6.67 6.67 4.18 4.15 3.63 3.63 3.63 3.62 3.60 3.60 3.60 3.54 3.52 3.13 3.13 3.12 3.12 3.10 3.10 3.09 2.86 1.36 1.36 1.36 1.36 1.35
1.02 1.01 1.08 1.39 1.33 0.99 1.00 0.97 1.00 1.02 1.02 1.04 3.07 9.33
f1 (ppm)

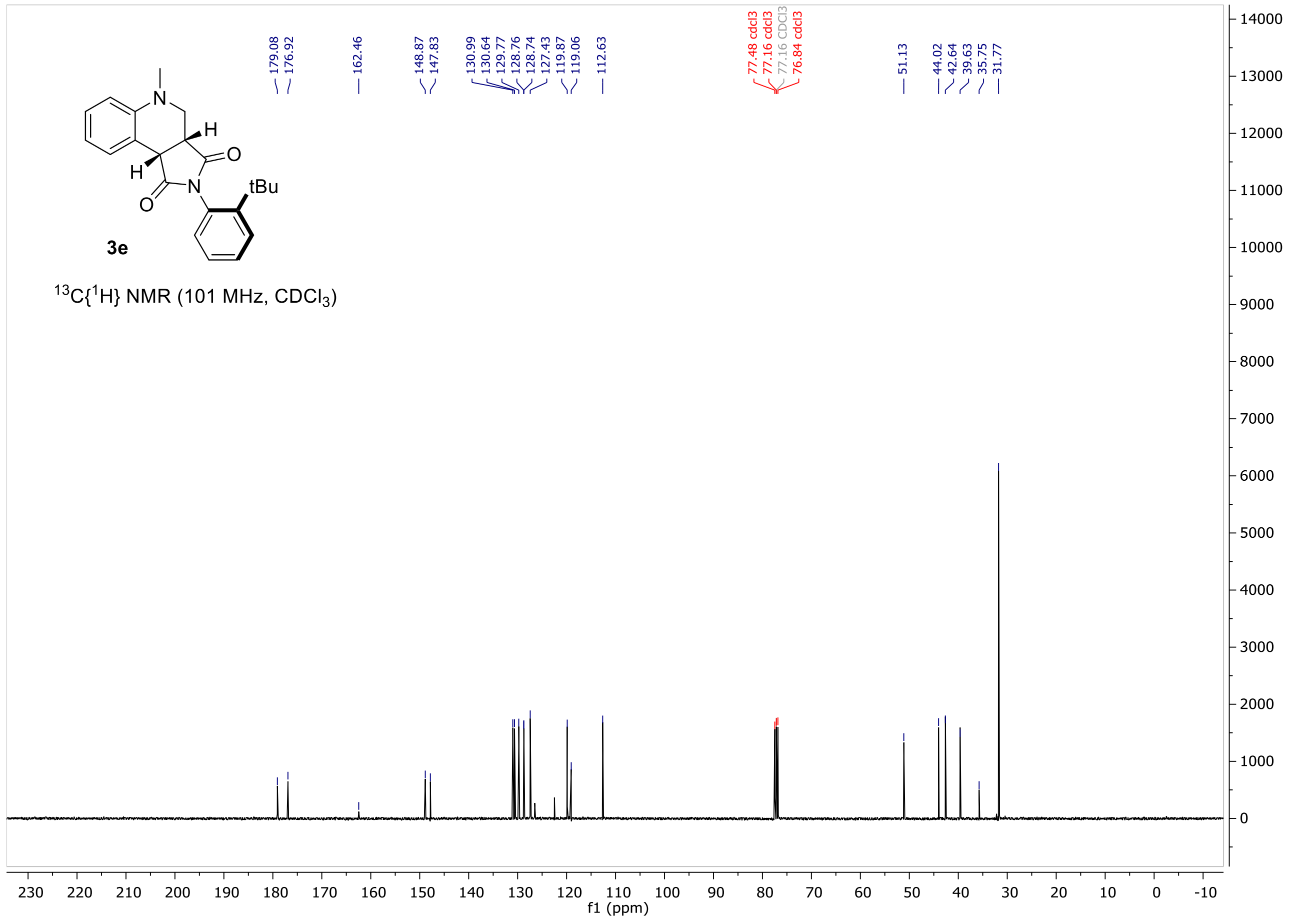
3e
^{13}C{^{1}H} NMR (101 MHz, CDCl$_3$)
179.08 176.92 162.46 148.87 147.83 130.99 130.64 129.77 128.76 128.74 127.43 119.87 119.06 112.63
77.48 cdcl3 77.16 cdcl3 77.16 CDCl3 76.84 cdcl3
51.13 44.02 42.64 39.63 35.75 31.77
f1 (ppm)

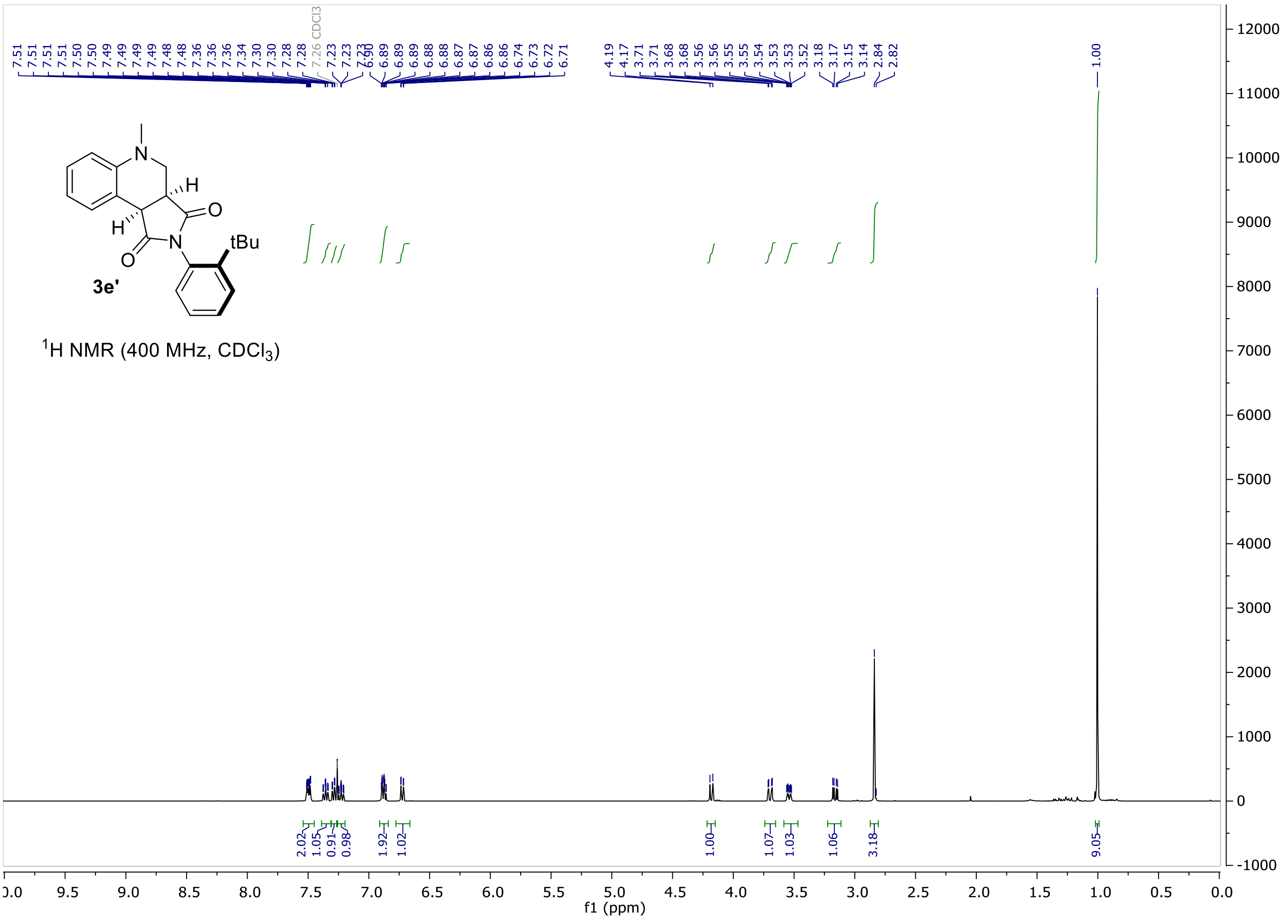

3e'

^{1}H NMR (400 MHz, $CDCl_3$)

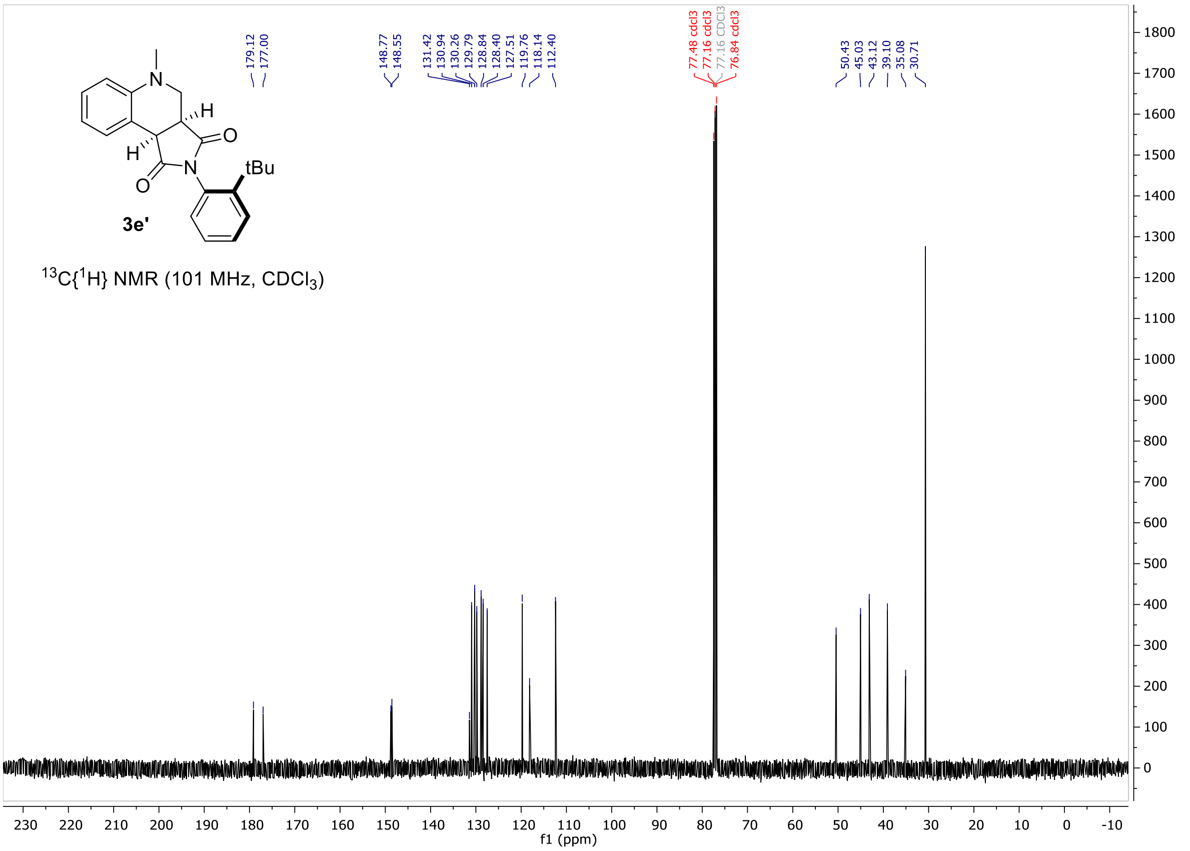

3e'

$^{13}C\{^{1}H\}$ NMR (101 MHz, $CDCl_3$)

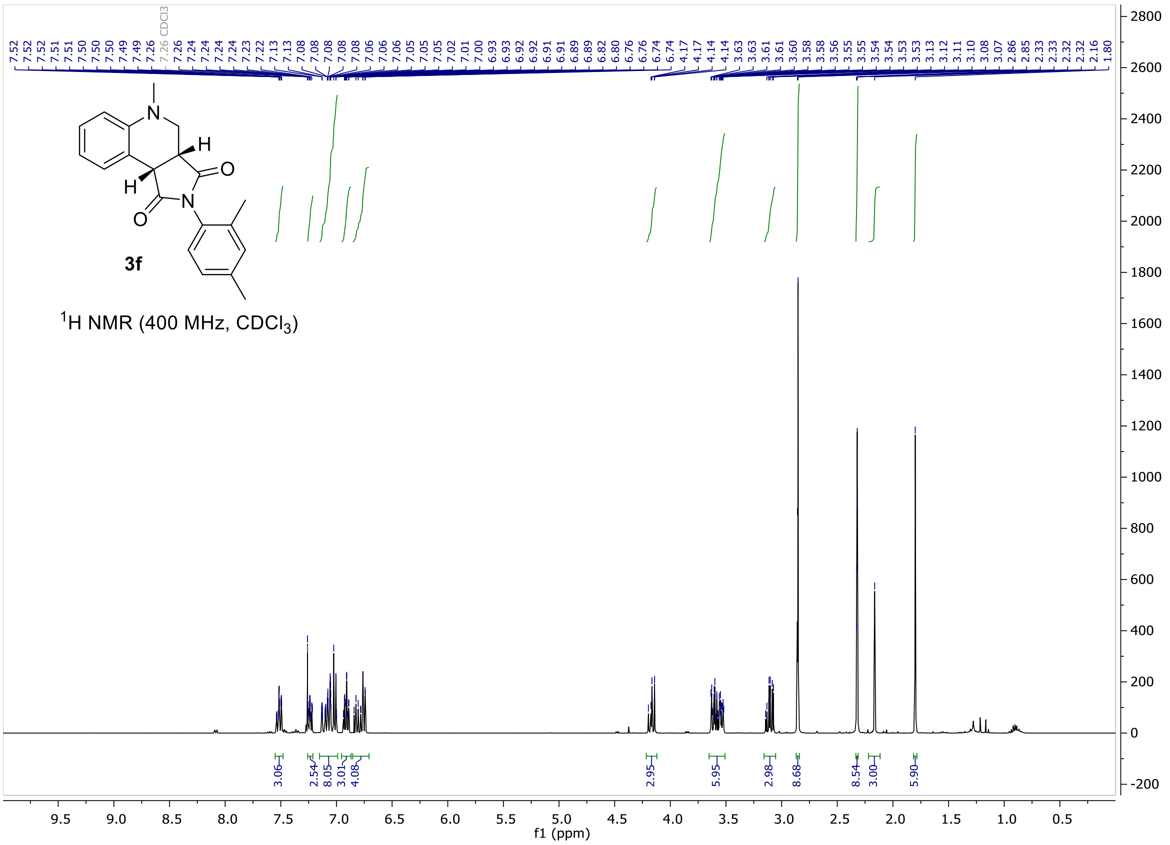

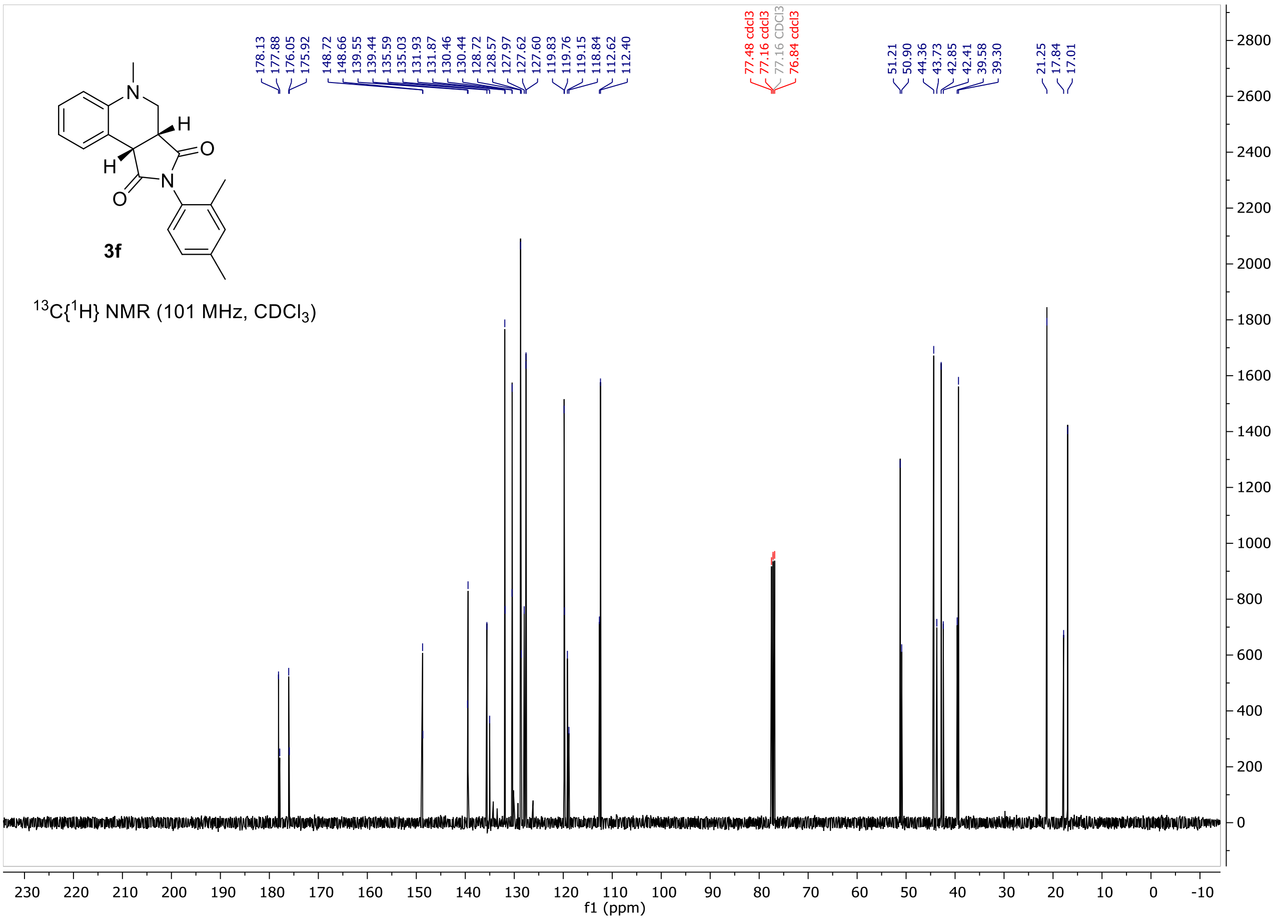

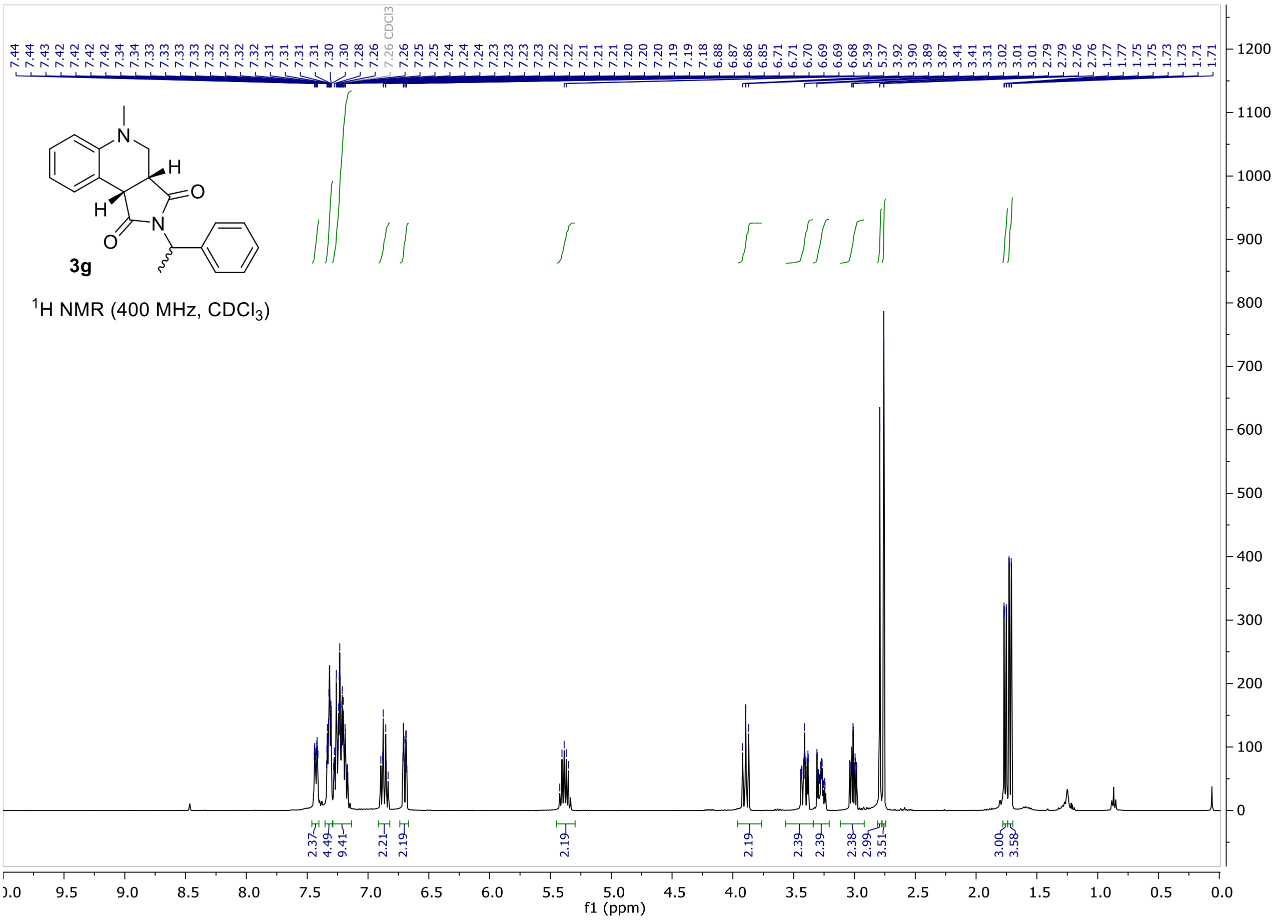
3g
^{1}H NMR (400 MHz, CDCl$_3$)
f1 (ppm)

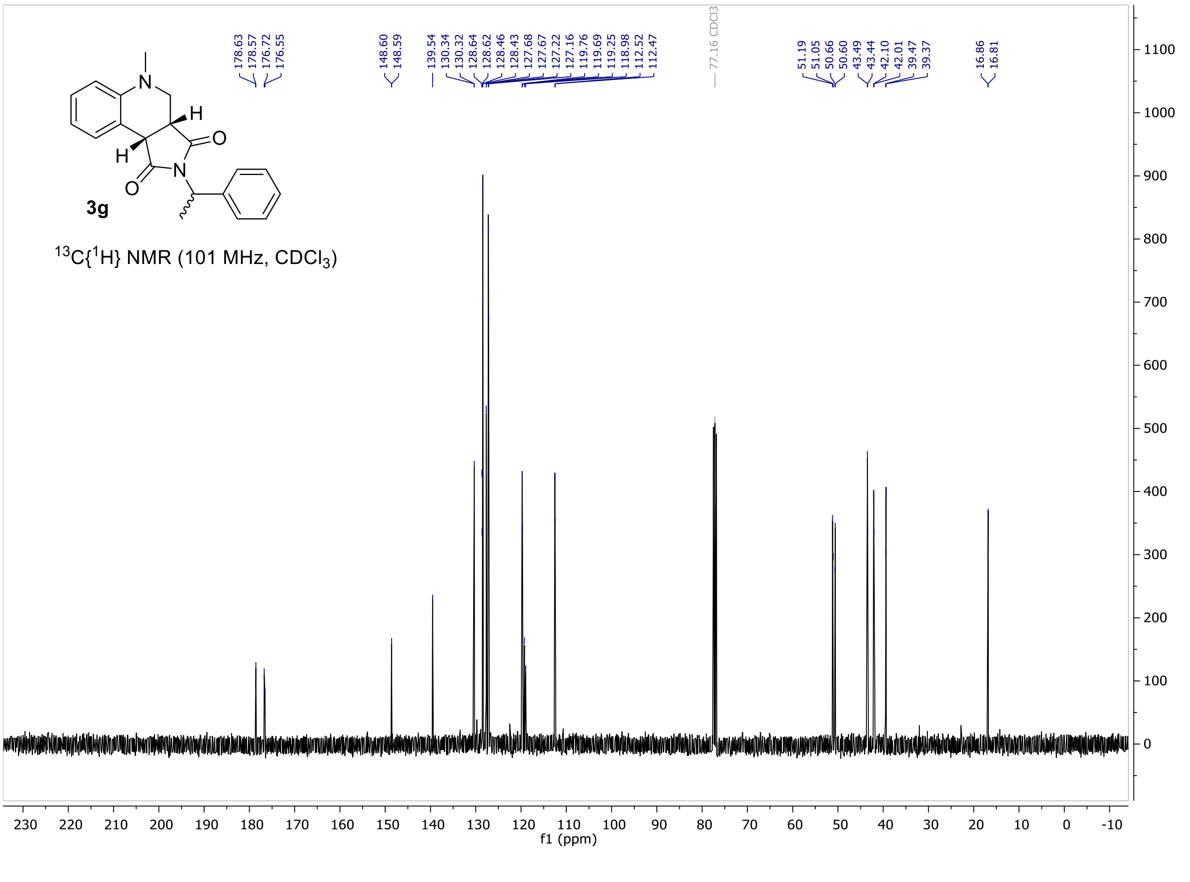
3g
^{13}C{^{1}H} NMR (101 MHz, CDCl$_3$)
f1 (ppm)

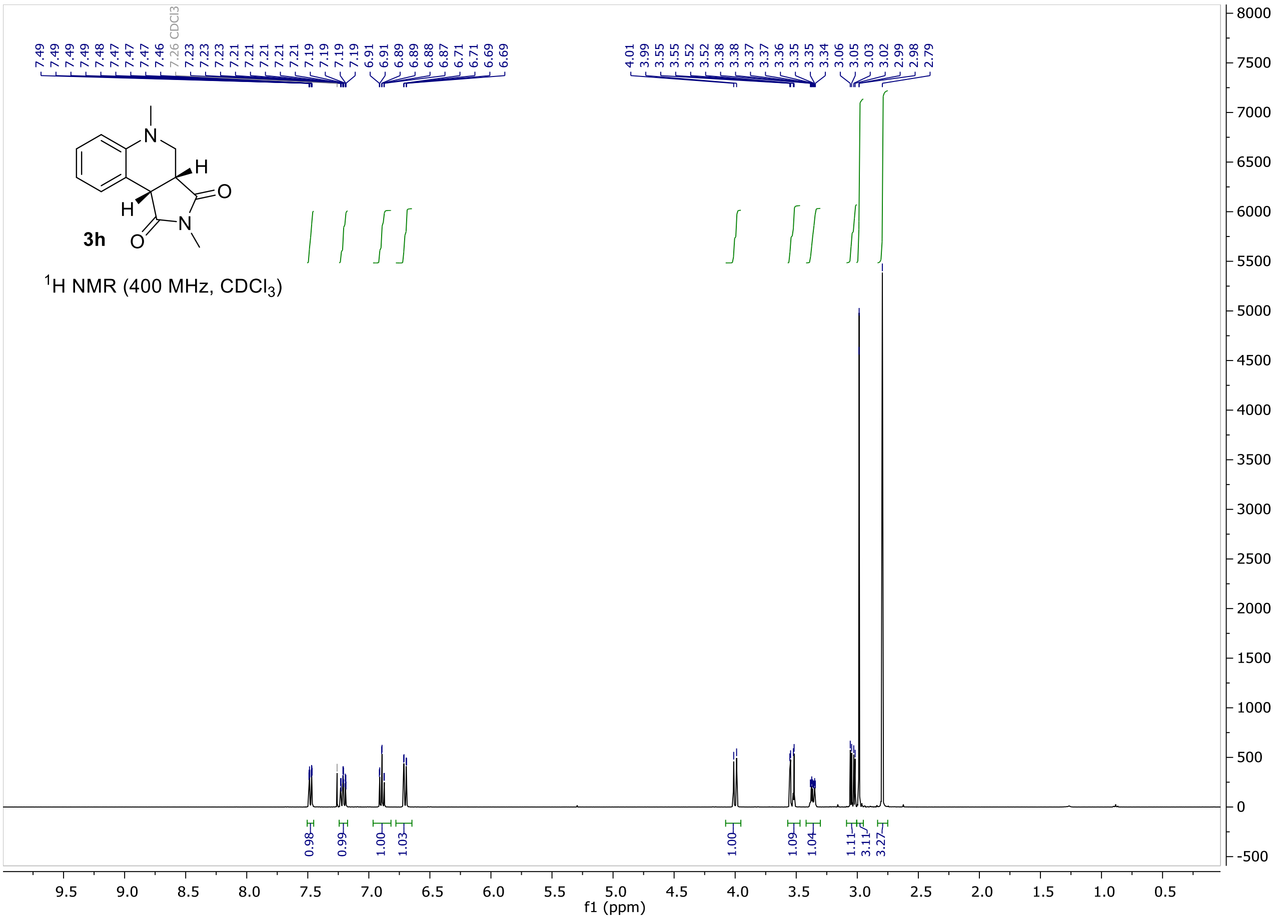
3h
1H NMR (400 MHz, CDCl3)
f1 (ppm)

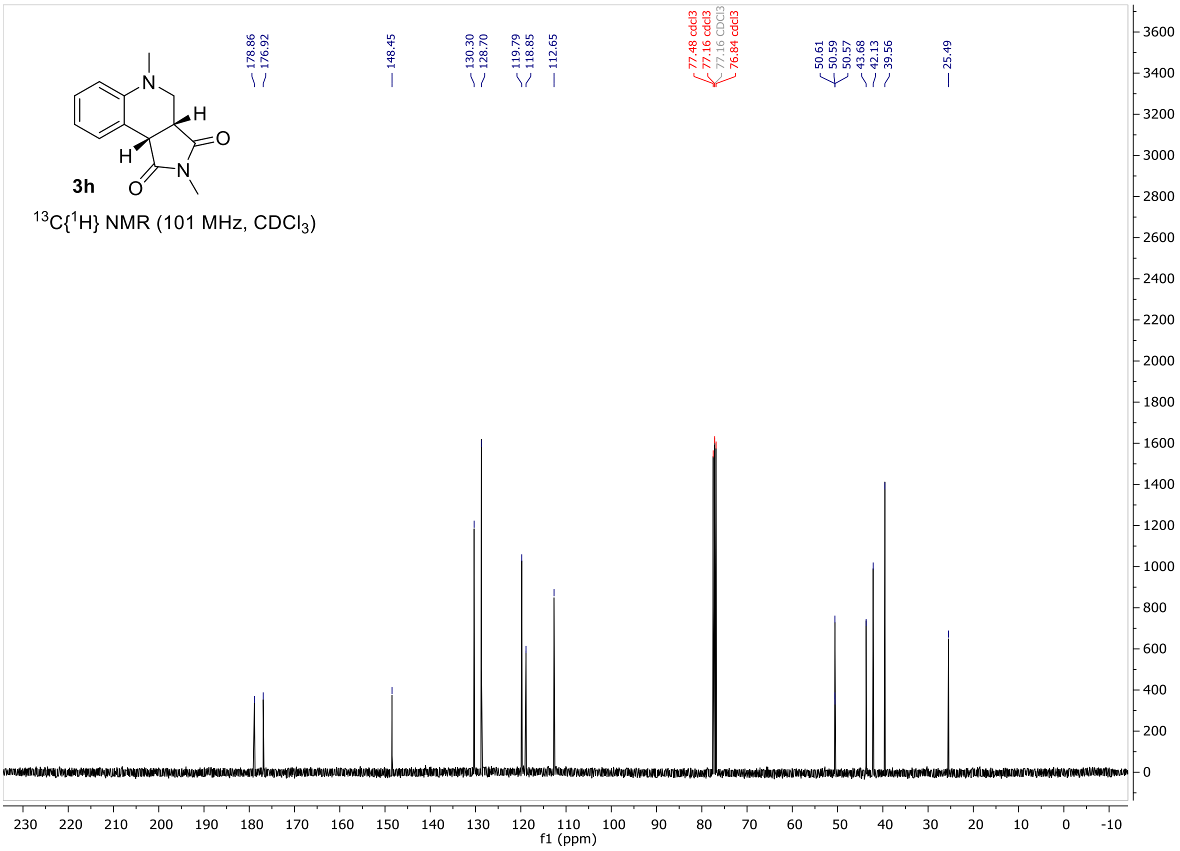
3h
13C{1H} NMR (101 MHz, CDCl3)
f1 (ppm)

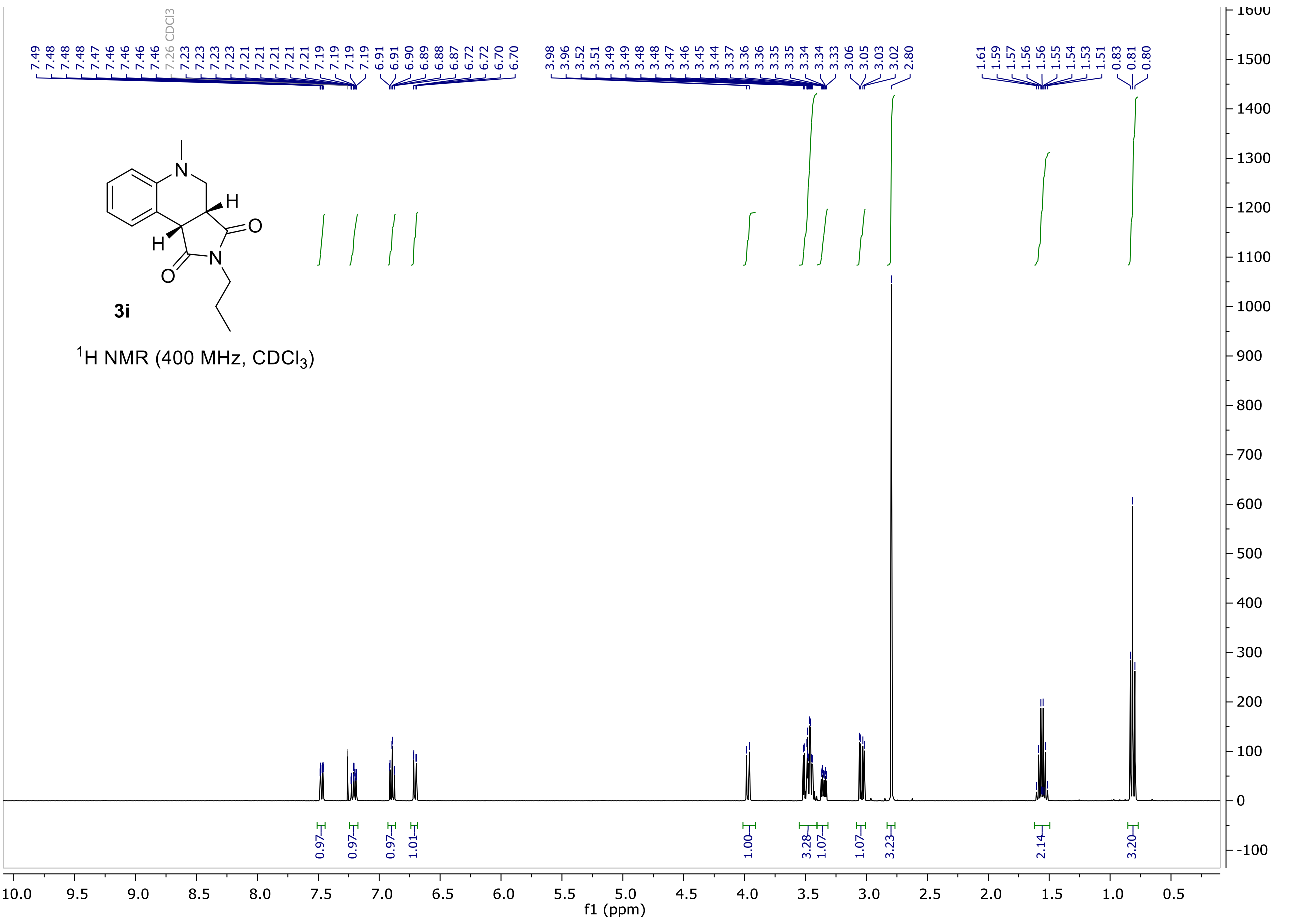
3i
^{1}H NMR (400 MHz, CDCl$_3$)
f1 (ppm)

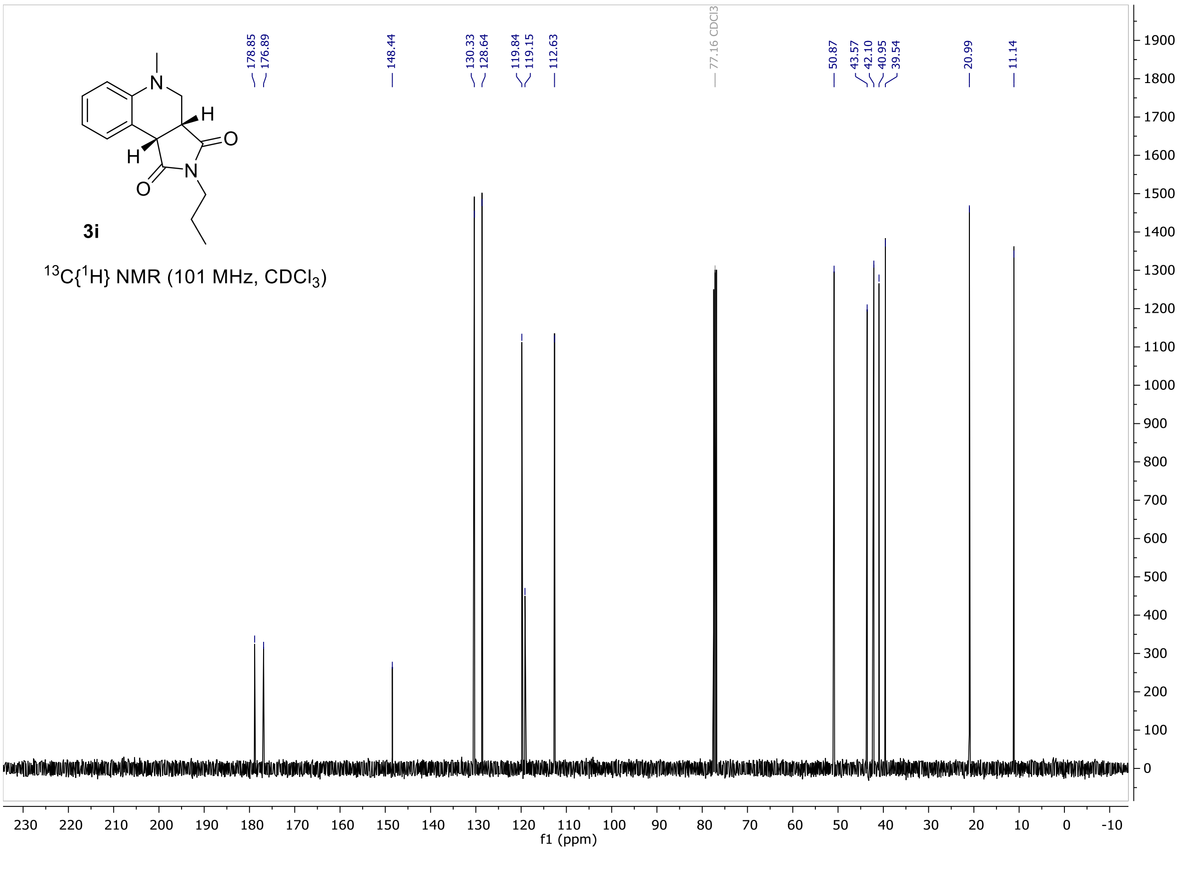
3i
^{13}C{^{1}H} NMR (101 MHz, CDCl$_3$)
f1 (ppm)

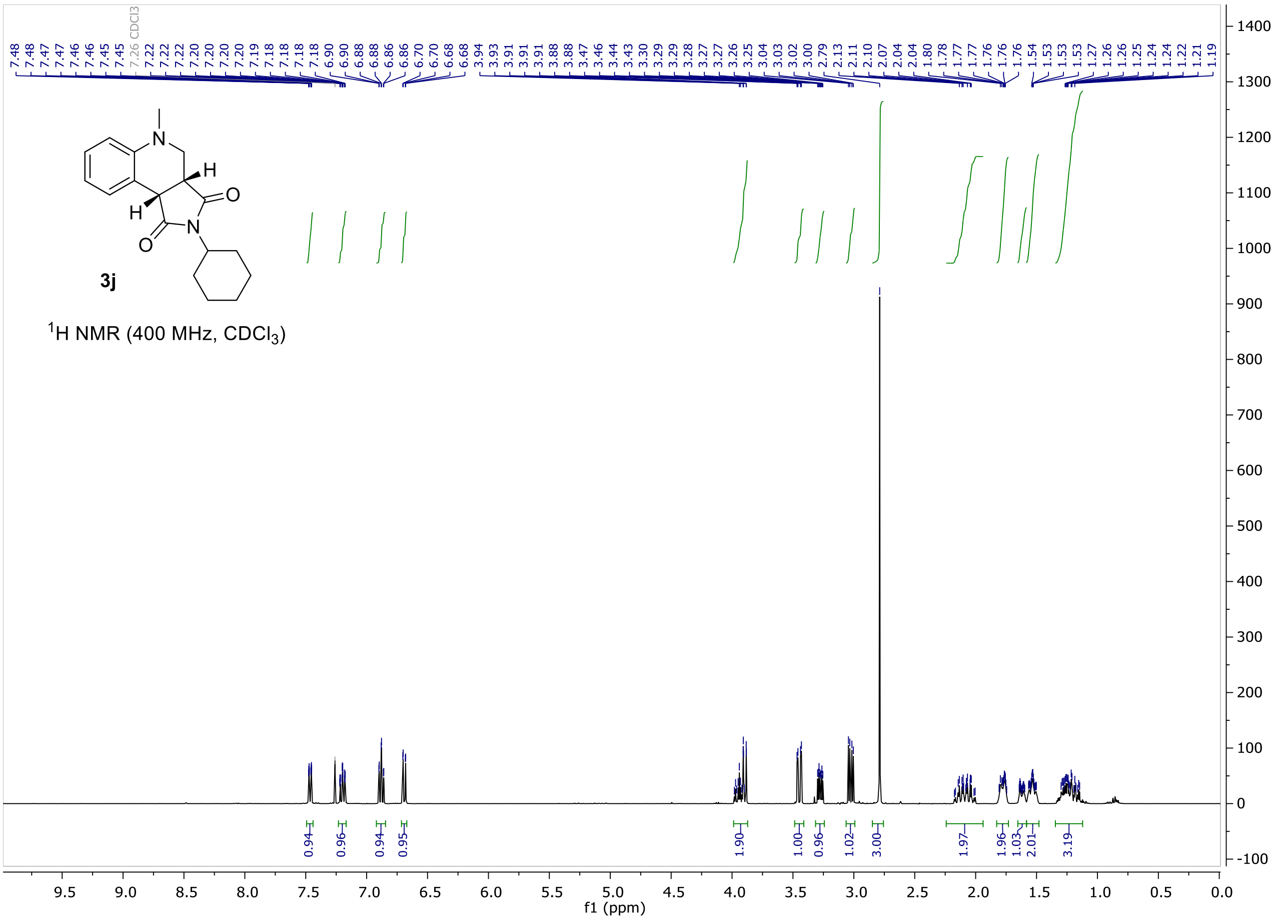
3j
^{1}H NMR (400 MHz, CDCl$_3$)
7.48 7.48 7.47 7.47 7.46 7.46 7.45 7.45 7.26 CDCl3 7.22 7.22 7.22 7.22 7.20 7.20 7.20 7.20 7.19 7.18 7.18 7.18 7.18 6.90 6.90 6.88 6.88 6.86 6.86 6.70 6.70 6.68 6.68 3.94 3.93 3.91 3.91 3.91 3.88 3.47 3.46 3.44 3.43 3.30 3.29 3.29 3.28 3.27 3.27 3.26 3.25 3.04 3.03 3.02 3.00 2.79 2.13 2.11 2.10 2.07 2.04 2.04 1.80 1.78 1.77 1.77 1.76 1.76 1.76 1.54 1.53 1.53 1.53 1.27 1.26 1.25 1.24 1.24 1.22 1.21 1.19
0.94 0.96 0.94 0.95 1.90 1.00 0.96 1.02 3.00 1.97 1.96 1.03 2.01 3.19
f1 (ppm)

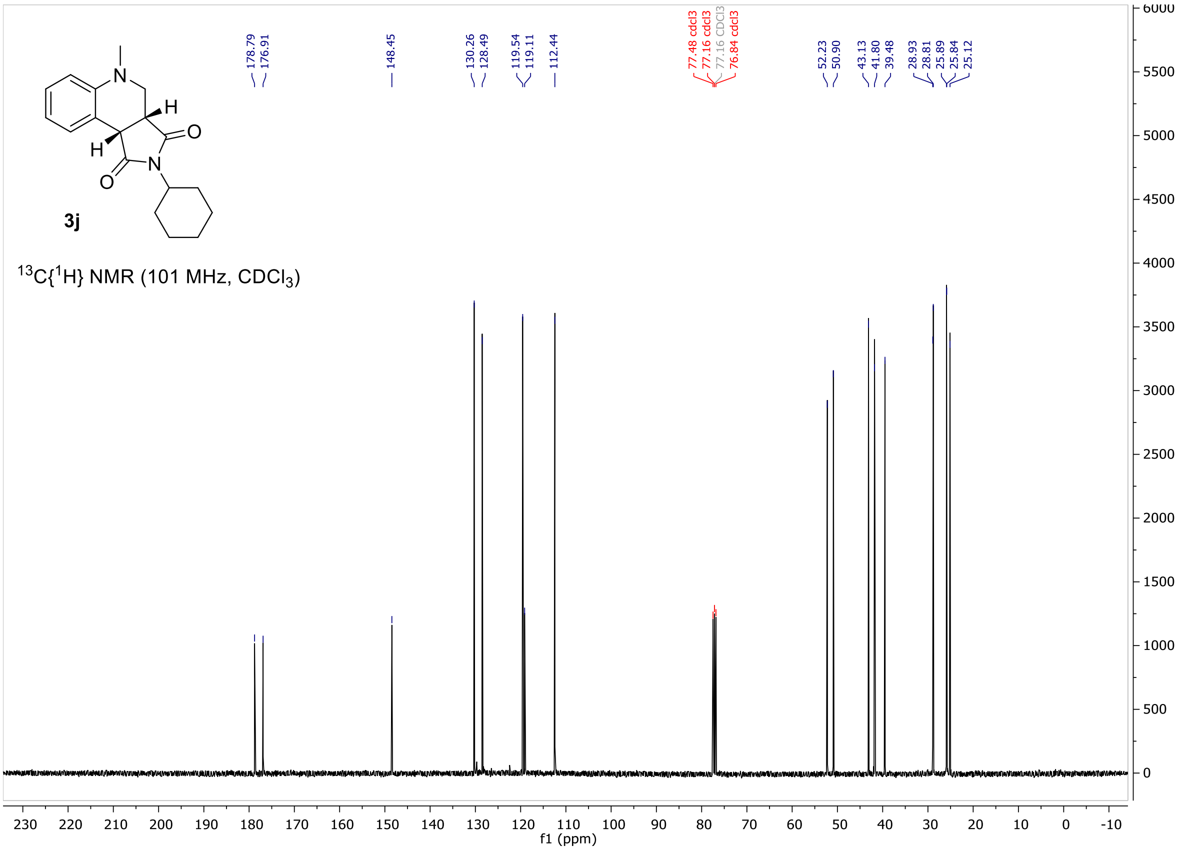
3j
^{13}C{^{1}H} NMR (101 MHz, CDCl$_3$)
178.79 176.91 148.45 130.26 128.49 119.54 119.11 112.44 77.48 cdcl3 77.16 cdcl3 77.16 CDCl3 76.84 cdcl3 52.23 50.90 43.13 41.80 39.48 28.93 28.81 25.89 25.84 25.12
f1 (ppm)

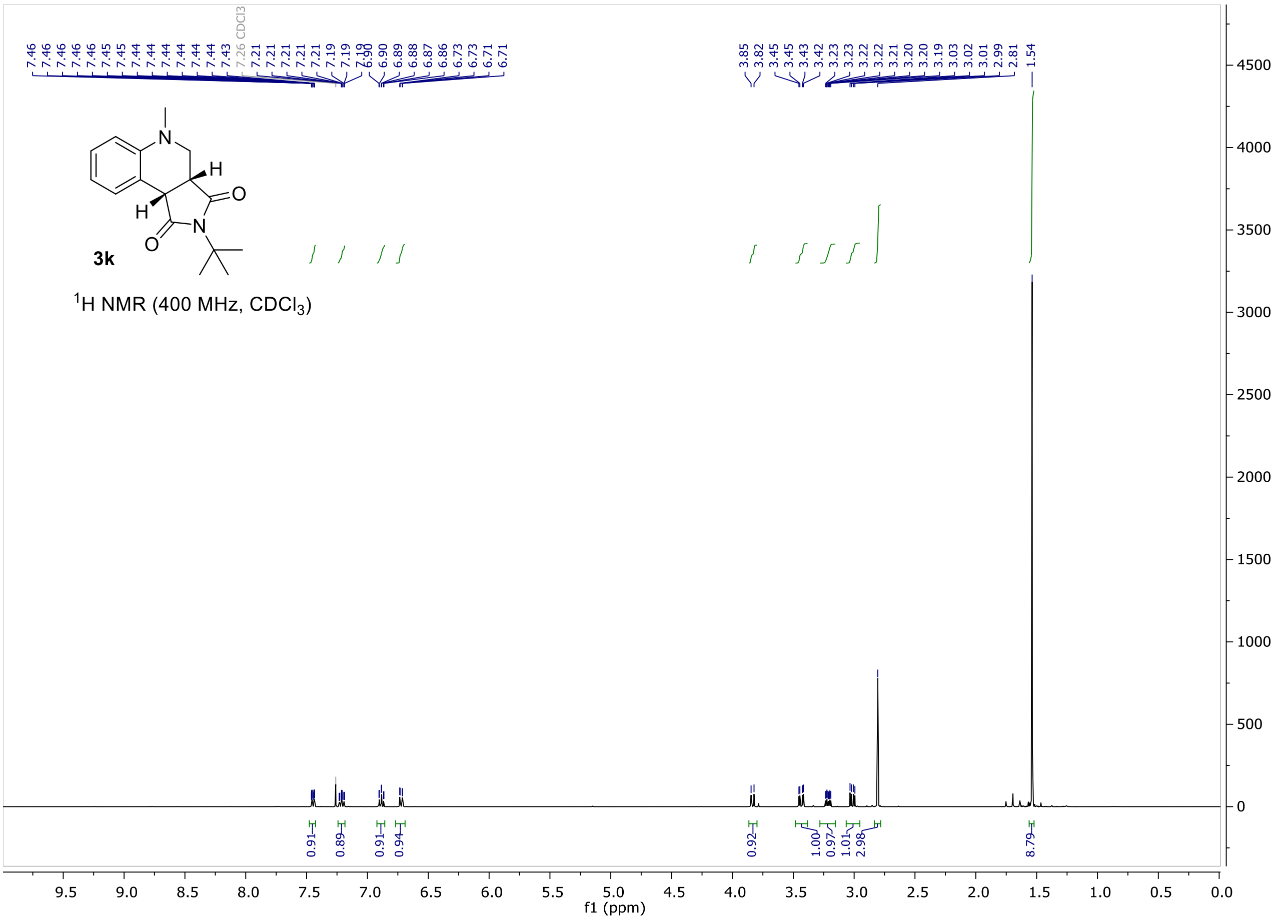
3k
1H NMR (400 MHz, CDCl3)
f1 (ppm)

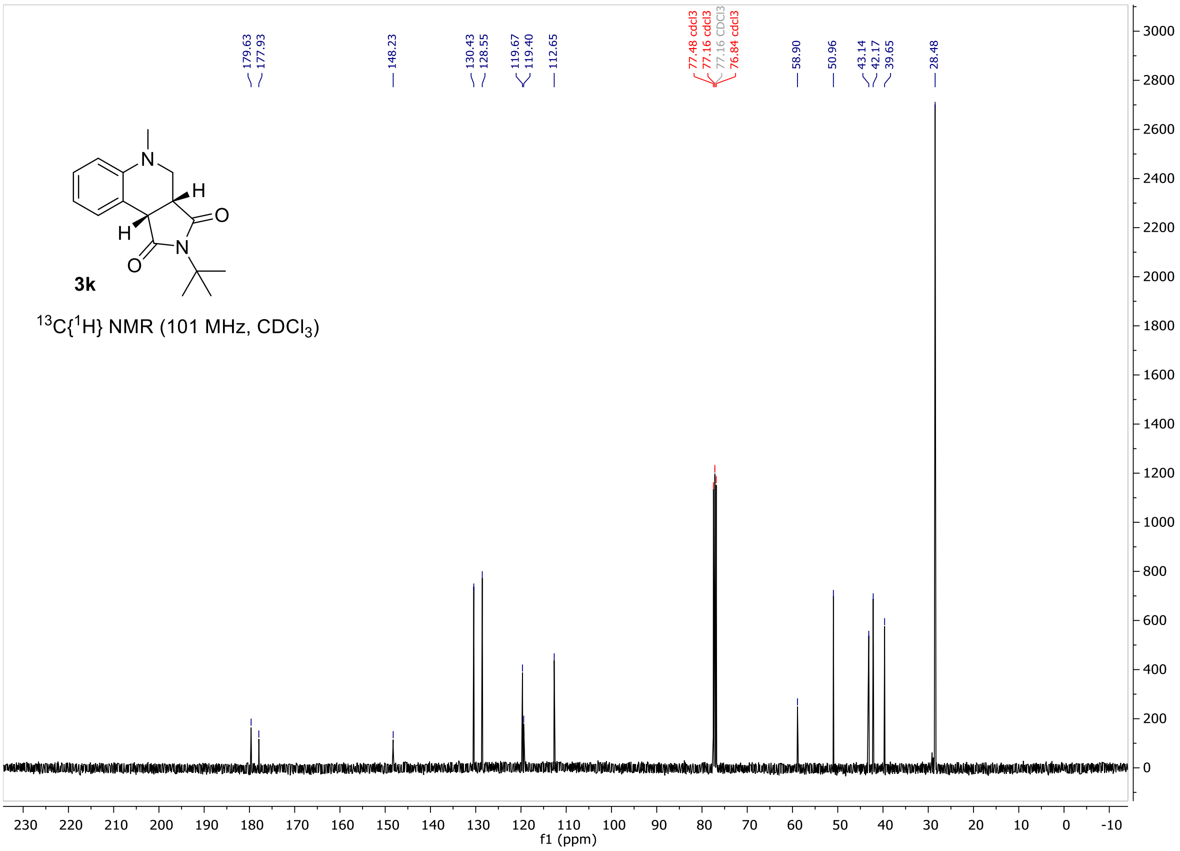
3k
13C{1H} NMR (101 MHz, CDCl3)
179.63
177.93
148.23
130.43
128.55
119.67
119.40
112.65
77.48 cdcl3
77.16 cdcl3
77.16 CDCl3
76.84 cdcl3
58.90
50.96
43.14
42.17
39.65
28.48
f1 (ppm)

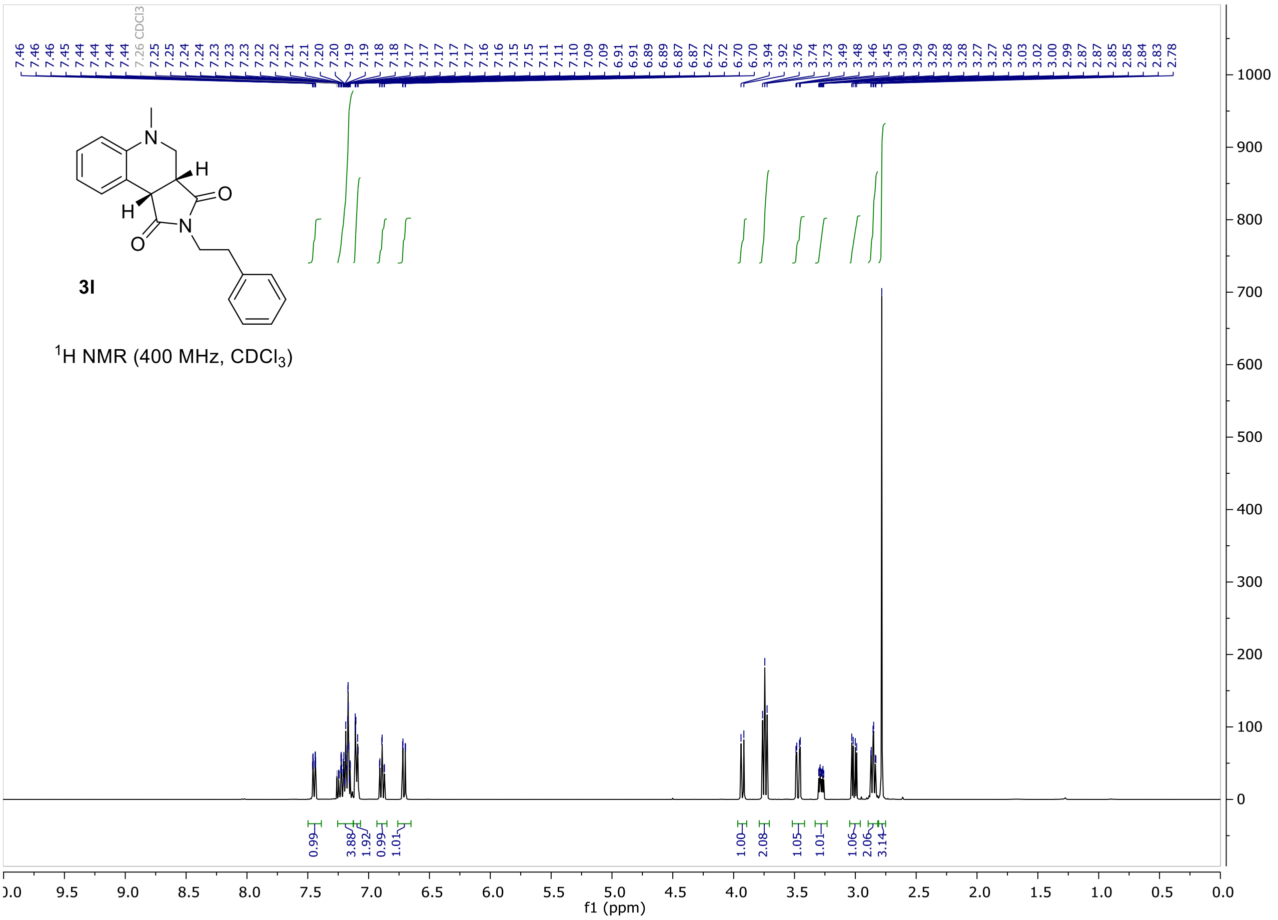
3l
1H NMR (400 MHz, CDCl3)
f1 (ppm)

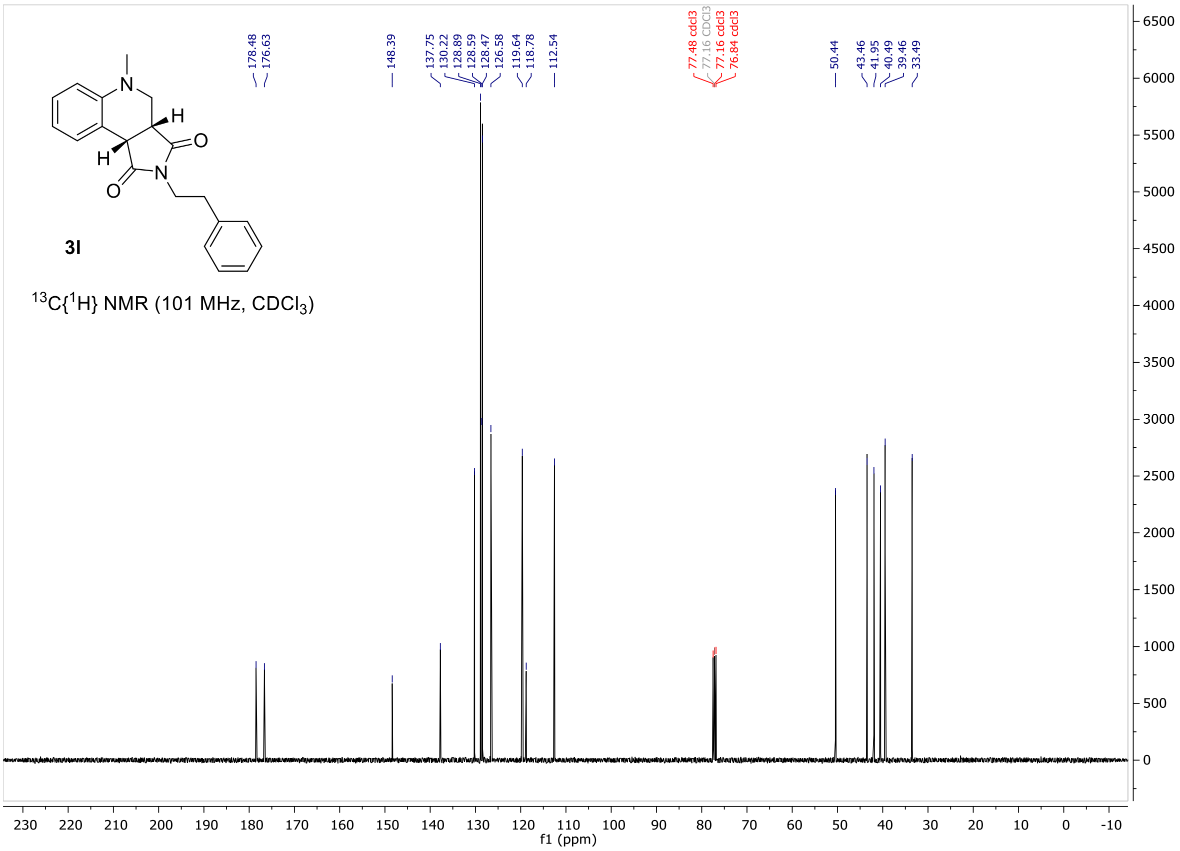
3l
13C{1H} NMR (101 MHz, CDCl3)
178.48
176.63
148.39
137.75
130.22
128.89
128.59
128.47
126.58
119.64
118.78
112.54
77.48 cdcl3
77.16 CDCl3
77.16 cdcl3
76.84 cdcl3
50.44
43.46
41.95
40.49
39.46
33.49
f1 (ppm)

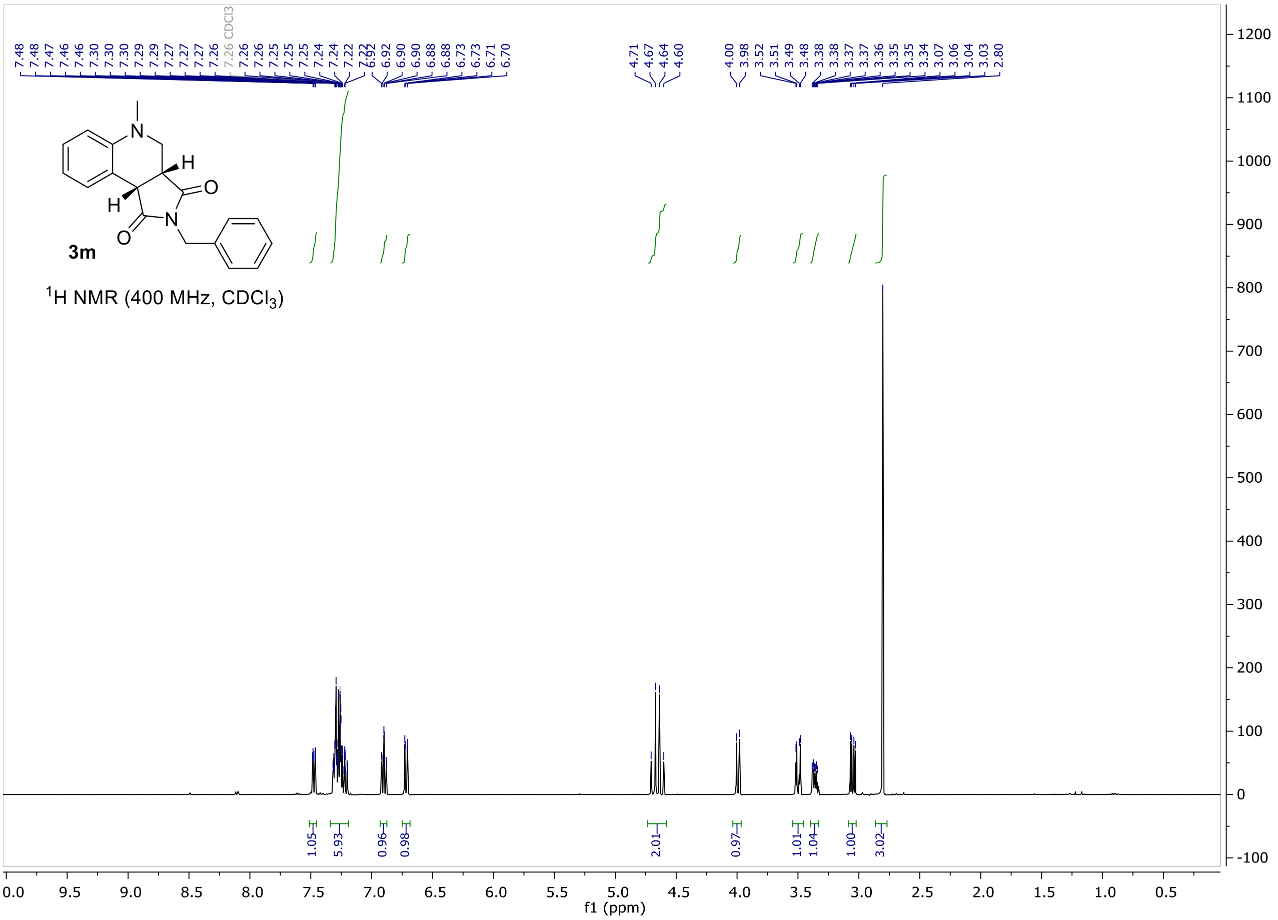

3m
^{1}H NMR (400 MHz, CDCl$_3$)
f1 (ppm)

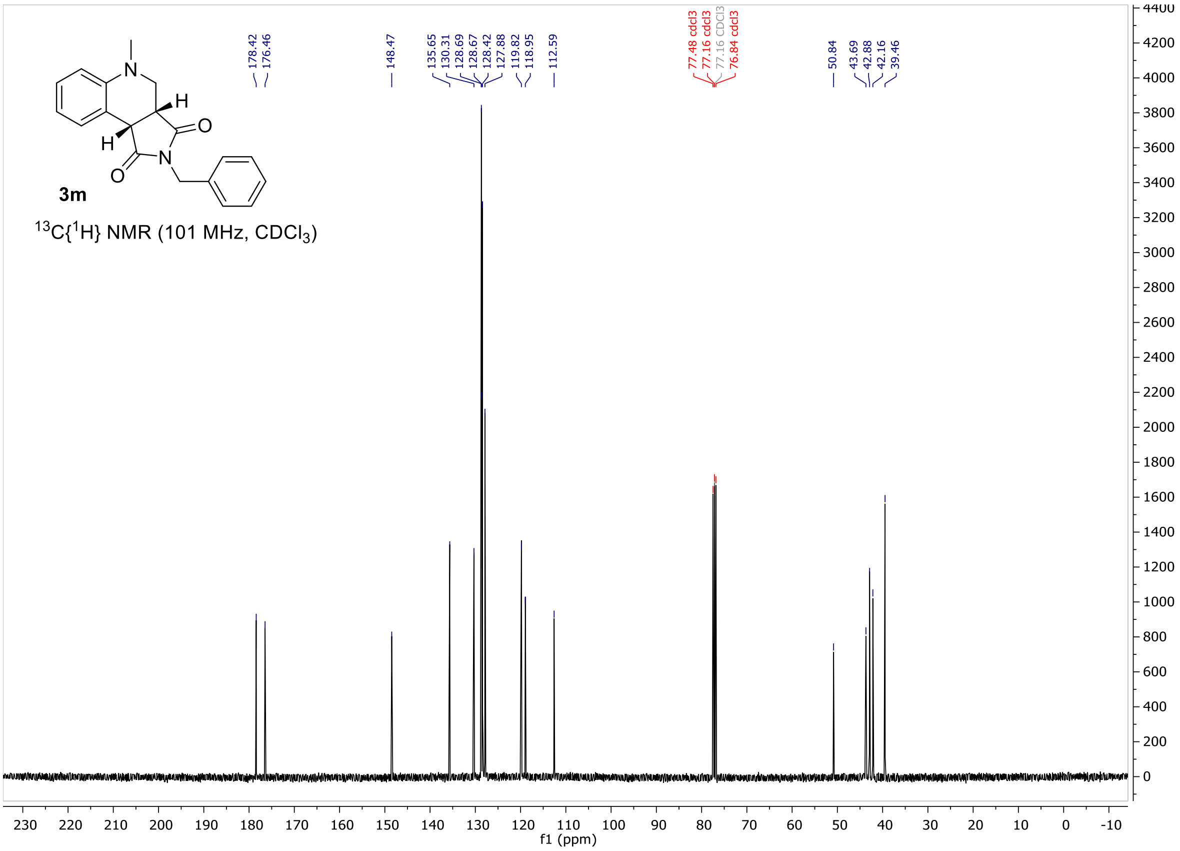

3m
^{13}C{^{1}H} NMR (101 MHz, CDCl$_3$)
f1 (ppm)

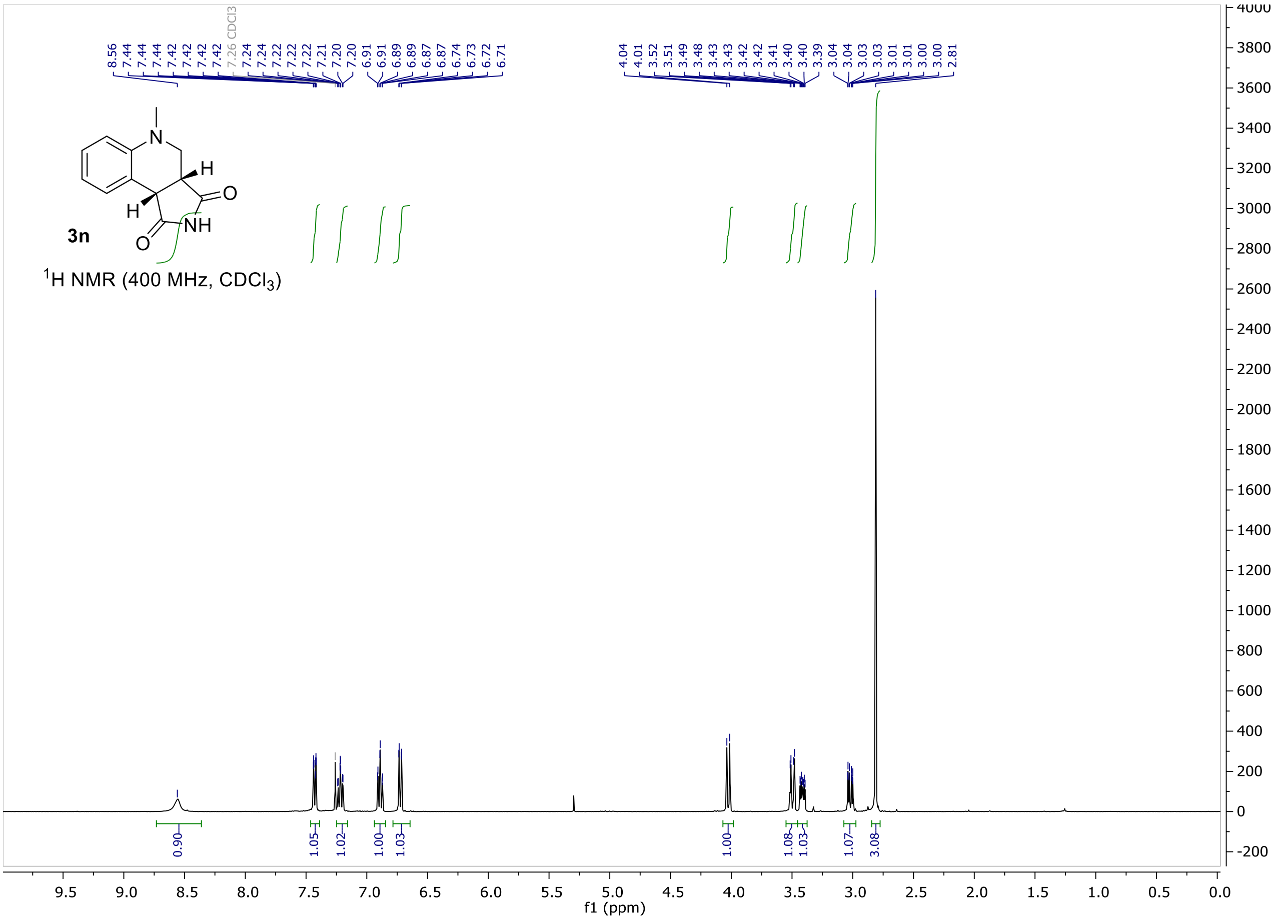
3n
^{1}H NMR (400 MHz, CDCl$_3$)
f1 (ppm)

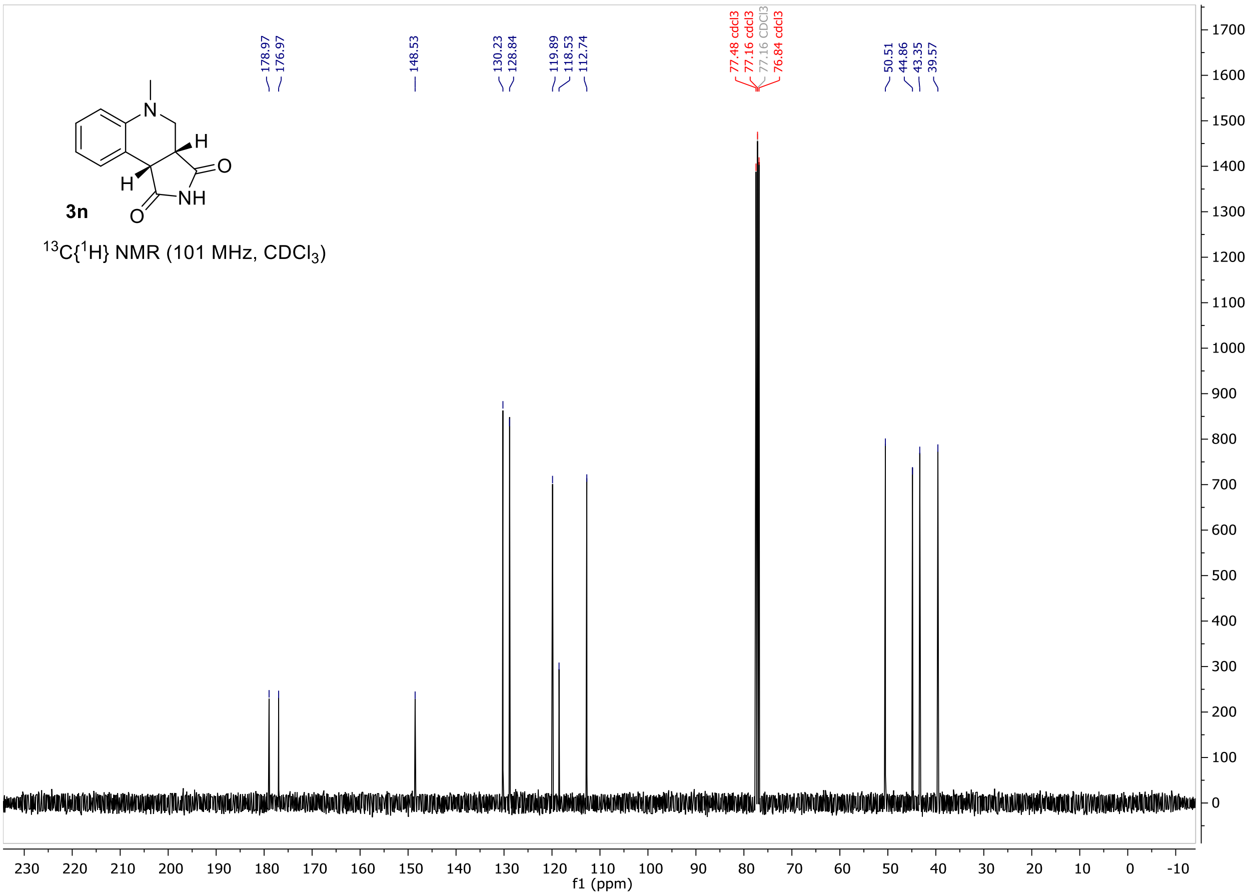
3n
^{13}C{^{1}H} NMR (101 MHz, CDCl$_3$)
f1 (ppm)

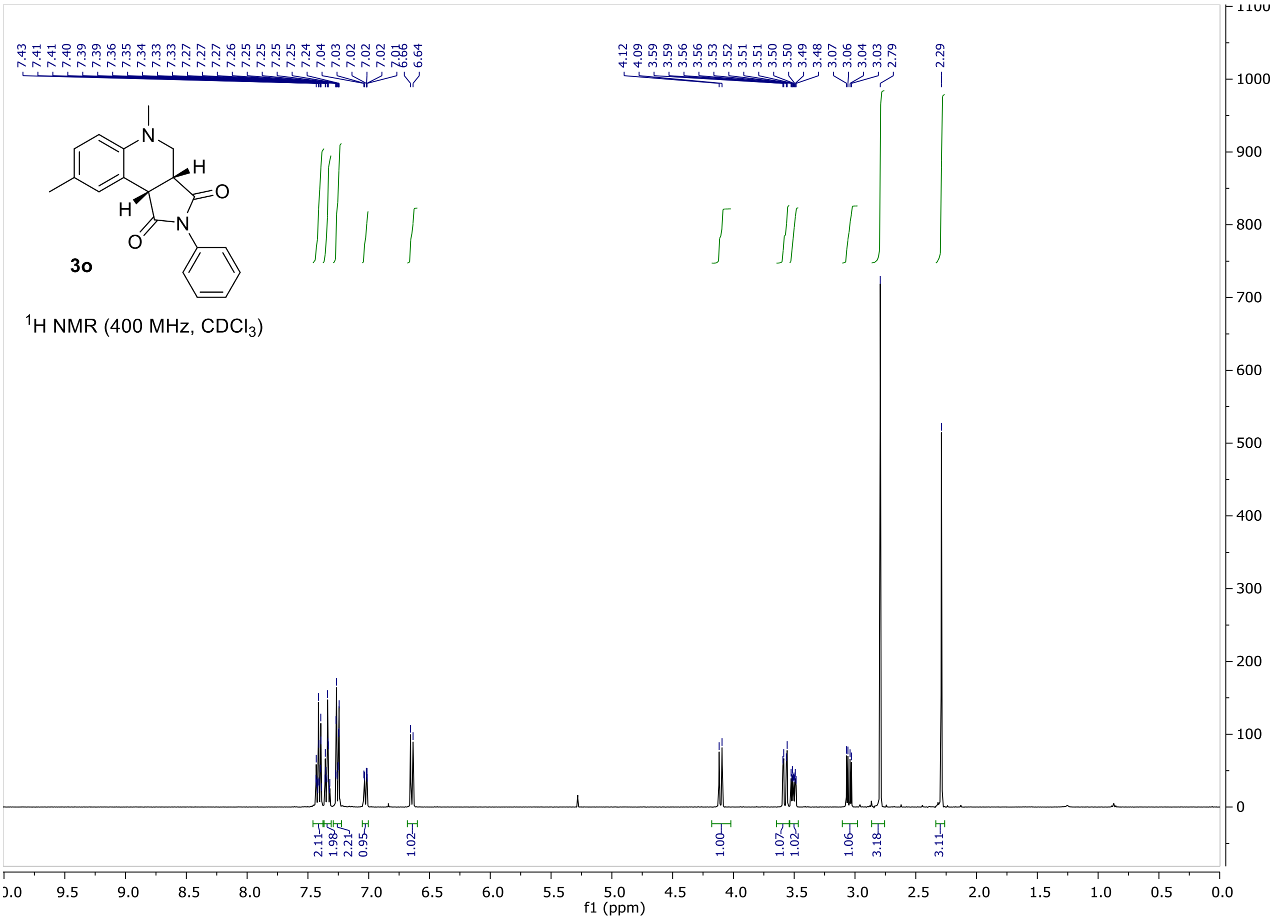
3o
^{1}H NMR (400 MHz, CDCl$_3$)
f1 (ppm)

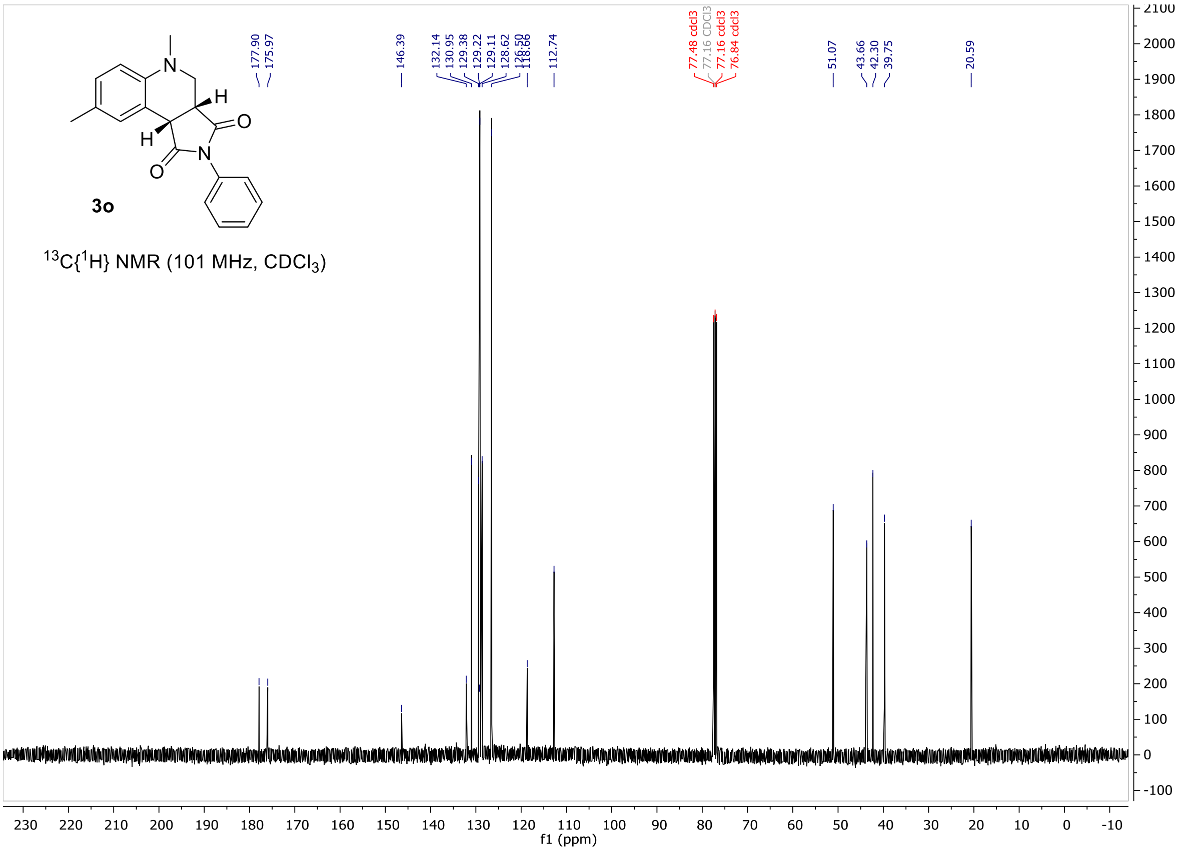
3o
^{13}C{^{1}H} NMR (101 MHz, CDCl$_3$)
177.90
175.97
146.39
132.14
130.95
129.38
129.22
129.11
128.62
112.74
51.07
43.66
42.30
39.75
20.59
77.48 cdcl3
77.16 CDCl3
77.16 cdcl3
76.84 cdcl3
f1 (ppm)

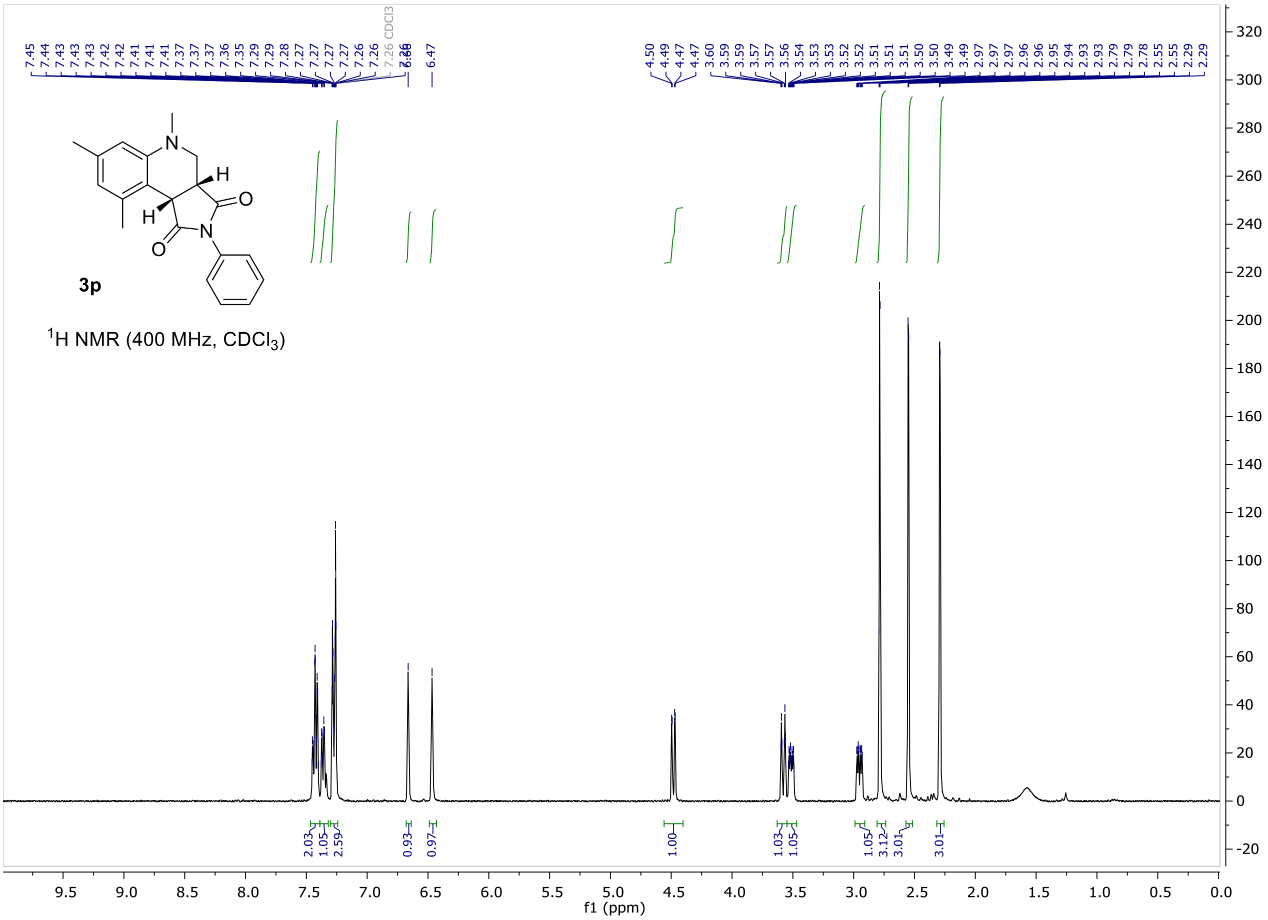
7.45 7.44 7.43 7.43 7.43 7.42 7.42 7.41 7.41 7.37 7.37 7.37 7.36 7.35 7.29 7.29 7.28 7.27 7.27 7.27 7.27 7.26 7.26 7.26 CDCl3 6.66 6.47
4.50 4.49 4.47 4.47 3.60 3.59 3.57 3.57 3.56 3.54 3.53 3.53 3.52 3.52 3.51 3.51 3.50 3.50 3.49 3.49 2.97 2.97 2.97 2.96 2.95 2.94 2.93 2.93 2.79 2.79 2.78 2.55 2.55 2.29 2.29
N
H
O
H
O
N
3p
^{1}H NMR (400 MHz, CDCl$_3$)
2.03 1.05 2.59 0.93 0.97 1.00 1.03 1.05 1.05 3.12 3.01 3.01
f1 (ppm)

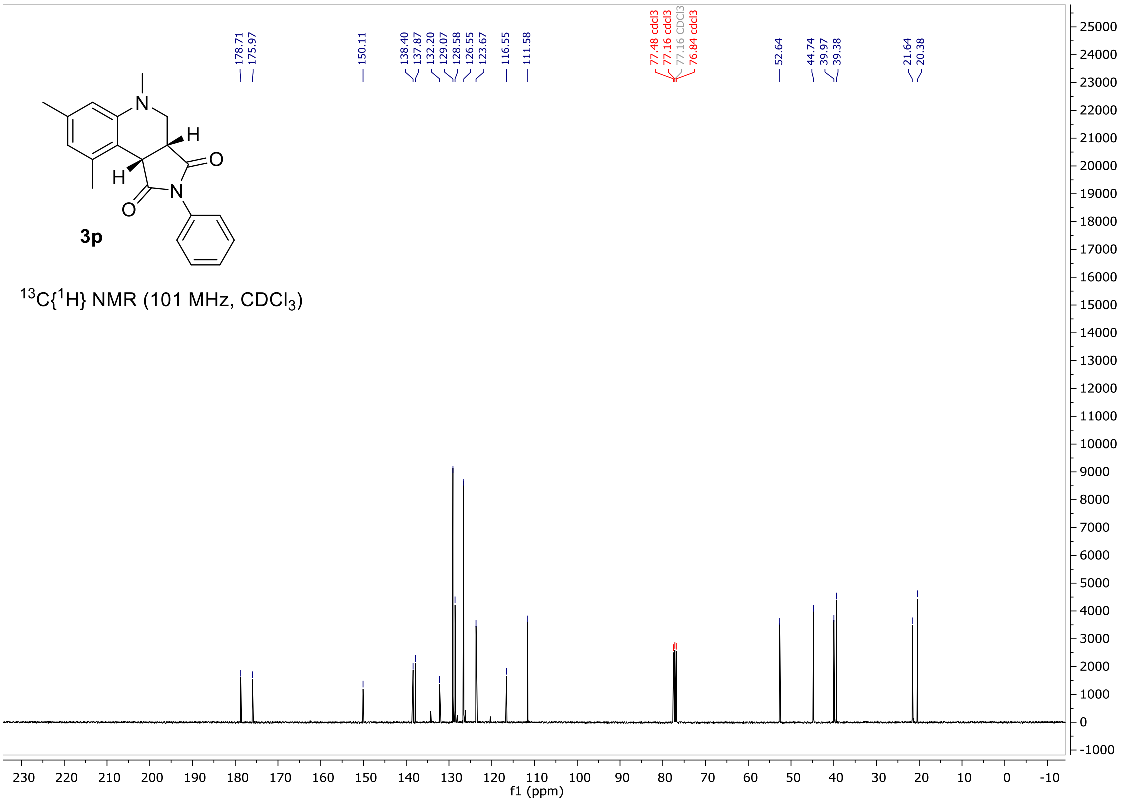
178.71 175.97 150.11 138.40 137.87 132.20 129.07 128.58 126.55 123.67 116.55 111.58
77.48 cdcl3 77.16 cdcl3 77.16 CDCl3 76.84 cdcl3
52.64 44.74 39.97 39.38 21.64 20.38
N
H
O
H
O
N
3p
^{13}C{^{1}H} NMR (101 MHz, CDCl$_3$)
f1 (ppm)

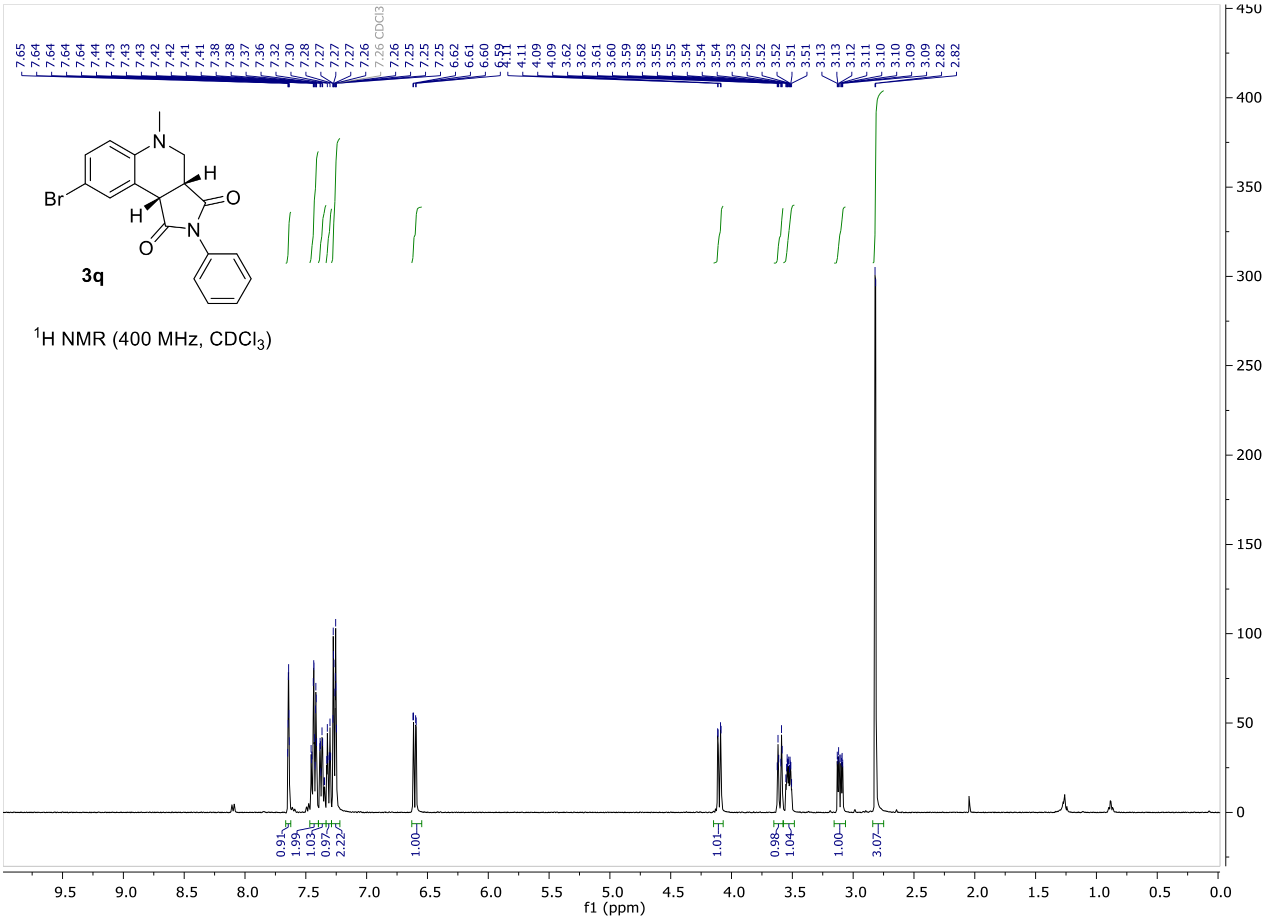
3q
^{1}H NMR (400 MHz, CDCl$_3$)
f1 (ppm)

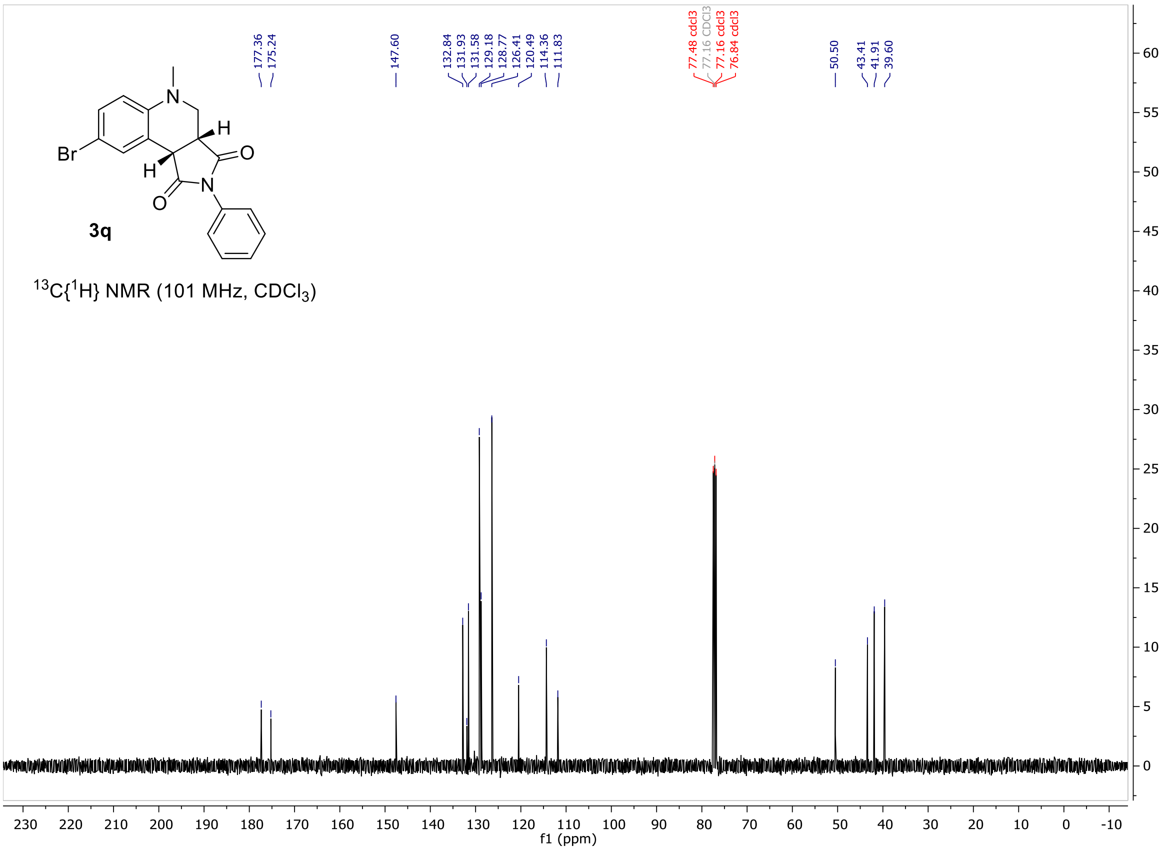
3q
^{13}C{^{1}H} NMR (101 MHz, CDCl$_3$)
f1 (ppm)

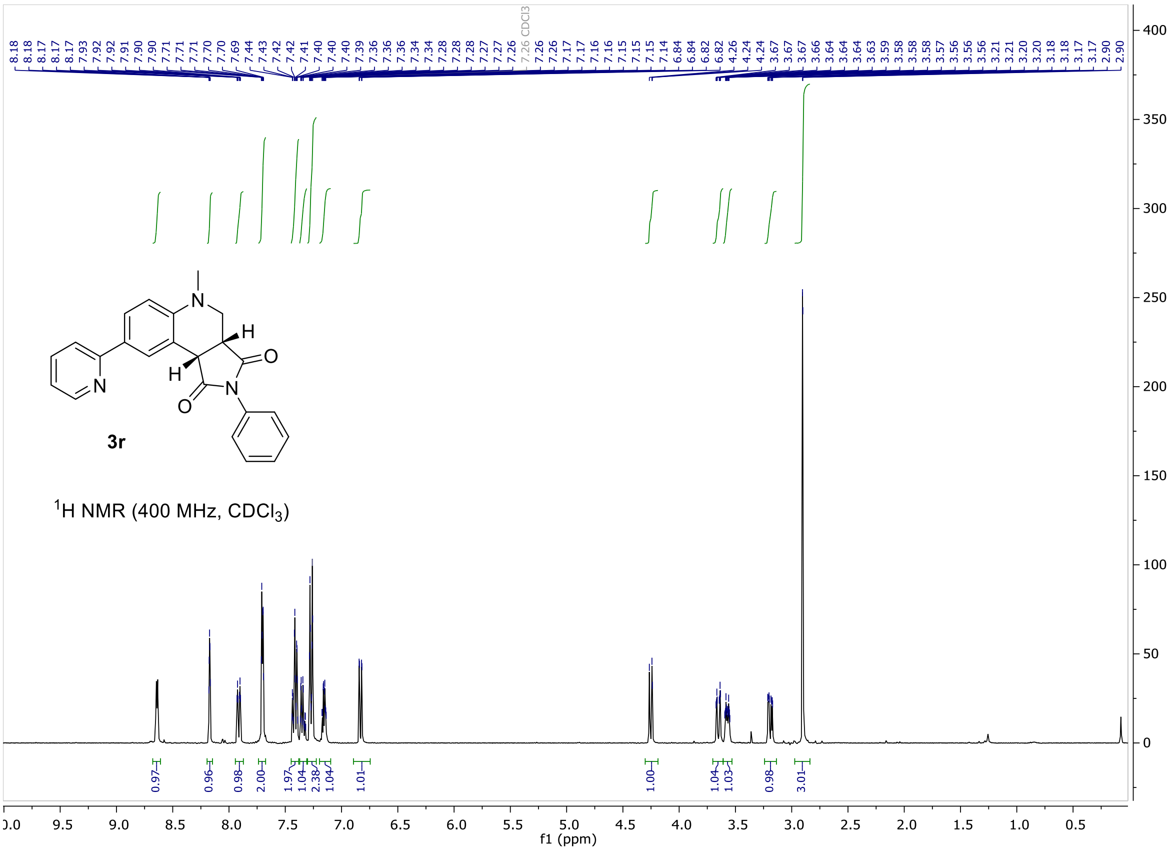
3r
^{1}H NMR (400 MHz, CDCl$_3$)
f1 (ppm)

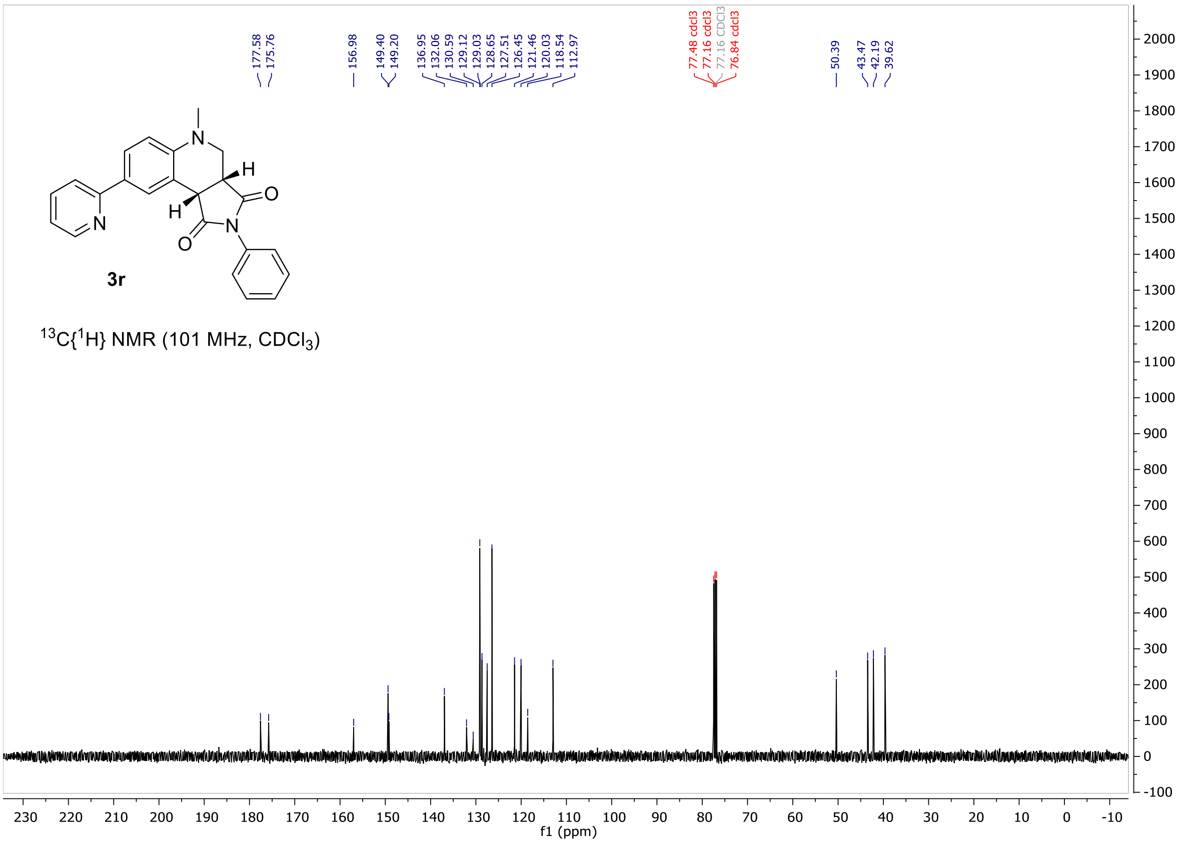
3r
^{13}C{^{1}H} NMR (101 MHz, CDCl$_3$)
f1 (ppm)

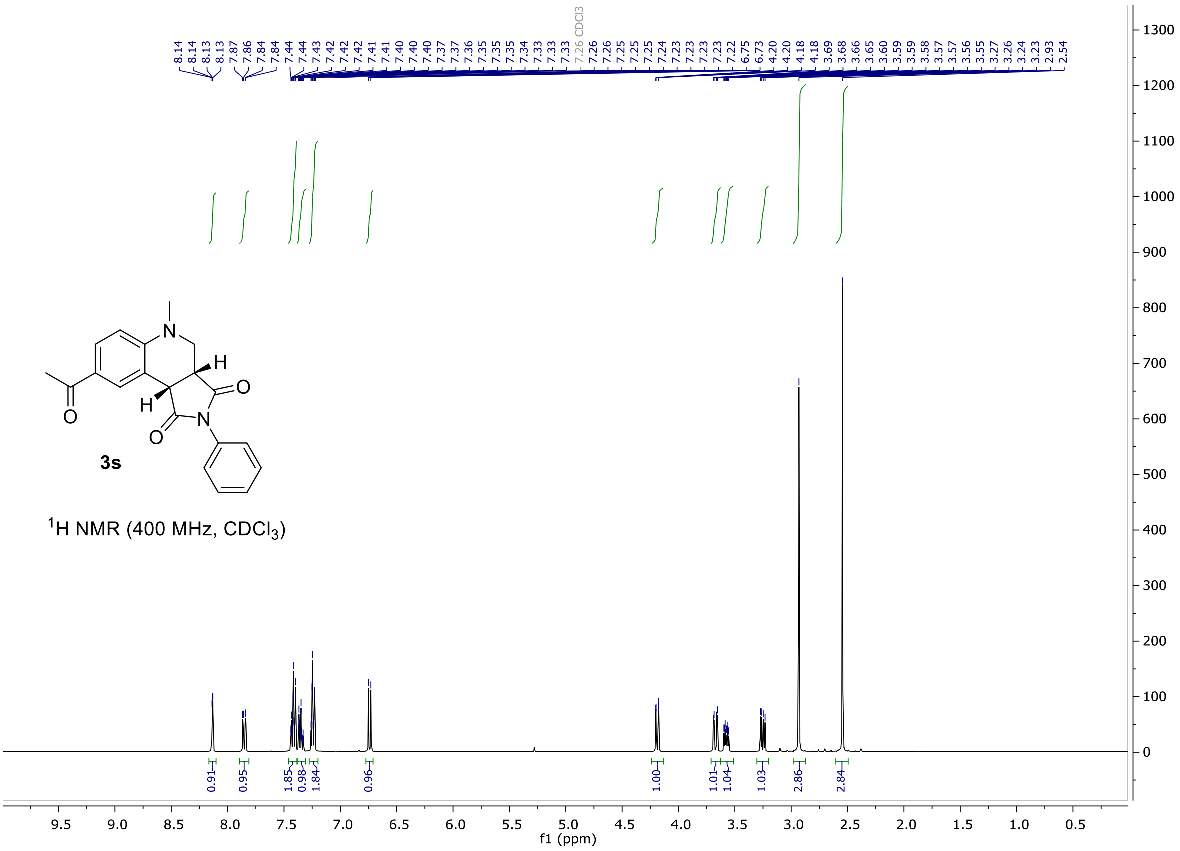
3s
1H NMR (400 MHz, CDCl3)
8.14
8.14
8.13
8.13
7.87
7.86
7.84
7.84
7.26 CDCl3
6.75
6.73
4.20
4.18
2.93
2.54
f1 (ppm)

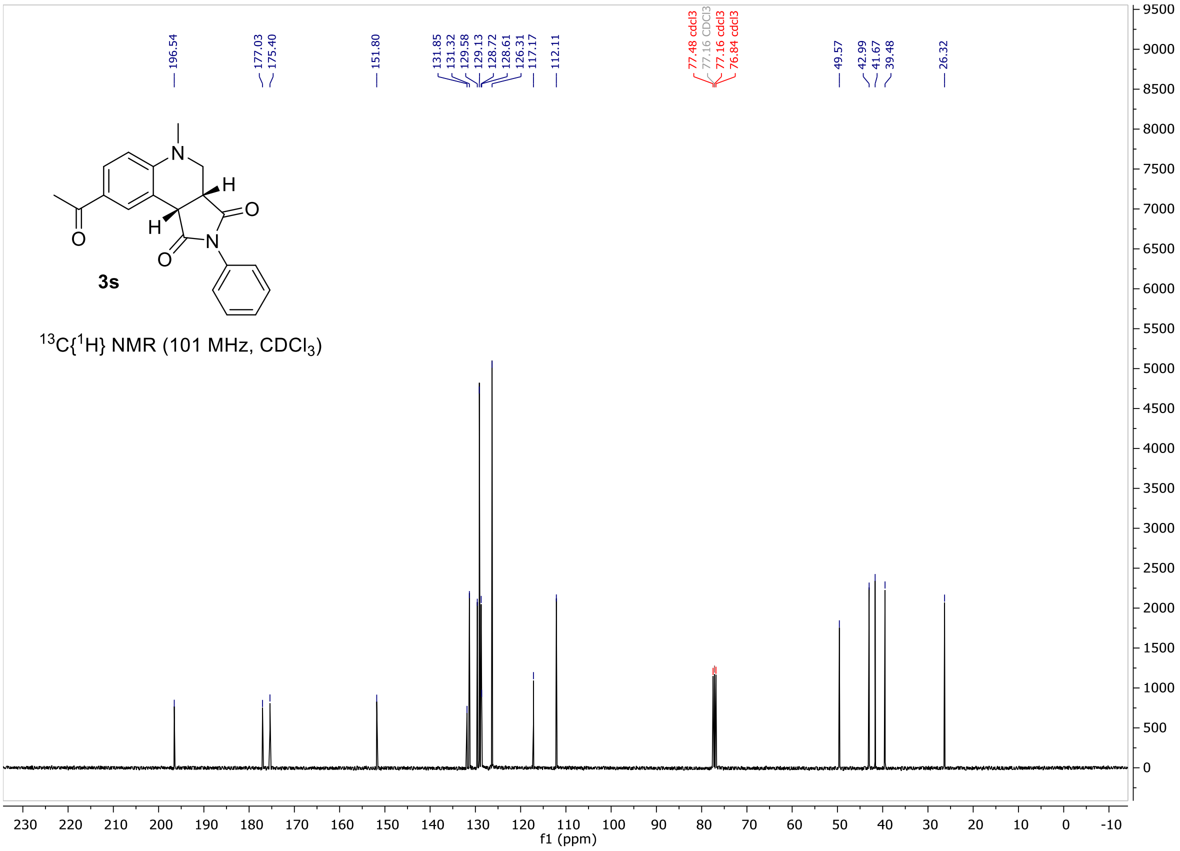
3s
13C{1H} NMR (101 MHz, CDCl3)
196.54
177.03
175.40
151.80
131.85
131.32
129.58
129.13
128.72
128.61
126.31
117.17
112.11
77.48 cdcl3
77.16 CDCl3
77.16 cdcl3
76.84 cdcl3
49.57
42.99
41.67
39.48
26.32
f1 (ppm)

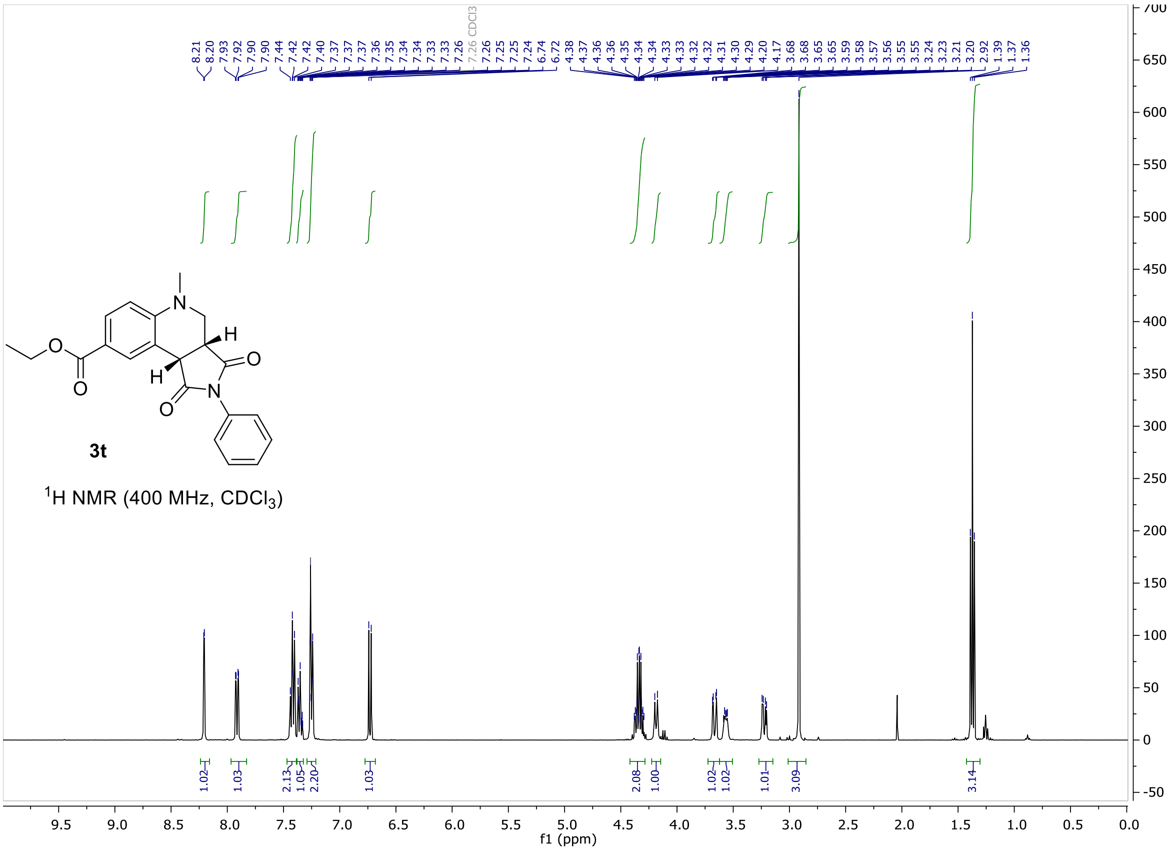
3t
^{1}H NMR (400 MHz, $CDCl_3$)
f1 (ppm)

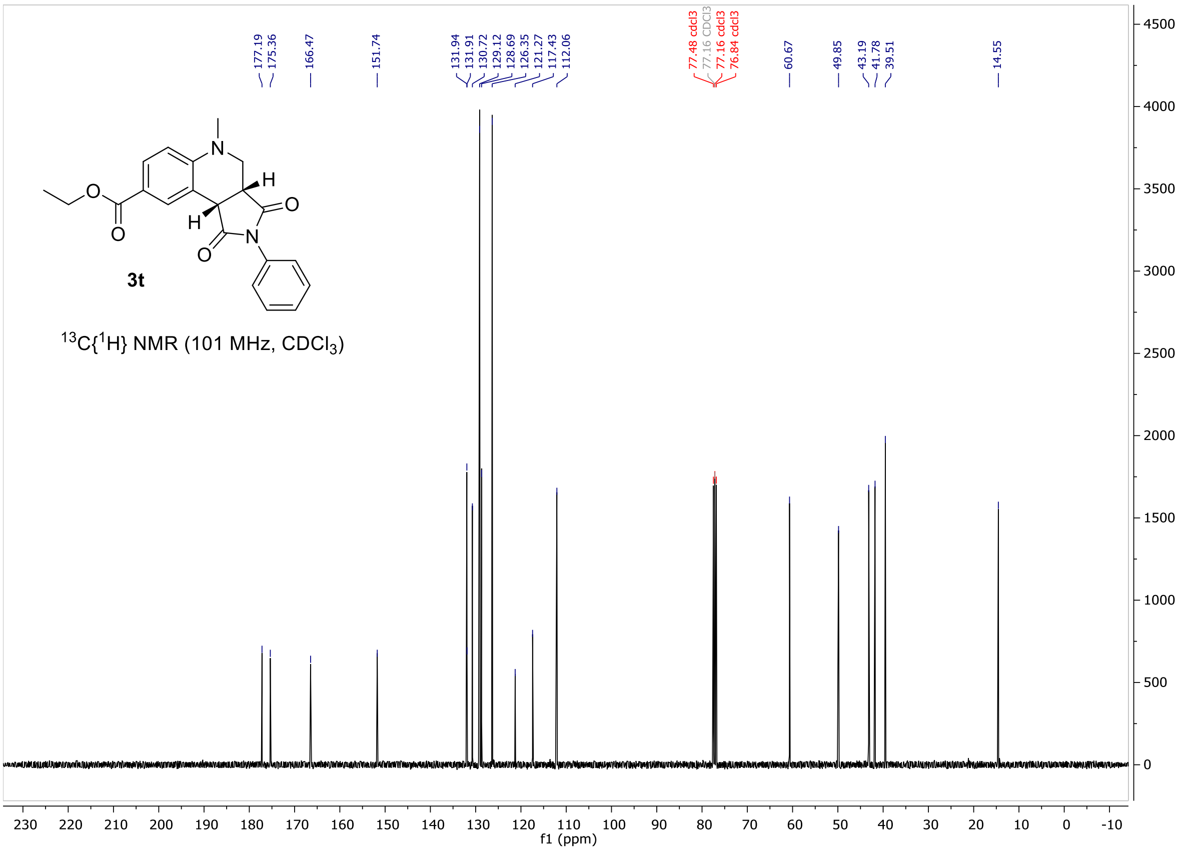
177.19
175.36
166.47
151.74
131.94
131.91
130.72
129.12
128.69
126.35
121.27
117.43
112.06
77.48 cdcl3
77.16 CDCl3
77.16 cdcl3
76.84 cdcl3
60.67
49.85
43.19
41.78
39.51
14.55
3t
^{13}C{^{1}H} NMR (101 MHz, $CDCl_3$)
f1 (ppm)

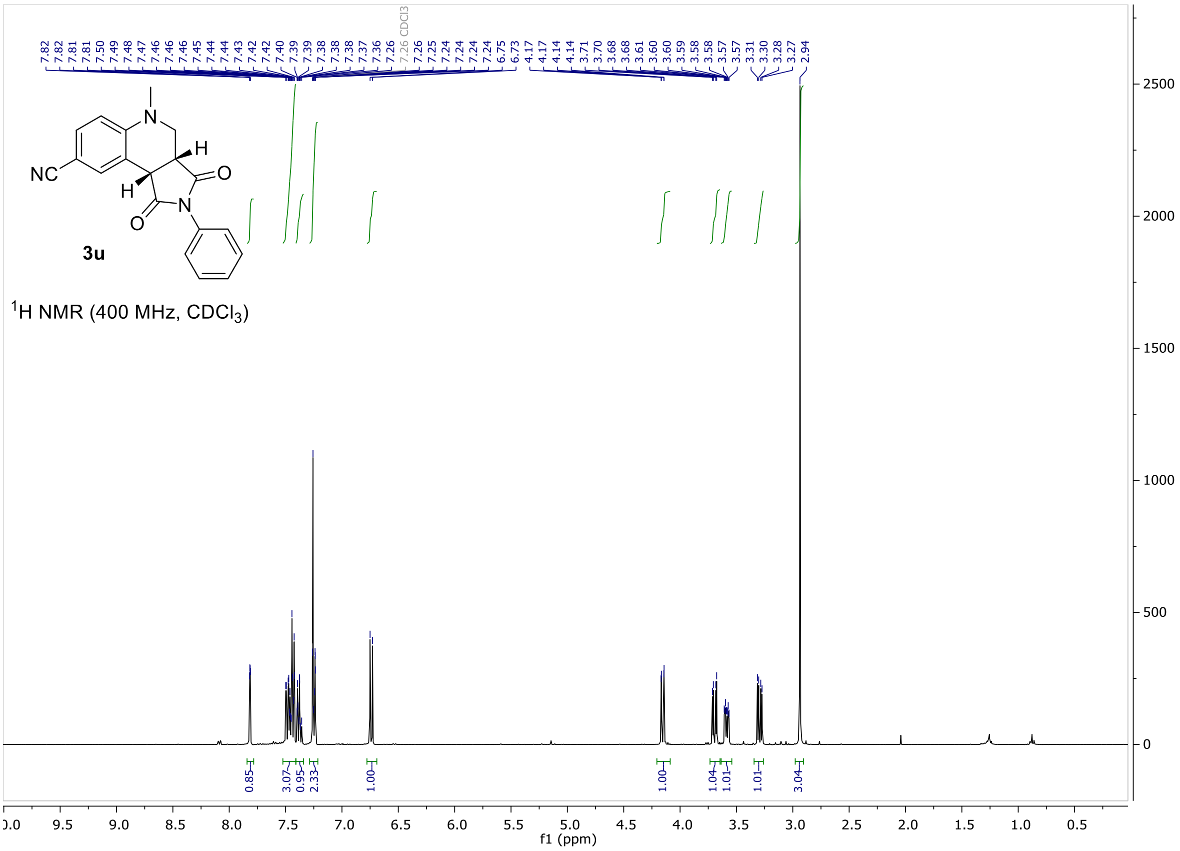

NC
3u
^{1}H NMR (400 MHz, CDCl$_3$)
f1 (ppm)

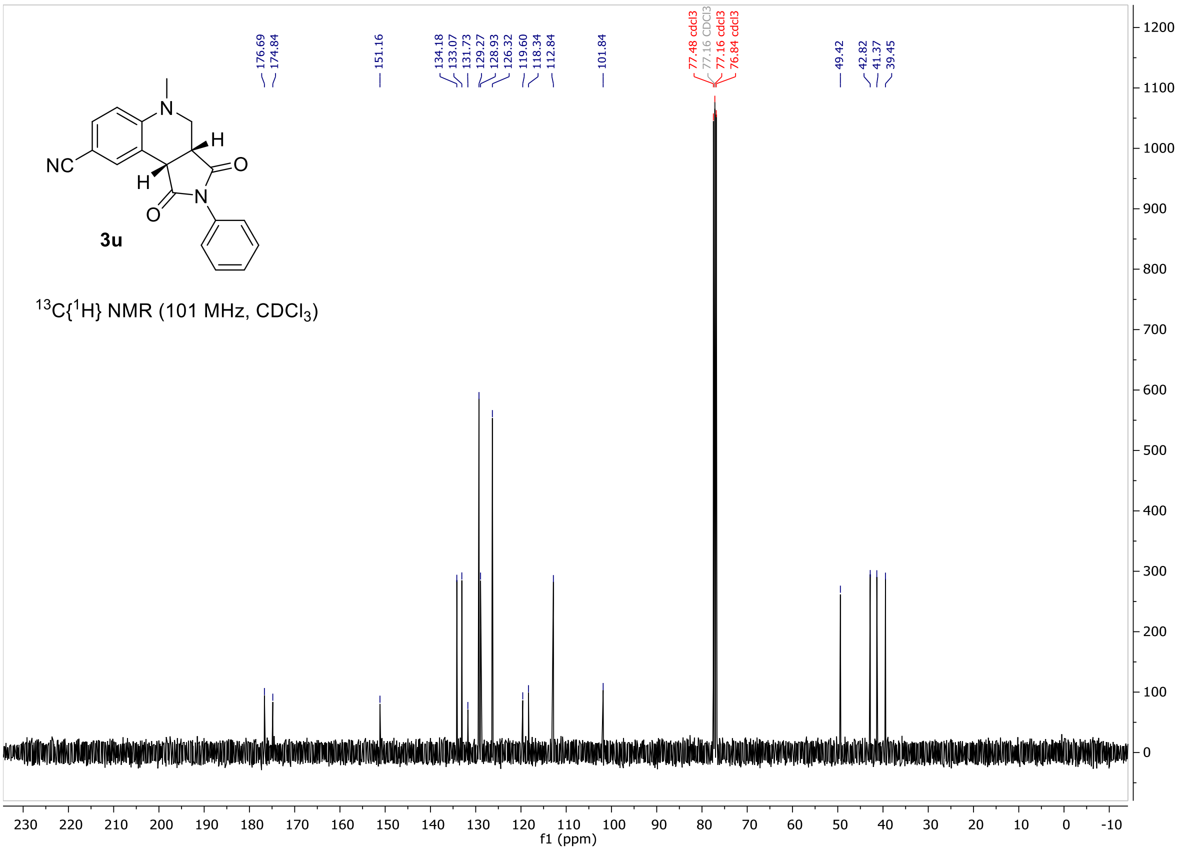

NC
3u
^{13}C{^{1}H} NMR (101 MHz, CDCl$_3$)
f1 (ppm)

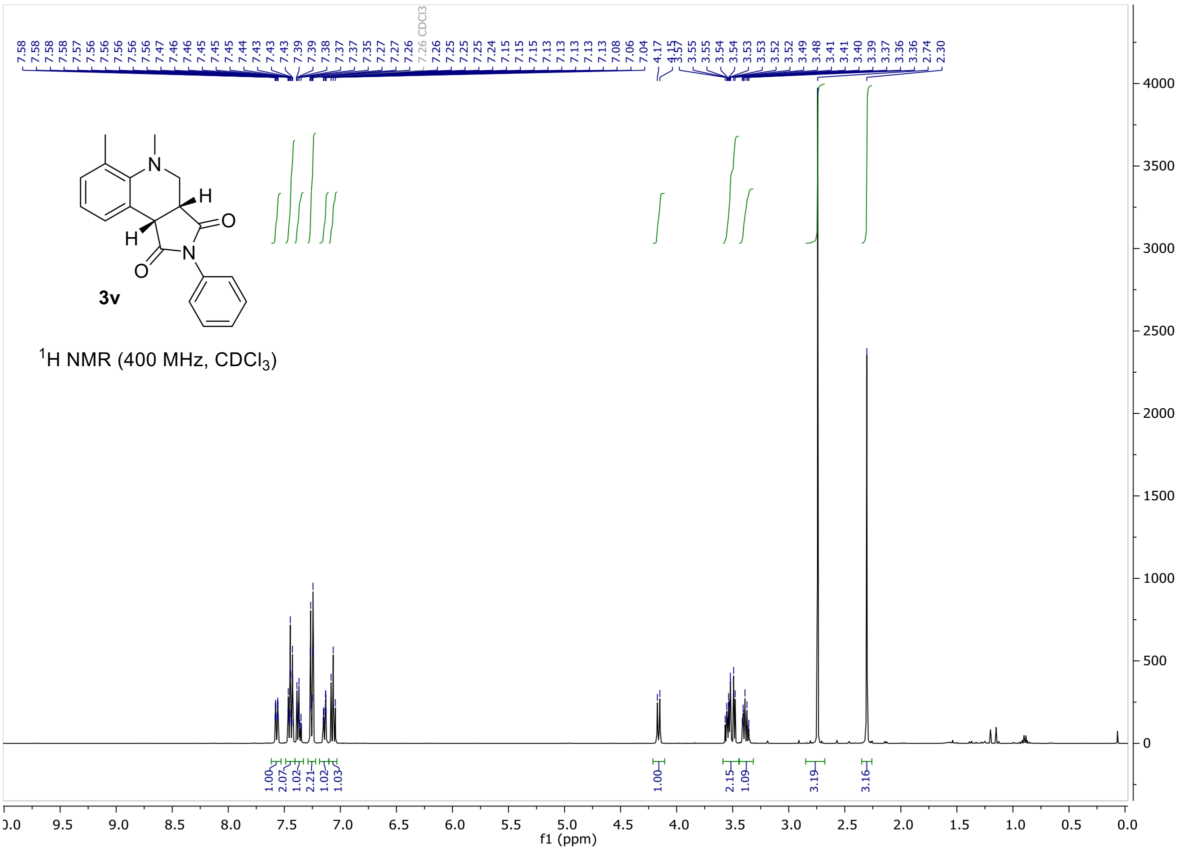
3v
^{1}H NMR (400 MHz, CDCl$_3$)
f1 (ppm)

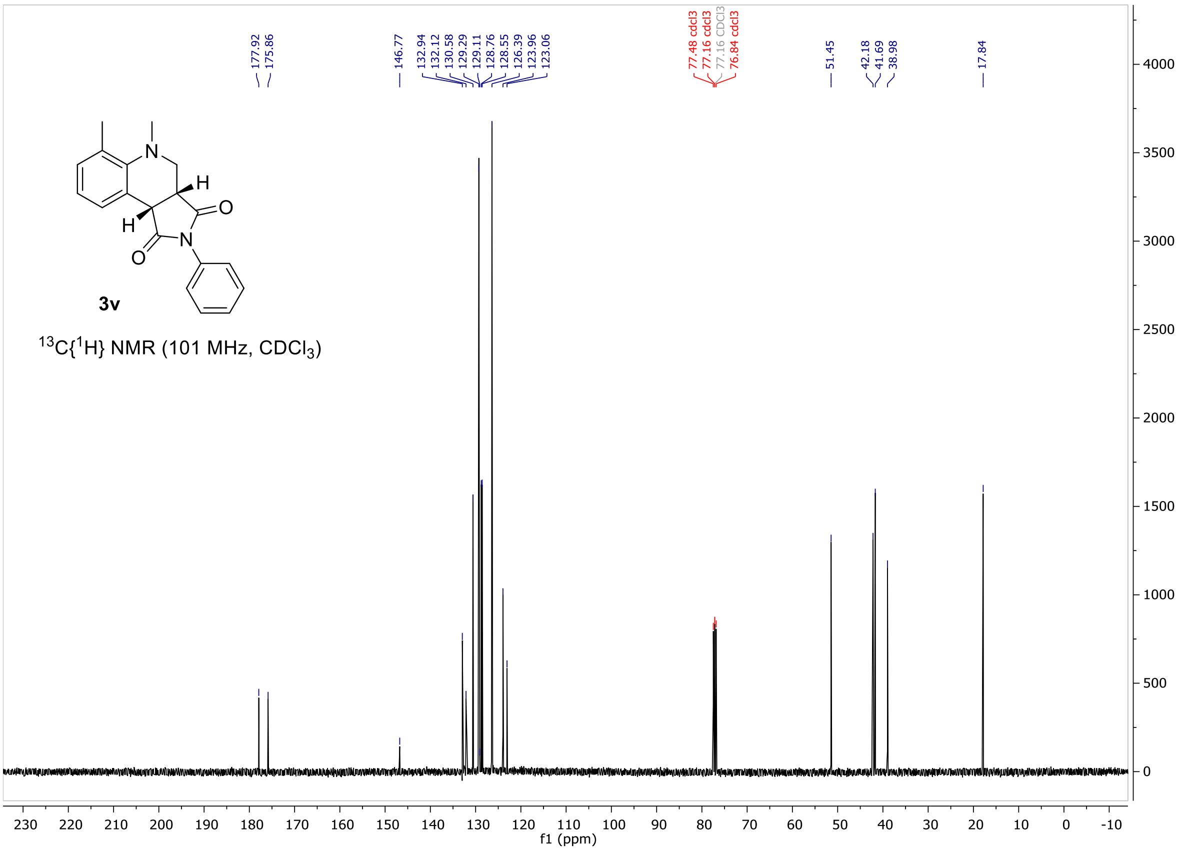
3v
^{13}C{^{1}H} NMR (101 MHz, CDCl$_3$)
f1 (ppm)

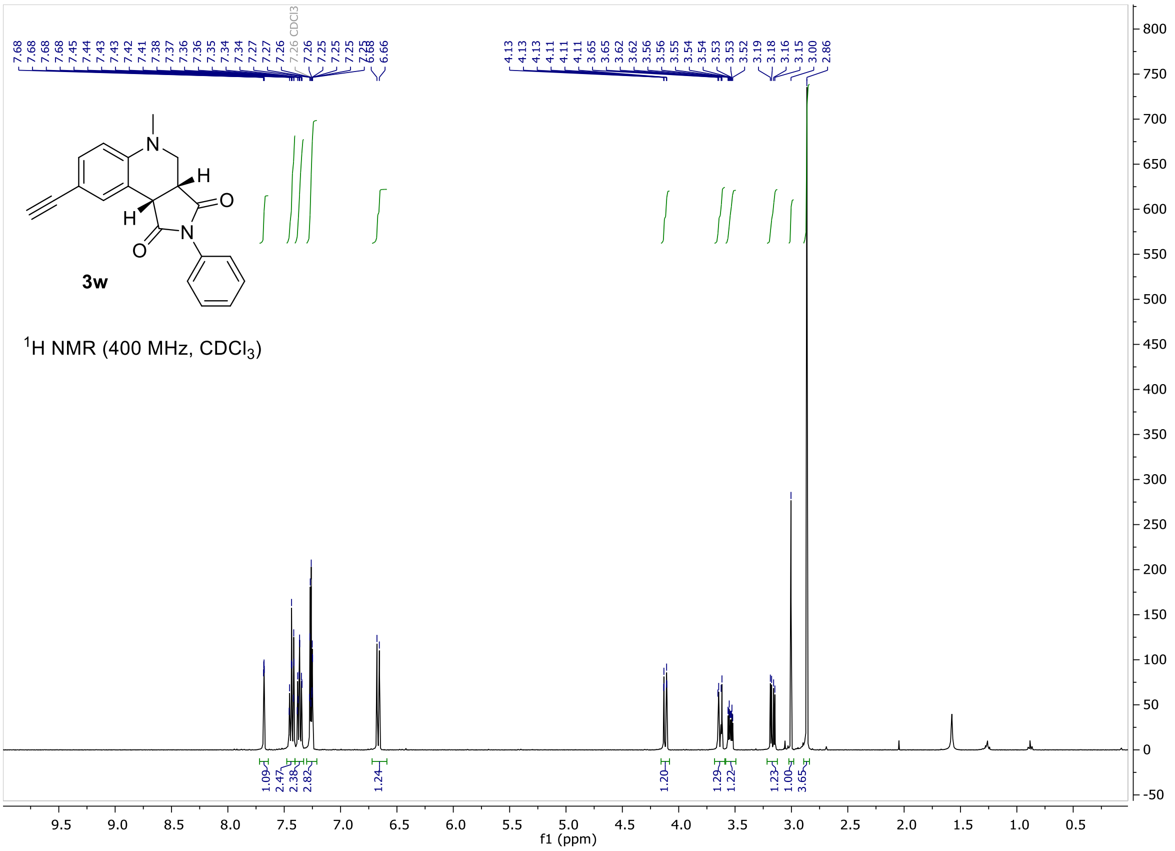
3w
^{1}H NMR (400 MHz, CDCl$_3$)
f1 (ppm)

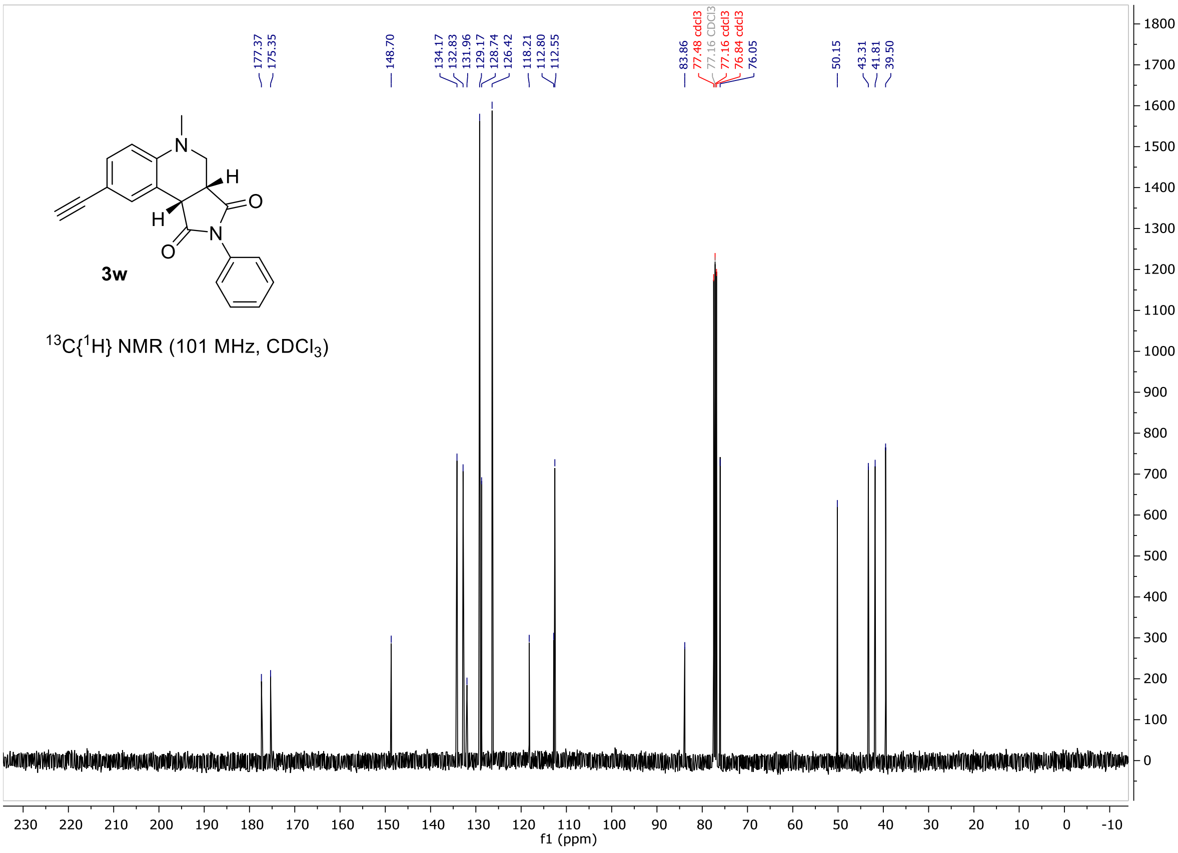
3w
^{13}C{^{1}H} NMR (101 MHz, CDCl$_3$)
f1 (ppm)

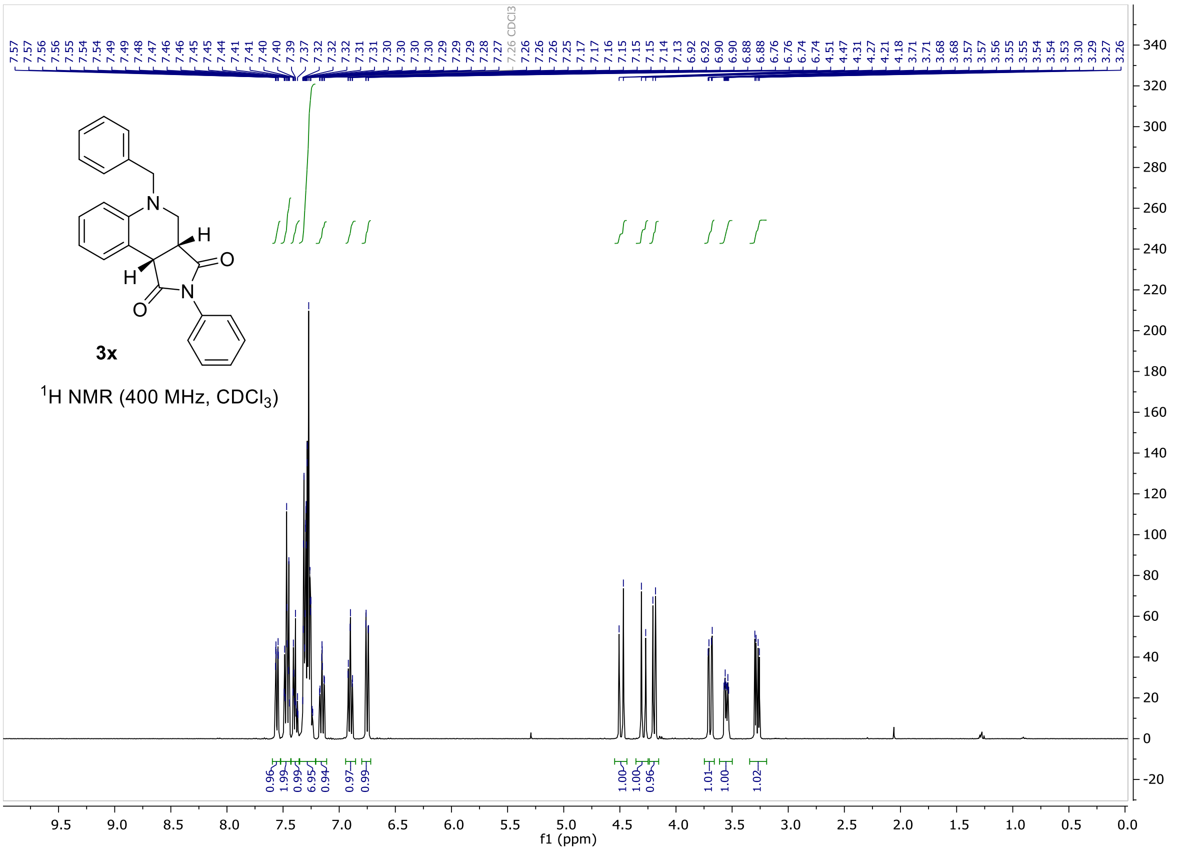
3x
^{1}H NMR (400 MHz, $CDCl_3$)
f1 (ppm)

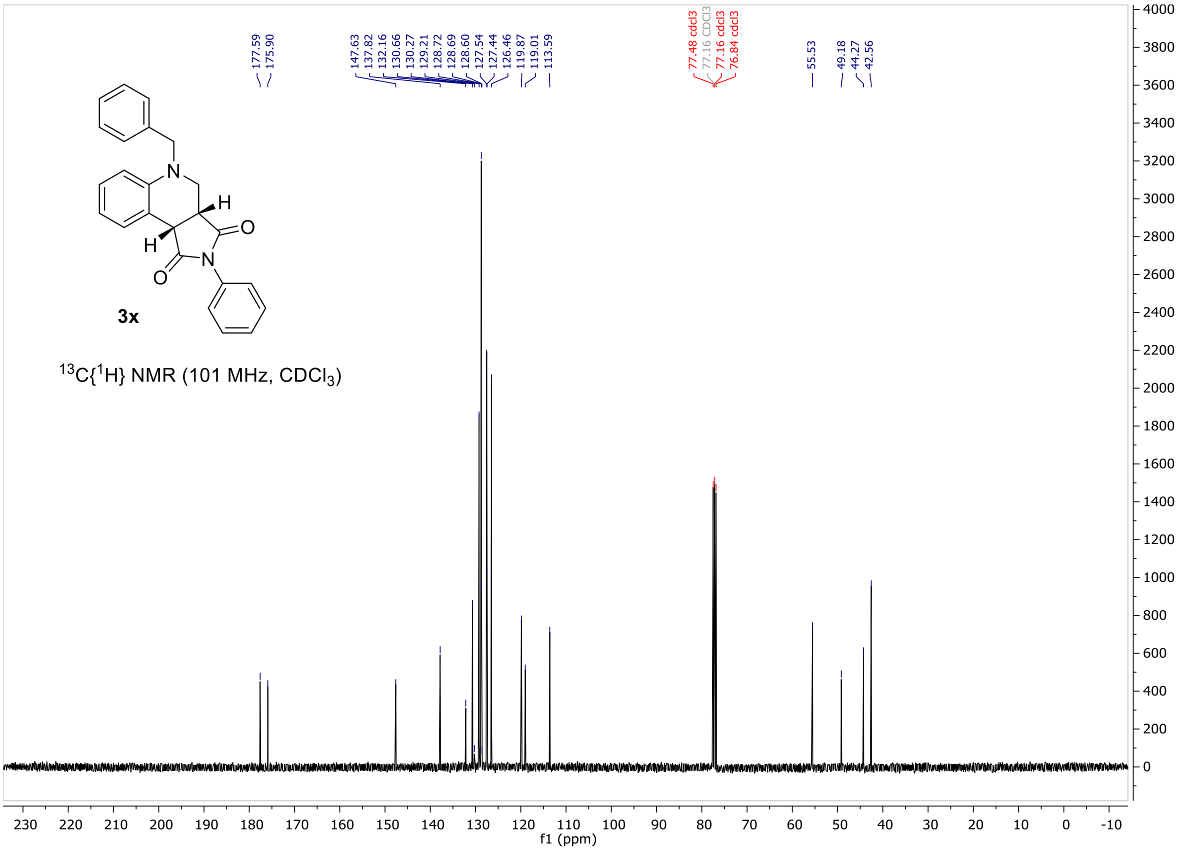
3x
$^{13}C\{^{1}H\}$ NMR (101 MHz, $CDCl_3$)
177.59
175.90
147.63
137.82
132.16
130.66
130.27
129.21
128.72
128.69
128.60
127.54
127.44
126.46
119.87
119.01
113.59
77.48 cdcl3
77.16 CDCl3
77.16 cdcl3
76.84 cdcl3
55.53
49.18
44.27
42.56
f1 (ppm)

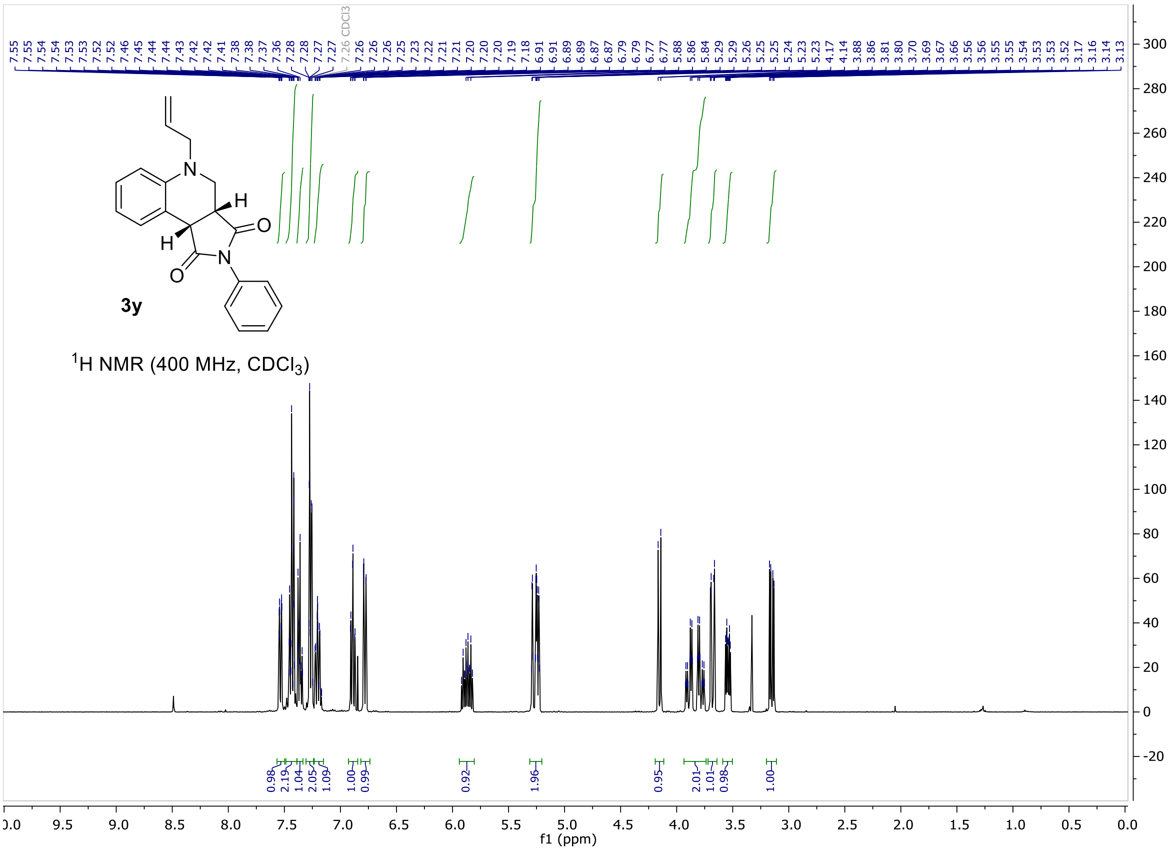
3y
^{1}H NMR (400 MHz, CDCl$_3$)
f1 (ppm)

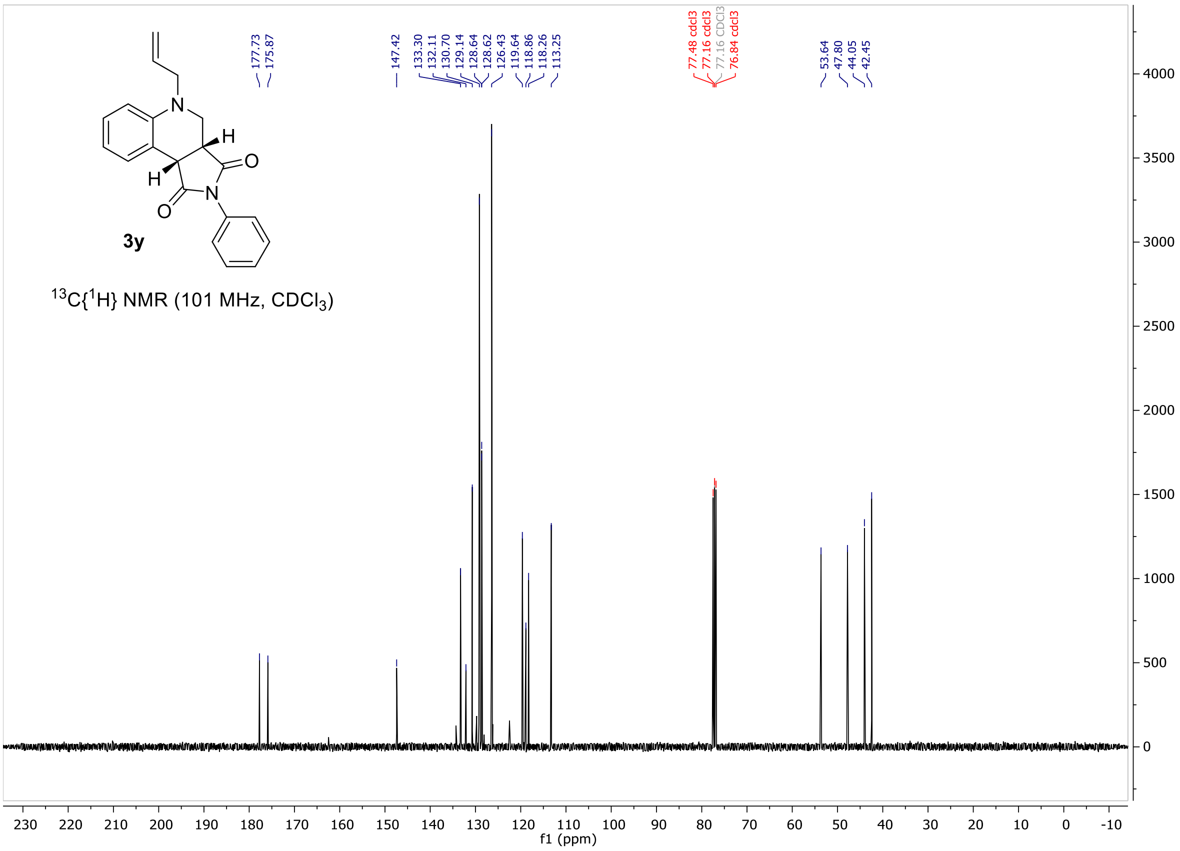
3y
^{13}C{^{1}H} NMR (101 MHz, CDCl$_3$)
177.73
175.87
147.42
133.30
132.11
130.70
129.14
128.64
128.62
126.43
119.64
118.86
118.26
113.25
77.48 cdcl3
77.16 cdcl3
77.16 CDCl3
76.84 cdcl3
53.64
47.80
44.05
42.45
f1 (ppm)

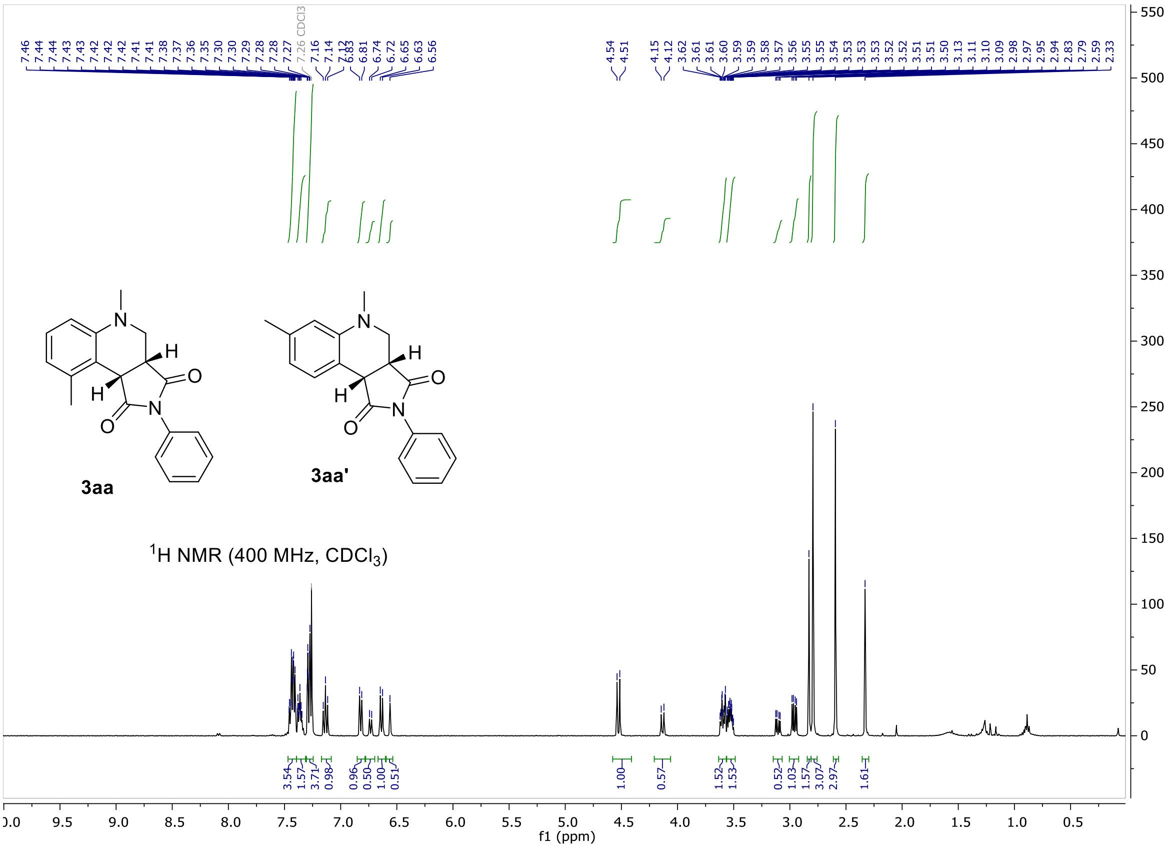

3aa
3aa'
^{1}H NMR (400 MHz, CDCl$_3$)
f1 (ppm)

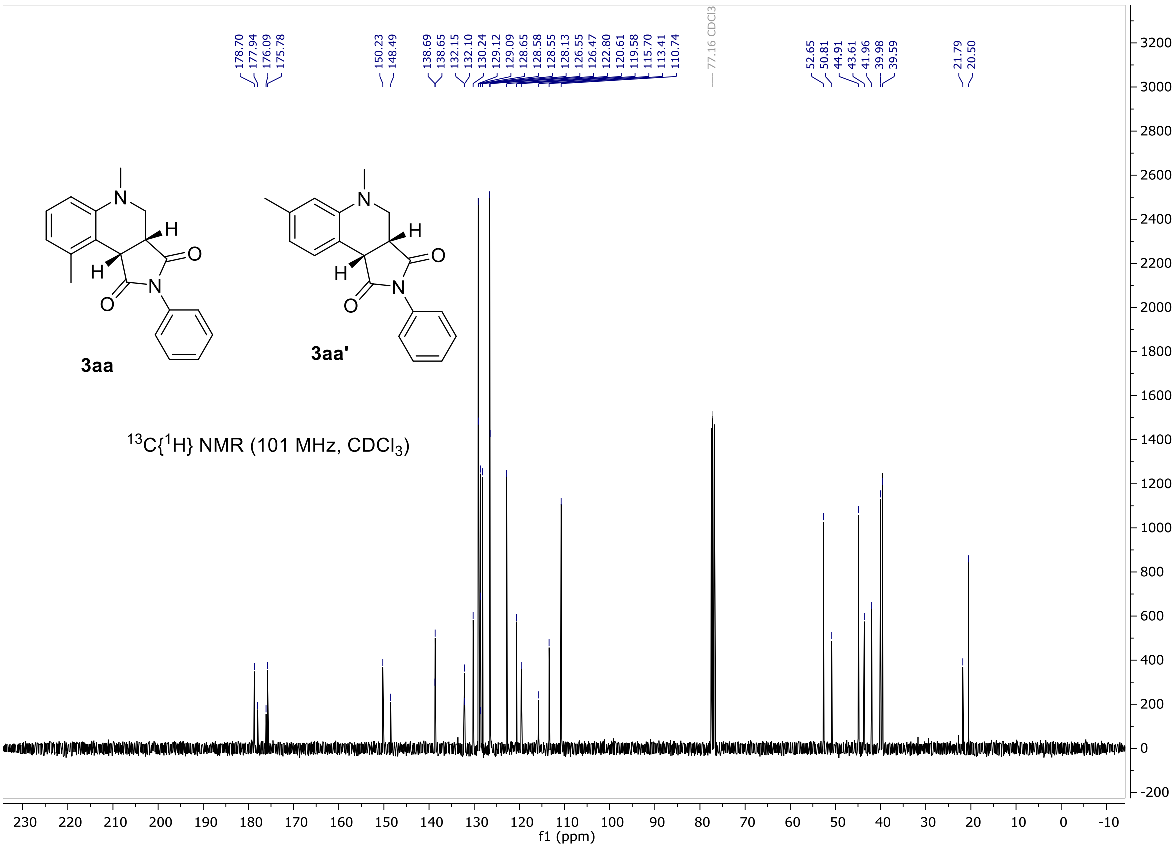

3aa
3aa'
^{13}C{^{1}H} NMR (101 MHz, CDCl$_3$)
f1 (ppm)

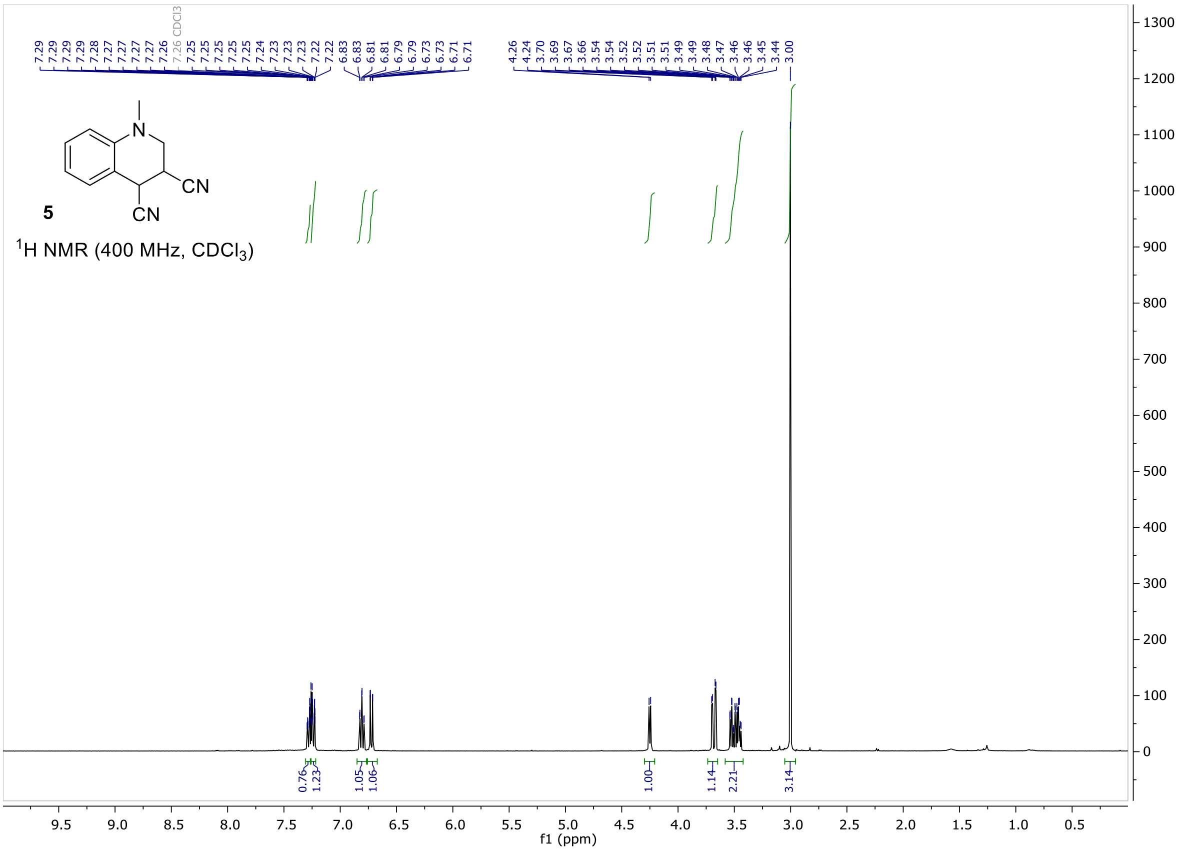
7.29 7.29 7.29 7.29 7.28 7.27 7.27 7.27 7.27 7.26 7.26 CDCl3 7.25 7.25 7.25 7.25 7.25 7.25 7.24 7.23 7.23 7.23 7.22 7.22 6.83 6.83 6.81 6.81 6.79 6.79 6.73 6.73 6.71 6.71
4.26 4.24 3.70 3.69 3.67 3.66 3.54 3.54 3.52 3.52 3.51 3.51 3.49 3.49 3.48 3.47 3.46 3.45 3.44 3.00
N
CN
CN
5
^{1}H NMR (400 MHz, CDCl$_3$)
0.76 1.23 1.05 1.06 1.00 1.14 2.21 3.14
f1 (ppm)

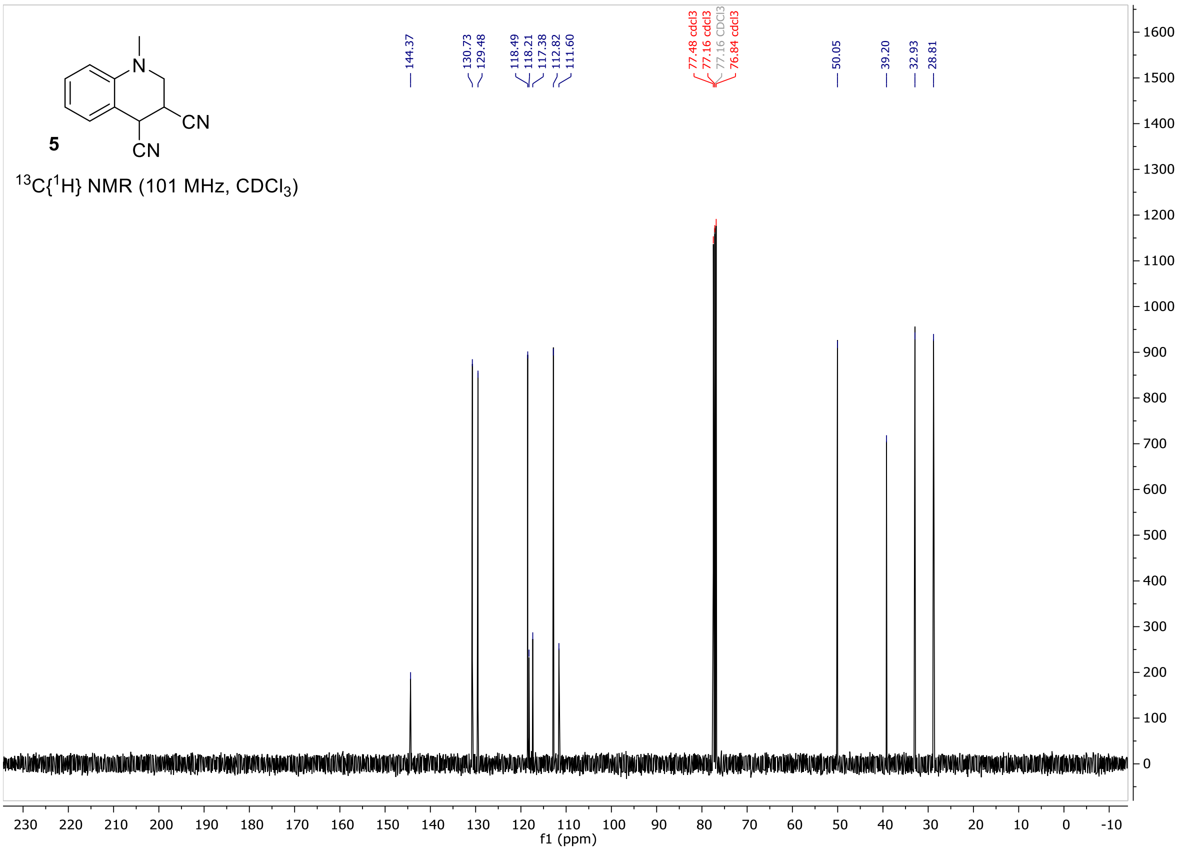
144.37
130.73 129.48
118.49 118.21 117.38 112.82 111.60
77.48 cdcl3 77.16 cdcl3 77.16 CDCl3 76.84 cdcl3
50.05
39.20
32.93
28.81
N
CN
CN
5
^{13}C{^{1}H} NMR (101 MHz, CDCl$_3$)
f1 (ppm)

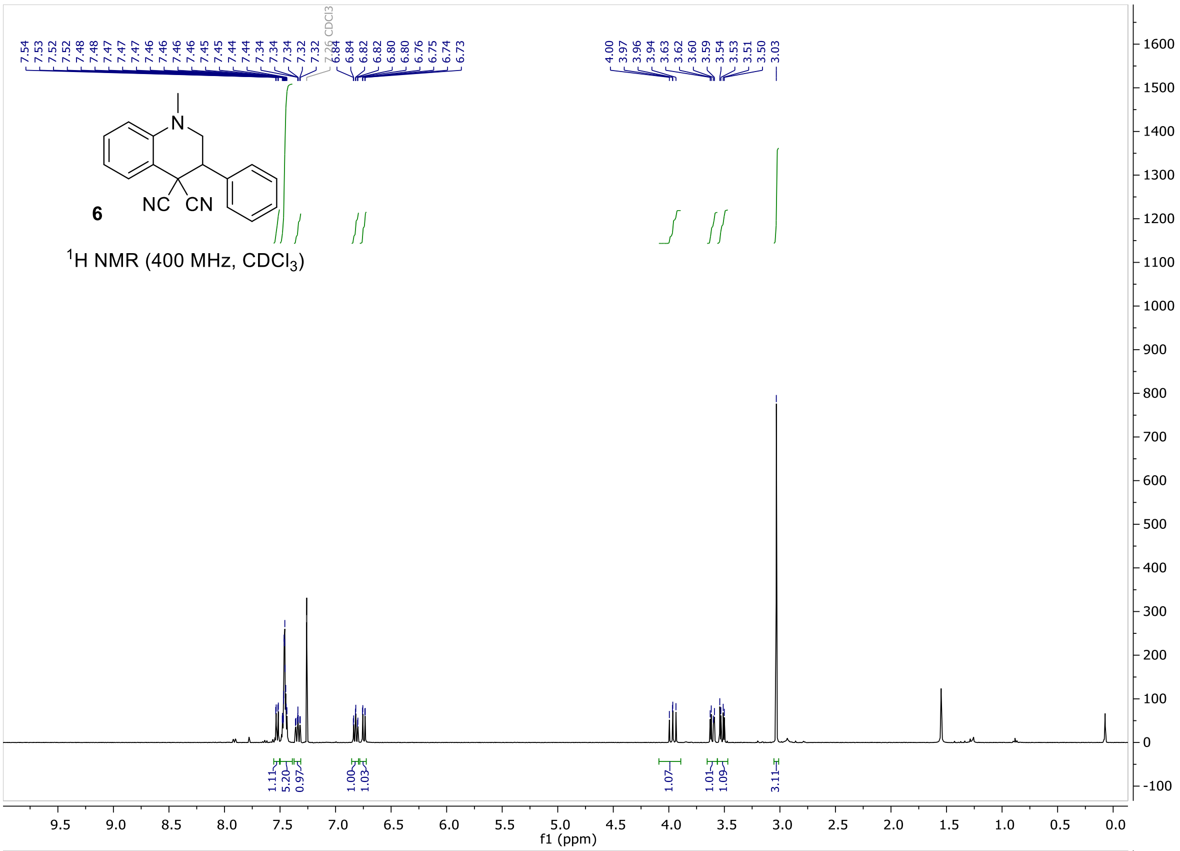
7.54 7.53 7.52 7.52 7.48 7.48 7.47 7.47 7.46 7.46 7.46 7.45 7.45 7.44 7.44 7.34 7.34 7.34 7.32 7.32 7.26 CDCl3 6.84 6.84 6.82 6.82 6.80 6.80 6.76 6.75 6.74 6.73
4.00 3.97 3.96 3.94 3.63 3.62 3.60 3.59 3.54 3.53 3.51 3.50 3.03
N
NC CN
6
1H NMR (400 MHz, CDCl3)
1.11 5.20 0.97 1.00 1.03 1.07 1.01 1.09 3.11
f1 (ppm)

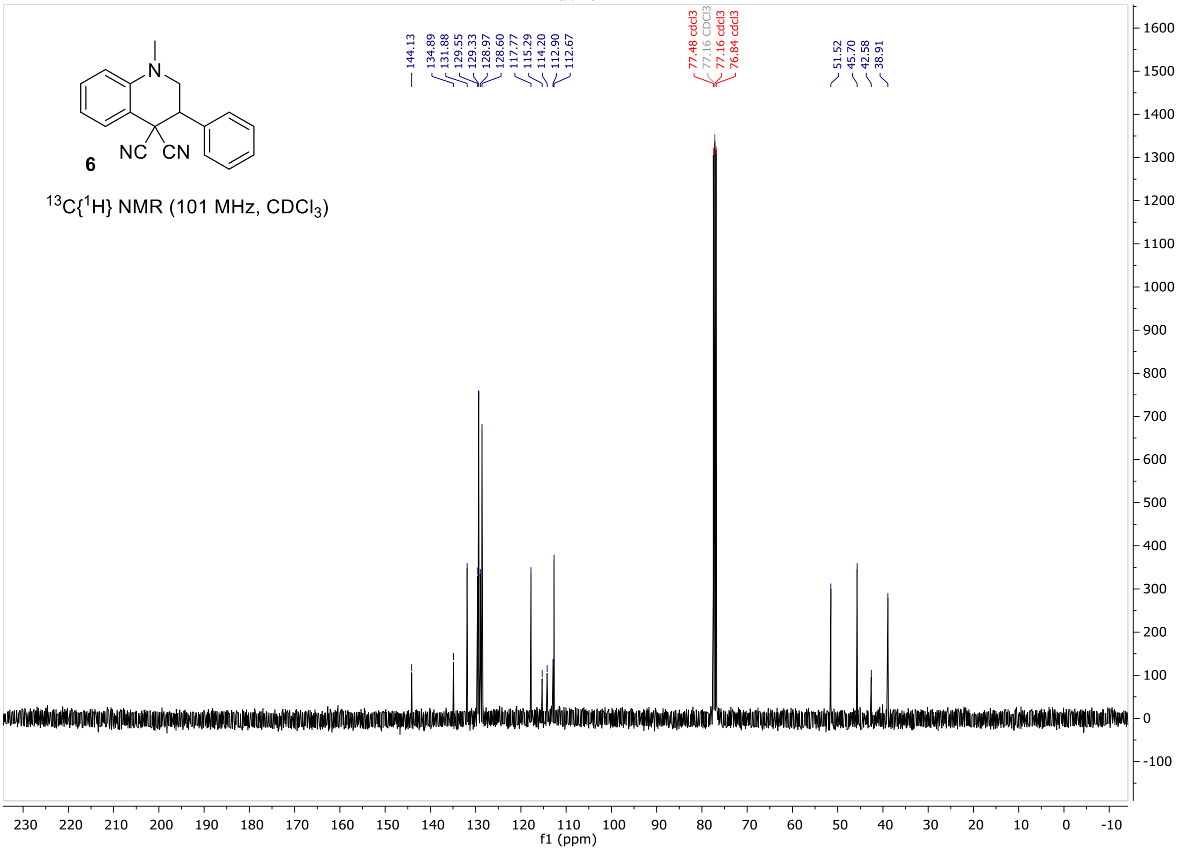
144.13 134.89 131.88 129.55 129.33 128.97 128.60 117.77 115.29 114.20 112.90 112.67
77.48 cdcl3 77.16 CDCl3 77.16 cdcl3 76.84 cdcl3
51.52 45.70 42.58 38.91
N
NC CN
6
13C{1H} NMR (101 MHz, CDCl3)
f1 (ppm)

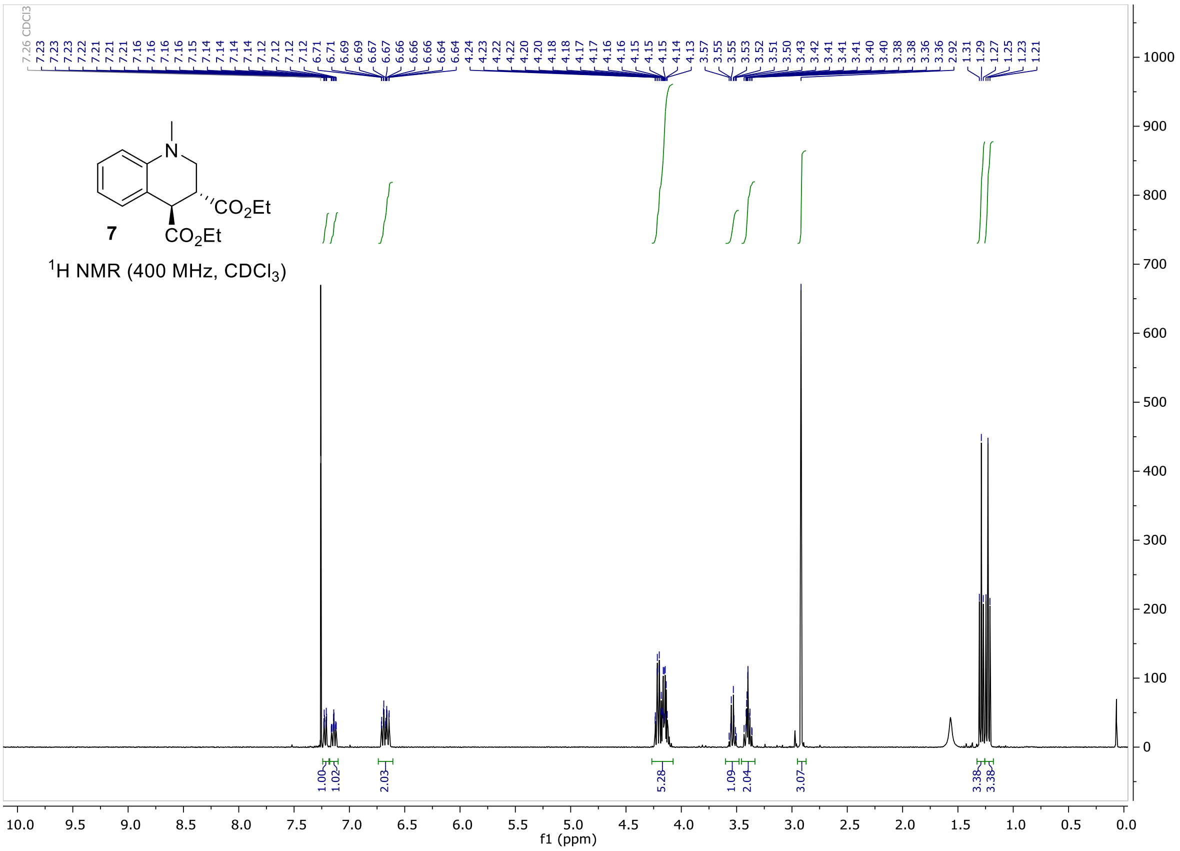
7
^1H NMR (400 MHz, $CDCl_3$)
f1 (ppm)

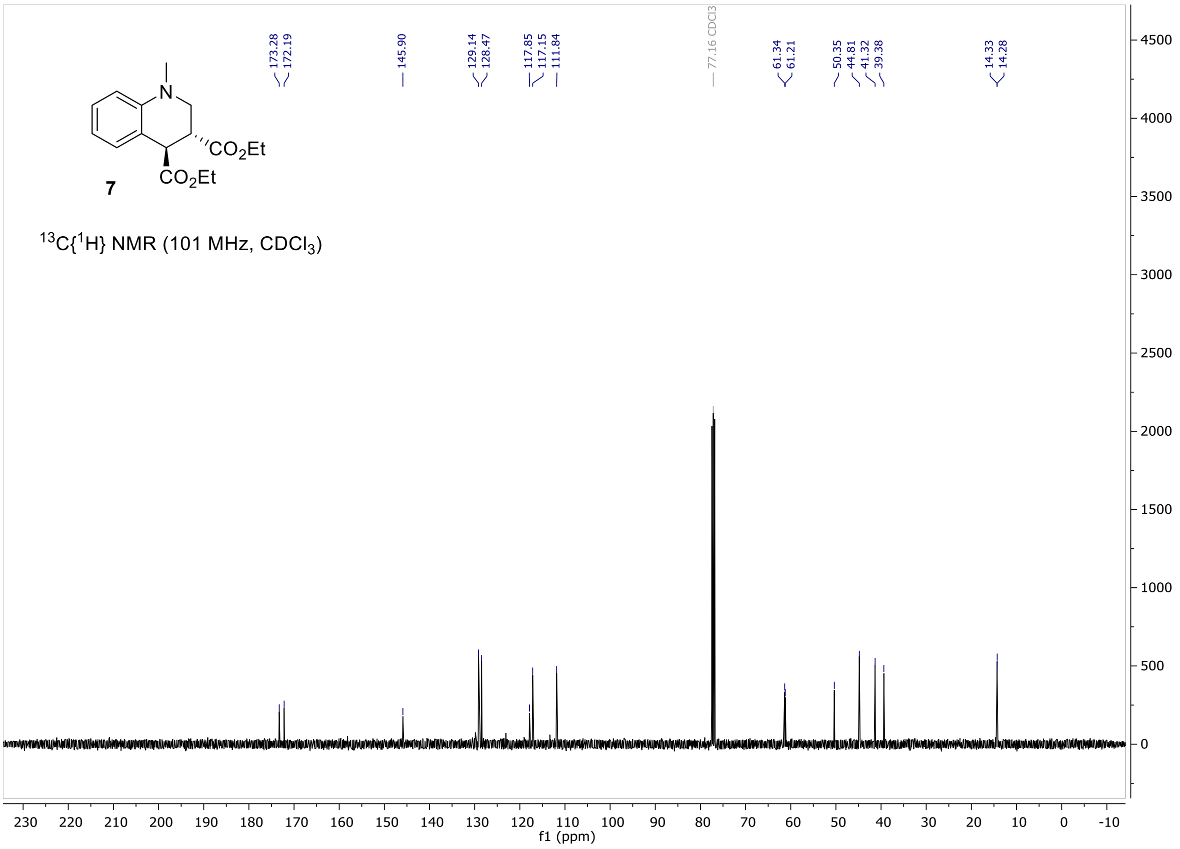
7
$^{13}C\{^1H\}$ NMR (101 MHz, $CDCl_3$)
f1 (ppm)
